# Design, synthesis, and in vitro antitumor activity of 6-aryloxyl substituted quinazoline derivatives

**DOI:** 10.55730/1300-0527.3373

**Published:** 2022-02-23

**Authors:** Meixia FAN, Lei YAO

**Affiliations:** School of Pharmacy, Key Laboratory of Molecular Pharmacology and Drug Evaluation (Yantai University), Ministry of Education, Collaborative Innovation Center of Advanced Drug Delivery System and Biotech Drugs in Universities of Shandong, Yantai University, Yantai, China

**Keywords:** Quinazolines, antitumor, epidermal growth factor receptor, synthesis

## Abstract

Quinazoline derivatives are a class of important antitumor drugs known as small molecule inhibitors that include epidermal growth factor receptor (EGFR) inhibitors. Based on the structure of poziotinib, a series of 6-aryloxyl substituted quinazoline derivatives were designed and synthesized. The in vitro antitumor activities of the compounds were evaluated by the 3-(4,5-dimethyl-thiazol-2-yl) 2,5-diphenyltetrazolium bromide (MTT) method using the human gastric cancer N87 (HER2), nonsmall-cell lung cancer H1975 (EGFR^T790M/L858R^), and A549 (EGFR^WT^) cell lines. The most promising compound **4m** exhibited potent antitumoral activities with IC_50_ values of 6.3 nM and 7.5 nM for N87 and H1975 cell lines, respectively. Meanwhile, it was less potent against A549 cancer cells with an IC_50_ value of 29.9 μM. The molecular docking results suggested that compound **4m** has different binding modes to the wild-type and mutated EGFR.

## 1. Introduction

Cancer is a common and frequently occurring disease that seriously endangers human health [1]. Therefore, the discovery and development of novel, potent, and less-toxic anticancer reagents are urgent and challenging tasks for medicinal chemists worldwide. Epidermal growth factor receptor (EGFR) is a well-known therapeutic target for anticancer drug discovery [2]. It is a member of the ERBB family receptor tyrosine kinases, which consists of four members: EGFR (also known as ERBB1/HER1), ERBB2/HER2/NEU, ERBB3/HER3, and ERBB4/HER4 [3]. EGFR is a membrane receptor tyrosine kinase that plays an important role in cell proliferation, invasion, metabolism, apoptosis, and survival [4–6]. Its gene overexpression, mutation, or amplification is a driver of many types of human malignancies, including glioblastoma, breast cancer, ovarian cancer, and lung cancer, especially nonsmall-cell lung cancers (NSCLCs) [7–9]. Thus, epidermal growth factor receptor tyrosine kinase inhibitors (EGFR-TKIs) can be used as antitumoral molecules to inhibit EGFR autophosphorylation and downstream signal transduction. To date, numerous small molecule inhibitors have been discovered, and some have achieved remarkable antitumor efficacies in clinic [10].

**Figure 1:**
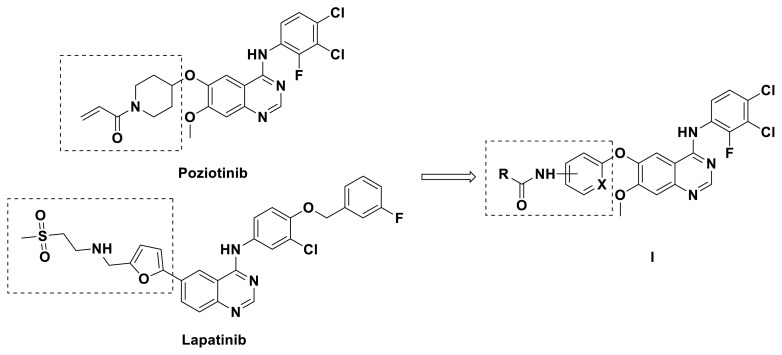
Structures and design strategy for 6-aryloxyl substituted quinazolines.

Quinazoline is a heterocyclic scaffold that possesses a wide range of biological activities [11–13] and 6-substituted quinazoline derivatives are important antitumor drugs that are used as small molecule inhibitors, especially as EGFR-TKIs [14]. The first-generation drugs, such as gefitinib [15], and erlotinib [16], have significant clinical response rates. However, acquired resistance through mutations such as T790M and T790M/L858R, rapidly arises and causes relapse after 9–14 months posttreatment [17,18]. To overcome this resistance, second and third-generation EGFR-TKIs, such as afatinib [19], osimertinib [20], were developed. The irreversible covalent bound with the Cys 797 confers enhanced sensitivity and selectivity to these TKIs; however, severe side effects are also observed due to their activity against the wild-type (WT) EGFR. Poziotinib (Figure 1, HM 781-36B) is a novel, potent, third-generation, irreversible pan-her inhibitor developed for the prevention and treatment of patients with breast cancer, gastric cancer, and NSCLC, including clinical limitations caused by an acquired mutation (EGFR T790M) [21]. However, severe toxicities, such as diarrhea and skin rash, hampered its clinical administration [22]. Although these side effects are partially ascribed to the toxic de-methyl metabolite, the strong and irreversible inhibition of EGFR is still believed to be the main reason [23]. To further optimize this candidate drug, a series of 6-aryloxyl substituted quinazoline derivatives, were synthesized, and their antitumor activities were screened using the N87 human gastric cancer cell line, and the H1975 and A549 nonsmall-cell lung cancer cell lines.

Initially, we reviewed the structures of quinazoline EGFR-TKIs and found that the 6-position of quinazoline is mostly substituted by aliphatic alkanes or cycloalkanes. For instance, gefitinib, erlotinib, icotinib, and vandetanib had the 6,7-dialkyloxyl moieties, but only lapatinib, a rigid aromatic ring (furan), was found in the 6-position. Thus, we presumed that the flexible alkyl chain was crucial for the molecule to have strong interactions with the enzyme. Based on the structures of poziotinib and lapatinib, a series of 6-aryloxyl substituted quinazolines (I, Figure 1) were designed by introducing benzene and pyridine rings. Meanwhile, in order to check the effect of the electron density of these aromatic rings on the antitumoral activity of the drug, an electron-donating methoxy group was also introduced to the 2′- or 4′-position. In previously published studies [24–26], the introduction of small substituents at the end of the acrylamide side chain could change the activity of the reaction between the acrylamide warhead and the amino acid residue Cys 797 in the back pocket. So, four acrylic acid derivatives, including methacrylic acid, but-2-enoic acid, and 3-methylbut-2-enoic acid were used to afford the acrylamides in this article.

## 2. Results and discussion

### 2.1. Chemistry

The structures and preparation of compounds **4a**–**4t** are described in Scheme. Starting from a commercially available 6-hydroxyl-7-methoxy-4-arylaminoquinazoline (**1**), the final target molecules were obtained in three steps. Firstly, treatment of compound **1** with 1- or 2-fluoronitrobenzene at 50 °C generated the intermediates **2a**–**2e**. Secondly, the reduction of the **2a**–**2e** nitro group by the classical Fe/NH_4_Cl or H_2_/Pd-C method generated the amine compounds **3a**–**3e**. Finally, the amide formation reaction of the compounds **3a**–**3e** with acyl chlorides generated the compounds **4a**–**4t** in a moderate yield.

### 2.2 In vitro antitumor activity assay

To determine their antiproliferative activity, all synthesized compounds (**4a**–**4t**) were tested on the EGFR overexpressing cell lines, N87 (human gastric cancer), A549 (EGFR^WT^), and H1975 (EGFR^L858R/T790M^), using the 3-(4,5-dimethylthiazolyl-2)-2, 5-diphenyltetrazolium bromide (MTT) assay. The results were expressed as IC_50_ values and summarized in Table.

The antitumoral activities of these compounds were found to be less potent than those of poziotinib. Meanwhile, they were more potent for N87 and H1975 cancer cells compared to that for A549 cancer cells. Compound **4m** showed antitumoral activities against N87 gastric cancer cells with an IC_50_ value of 6.3 nM, while against H1975 lung cancer cells, the IC_50_ value was 7.5 nM. A preliminary summary of the structure-activity relationship of the 6-aryloxyl substituted quinazoline derivatives is summarized as follows: 1. Compounds with substitution (R_1_) on the acryloyl group generally possessed less antitumoral activity. For example, compound **4d** (IC_50_ = 9.6 and 21.3 nM) was more potent than compound **4i** (IC_50_ = 25.2 and 53.1 nM) when used for the treatment of N87 and H1975 cancer cells. Compound **4n** (IC_50_ = 27.3 and 41.5 nM) was more potent than compound **4s** (IC_50_ = 97.2 and 173.3 nM) when used for the treatment of N87 and H1975 cancer cells; 2. Compounds with benzene substitution at C-6 position exhibited better IC_50_ values than those with a pyridine substitution. For example, compound **4c** (IC_50_ = 12.3, 35.4 nM and 38.6 μM) was more potent than compound **4r** (IC_50_ = 83.6, 125.3 nM and 50.6 μM) when used for treatment of N87, H1975 and A549 cancer cells, and compound **4d** (IC_50_ = 9.6, 21.3 nM and 30.2 μM) was more potent than compound **4s** (IC_50_ = 97.2, 173.3 nM and 55.3 μM); 3. The methoxyl substitution on the phenyl ring had little effect on the antitumoral activity of the compounds. Compounds **4c** (IC_50_ = 12.3, 35.4 nM and 38.6 μM) and **4d** (IC_50_ = 9.6, 21.3 nM and 30.2 μM) had similar antitumoral activities against N87, H1975 and A549 cancer cells. Compound **4r** (83.6, 125.3 nM and 50.6 μM) and **4s** (IC_50_ = 97.2, 173.3 nM and 55.3 μM) exhibited comparable activities. 4. Compounds with para-acrylamide were more potent than those with ortho-acrylamide. For instance, compound **4a** (IC_50_ = 4.7, 12.3 nM) was more potent than compound **4b** (IC_50_ = 46.4, 88.5 nM) when used for the treatment of N87 and H1975 cancer cells, and compound **4k** (IC_50_ = 5.3, 9.5 nM) exhibited a better antitumoral activity than that of compound **4l** (IC_50_ = 37.5, 24.6 nM).

### 2.3. Molecular docking study

**Figure 2:**
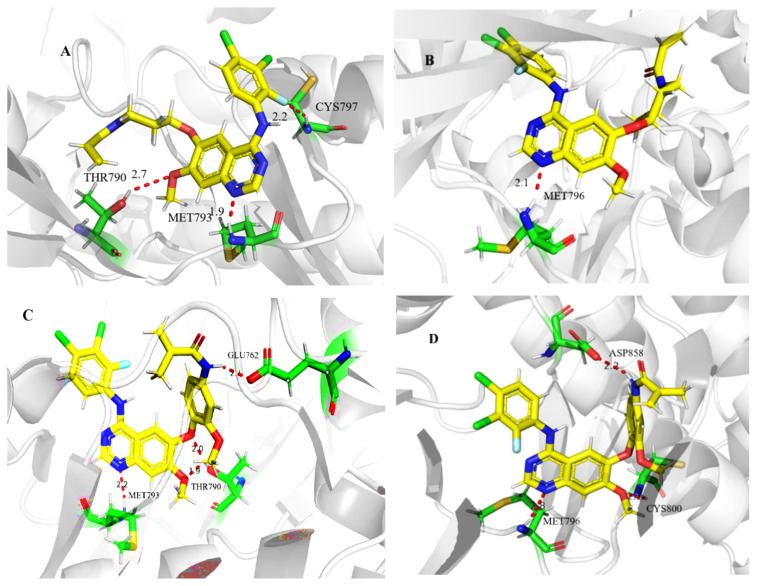
The results of the molecular docking. **A** and **B** are predicted binding model of poziotinib with 4ZAU and 4LRM; **C** and **D** are predicted binding model of compound **4m** with 4ZAU and 4LRM.

To better understand how these compounds interact with the target proteins, a molecular docking study was performed using the SURFLEX-DOCK module of the SYBYL package version. Poziotinib and compound **4m** were selected for the molecular docking study. The proteins selected were EGFR^WT^ protein (PDB ID code: 4ZAU) and EGFR D770_N771 insNPG protein (PDB ID code: 4LRM). The results of docking analysis were shown in Figure 2. Poziotinib and compound **4m** adopted a U-shape in the kinase domain and combined with the EGFR kinase. The amino-pyrimidine group formed a hydrogen bound with the amino acid residue Met 793 in the hinged region and the side chain amine penetrated the solvent region. With the wild-type EGFR, compound **4m** formed strong two-dentate hydrogen bounds with Thr 790 because its hydrogen bonds were shorter than those of poziotinib (1.9 Å vs. 2.7 Å). In the mutated EGFR, the amino-pyrimidine group of the compound **4m** formed hydrogen bounds with the amino acid residue Met 796 in the hinged region. Poziotinib bound the protein more tightly than compound **4m** because the hydrogen bonds were shorter than those of **4m** (2.1 Å vs. 2.8 Å). The molecular docking results also suggested that compound **4m** had a different binding mode with the wild-type when compared with that of the mutated EGFR. This could be partially explained by its higher potency when used in the treatment of N87 and H1975 cancer cells and when compared with that of A549 cancer cells. Compound **4m** was found to have lower binding scores with the wild-type EGFR compared with those with the mutated EGFR. The docking results also showed that the amino-pyrimidine group served as an indispensable anchor for hydrogen bond interactions with Met 793 or Met 796, and that the transformation direction of side chain was of certain significance.

### 2.4. Conclusion

A series of 6-aryloxyl substituted quinazoline derivatives were designed and synthesized, and their antitumoral activity was screened by the MTT assay using N87, H1975 and A549 cell lines. The most promising compound **4m** showed a potent antitumoral activity against N87 and H1975 cells with the IC_50_ values of 6.3 nM and 7.5 nM. Although it was less potent than the lead compound poziotinib, more investigations on compound **4m** toxicity and side effects are required. To the best of our knowledge, the intolerance of poziotinib is due to the toxicity during its clinical usage. We attempted to reduce the toxicity of poziotinib by partially sacrificing its antitumoral activity. Despite that, there is still an unmet medical need to develop novel small molecule EGFR-TKIs or therapeutic approaches to overcome multipoint mutations in EGFR [27].

## 3. Experiments

### 3.1. Material and instruments

The chemicals were purchased from Shanghai Aladdin Biochemical Technology Co., Ltd. China. The 4-(3,4-dichloro-2-fluorophenylamino)-7-methoxyquinazolin-6-ol (**1**) was bought from Suzhou NMT Biotech Co., Ltd. China. The NMR spectra of the intermediates and final products in the deuterated solvent were detected on a Bruker 400/101 MHz spectrometer. The high-resolution mass spectra (HRMS) were recorded on an Agilent 6520 ESI/TOF mass spectrometer. The uncorrected melting points (MP) were recorded on a Büchi B-540 melting point apparatus. Flash column chromatographic separation was achieved using a silica gel from Qingdao Ocean Chemical (200–300 mesh) with a particle size from 54 to 74 μm using dichloromethane and methanol (or ethyl acetate) as eluents. The analytical TLC was carried out on a Merck precoated silica gel 60 GF-254 using 0.25-mm-thick TLC plates.

### 3.2. General experimental procedures and physical data of compounds 4

Sodium carbonate (0.28 g, 2.69 mmol) was added to a solution of compound **3** (0.3 g, 0.67 mmol) in tetrahydrofuran (THF) (8 mL), and under violent stirring. The reaction solution was cooled to 0–5 °C, and a solution of acryloyl chloride (2.34 mmol) in THF (8 mL) was added through an addition funnel within 30 min reaction mixture was stirred for 60 min at room temperature till TLC showed the completion of the reaction. Water (20 mL) was added to the reaction mixture and the aqueous solution was extracted with ethyl acetate. The organic layer was combined, dried, and evaporated. The residue was purified by silica gel column chromatography (dichloromethane/methanol = 50:1 – 10:1) and generated compound **4** as a pale-yellow solid.

#### *N*-(4-((4-((3,4-Dichloro-2-fluorophenyl)amino)-7-methoxyquinazolin-6-yl)oxy) phenyl)acrylamide (4a)

A pale-yellow solid (187 mg, 56% yield); m.p: 222–224 °C; ^1^H NMR (400 MHz, DMSO-*d*_6_) *δ* 10.14 (s, 1H), 9.76 (s, 1H), 8.46 (s, 1H), 8.05 (s, 1H), 7.66 (d, *J* = 9.1 Hz, 2H), 7.53 (s, 2H), 7.39 (s, 1H), 6.98 (d, *J* = 9.1 Hz, 2H), 6.37–6.45 (m, 1H), 6.25 (d, *J* = 2.1 Hz, 1H), 5.73 (dd, *J* = 10.1, 2.1 Hz, 1H), 3.94 (s, 3H). ^13^C NMR (101 MHz, DMSO - *d*_6_) *δ* 162.98, 157.29, 156.22, 154.44, 154.04, 154.01, 153.08, 144.87, 134.49, 131.96, 126.94, 126.50, 125.30, 125.26, 121.00, 119.67, 119.48, 117.27, 113.00, 108.83, 108.40, 56.20. HRMS (ESI): m/z calcd for C_24_H_17_Cl_2_FN_4_O_3_: 499.0734 [M+H]^+^; found: 499.0738.

#### *N*-(2-((4-((3,4-Dichloro-2-fluorophenyl)amino)-7-methoxyquinazolin-6-yl)oxy) phenyl)acrylamide (4b)

A pale-yellow solid (214 mg, 64% yield); m.p: 223–225 °C; ^1^H NMR (400 MHz, DMSO-d_6_) δ 9.80 (s, 2H), 8.48 (s, 1H), 8.16–8.22 (m, 2H), 7.53 (s, 2H), 7.43 (s, 1H), 7.01–7.10 (m, 2H), 6.70–6.83 (m, 2H), 6.25–6.31 (m, 1H), 5.72–5.76 (m, 1H), 3.94 (s, 3H). ^13^C NMR (101 MHz, DMSO-d_6_) δ 163.71, 157.37, 156.47, 154.46, 151.94, 149.43, 148.48, 144.07, 131.97, 128.35, 128.12, 127.01, 125.37, 125.32, 124.85, 123.13, 122.63, 119.74, 119.55, 115.42, 114.43, 108.87, 108.70, 56.28. HRMS (ESI): m/z calcd for C_24_H_17_Cl_2_FN_4_O_3_: 499.0734 [M+H]^+^; found: 499.0730.

#### *N*-(4-((4-((3,4-Dichloro-2-fluorophenyl)amino)-7-methoxyquinazolin-6-yl)oxy)-3-methoxyphenyl)acrylamide (4c)

A pale-yellow solid (184 mg, 52% yield); m.p: 191–193 °C; ^1^H NMR (400 MHz, DMSO-*d*_6_) *δ* 10.22 (s, 1H), 9.64 (s, 1H), 8.40 (s, 1H), 7.67 (d, *J* = 2.2 Hz, 1H), 7.63 (s, 1H), 7.51 (s, 2H), 7.34 (s, 1H), 7.25 (dd, *J* = 8.7, 2.3 Hz, 1H), 6.98 (d, *J* = 8.7 Hz, 1H), 6.43 (dd, *J* = 17.0, 10.1 Hz, 1H), 6.26 (dd, *J* = 17.0, 2.1 Hz, 1H), 5.75 (dd, *J* = 10.1, 2.1 Hz, 1H), 3.98 (s, 3H), 3.77 (s, 3H). ^13^C NMR (101 MHz, DMSO-*d*_6_) *δ* 163.06, 157.03, 155.04, 153.51, 150.37, 146.75, 139.65, 136.43, 131.82, 127.26, 126.86, 125.25, 125.21, 120.24, 119.61, 119.42, 111.70, 108.58, 108.09, 107.72, 105.02, 56.09, 55.57. HRMS (ESI): m/z calcd for C_25_H_19_Cl_2_FN_4_O_4_: 529.0840 [M+H] ^+^; found: 529.0838.

#### *N*-(2-((4-((3,4-Dichloro-2-fluorophenyl)amino)-7-methoxyquinazolin-6-yl)oxy)-5-methoxyphenyl)acrylamide (4d)

A pale-yellow solid (205 mg, 58% yield) ; m.p: 225–227 °C; ^1^H NMR (400 MHz, DMSO-*d*_6_) *δ* 9.74 (s, 2H), 8.45 (s, 1H), 7.92–7.96 (m, 2H), 7.53 (s, 2H), 7.39 (s, 1H), 6.77 (s, 2H), 6.65 (s, 1H), 6.23–6.27 (m, 1H), 5.73 (d, *J* = 11.1 Hz, 1H), 3.96 (s, 3H), 3.73 (s, 3H). ^13^C NMR (101 MHz, DMSO-*d*_6_) *δ* 163.76, 156.12, 154.80, 154.78, 154.13, 153.61, 145.68, 141.43, 131.92, 129.70, 129.66, 127.14, 127.12, 125.36, 125.33, 125.28, 122.34, 119.19, 117.54, 109.47, 108.54, 108.53, 99.48, 56.21, 55.37. HRMS (ESI): m/z calcd for C_25_H_19_Cl_2_FN_4_O_4_: 529.0840 [M+H] ^+^; found: 529.0839.

#### *N*-(2-((4-((3,4-Dichloro-2-fluorophenyl)amino)-7-methoxyquinazolin-6-yl)oxy) pyridin-4-yl)acrylamide (4e)

A pale-yellow solid (181 mg, 54% yield); m.p: 238–240 °C; ^1^H NMR (400 MHz, DMSO-*d*_6_) *δ* 10.09 (s, 1H), 9.89 (s, 1H), 8.58 (dd, *J* = 7.9, 1.5 Hz, 1H), 8.50 (s, 1H), 8.34 (s, 1H), 7.76 (dd, *J* = 4.9, 1.7 Hz, 1H), 7.57 (d, *J* = 7.7 Hz, 2H), 7.40 (s, 1H), 7.12 (dd, *J* = 7.9, 4.9 Hz, 1H), 6.85 (dd, *J* = 17.0, 10.2 Hz, 1H), 6.32 (dd, *J* = 17.0, 2.1 Hz, 1H), 5.79 (dd, *J* = 10.2, 2.1 Hz, 1H), 3.87 (s, 3H). ^13^C NMR (101 MHz, DMSO-*d*_6_) *δ* 164.26, 157.46, 156.72, 154.57, 153.69, 151.92, 149.91, 142.09, 141.05, 131.53, 130.52, 128.89, 127.60, 126.98, 126.96, 125.33, 125.29, 122.31, 118.90, 116.54, 108.74, 108.26, 56.24. HRMS (ESI): m/z calcd for C_23_H_16_Cl_2_FN_5_O_3_: 500.0687 [M+H] ^+^; found: 500.0685.

#### (*E*)-*N*-(4-((4-((3,4-Dichloro-2-fluorophenyl)amino)-7-methoxyquinazolin-6-yl) oxy)phenyl)but-2-enamide (4f)

A pale-yellow solid (175 mg, 51% yield); m.p: 249–251 °C; ^1^H NMR (400 MHz, DMSO-*d*_6_) *δ* 10.14 (s, 1H), 9.86 (s, 1H), 8.45 (s, 1H), 8.09 (s, 1H), 7.69 (s, 1H), 7.67 (s, 1H), 7.53 (s, 2H), 7.38 (s, 1H), 6.97 (s, 1H), 6.95 (d, *J* = 2.1 Hz, 1H), 6.72–6.81 (m, 1H), 6.16 (dd, *J* = 15.2, 1.7 Hz, 1H), 3.93 (s, 3H), 1.84 (dd, *J* = 6.9, 1.6 Hz, 3H). ^13^C NMR (101 MHz, DMSO-*d*_6_) *δ* 163.27, 157.24, 156.17, 154.15, 152.77, 149.00, 144.92, 139.36, 134.81, 128.86, 127.16, 127.03, 127.00, 126.97, 126.10, 125.28, 125.24, 120.83, 117.26, 112.78, 108.77, 108.54, 56.17, 17.48. HRMS (ESI): m/z calcd for C_25_H_19_Cl_2_FN_4_O_3_: 513.0891 [M+H] ^+^; found: 513.0890.

#### (*E*)-*N*-(2-((4-((3,4-Dichloro-2-fluorophenyl)amino)-7-methoxyquinazolin-6-yl) oxy)phenyl)but-2-enamide (4g)

A pale-yellow solid (158 mg, 46% yield); m.p: 236–238 °C; ^1^H NMR (400 MHz, DMSO-*d*_6_) *δ* 9.81 (s, 1H), 9.55 (s, 1H), 8.47 (s, 1H), 8.18 (d, *J* = 7.6 Hz, 1H), 8.14 (s, 1H), 7.53 (s, 1H), 7.42 (s, 1H), 6.99–7.08 (m, 3H), 6.81 (dd, *J* = 15.2, 6.9 Hz, 1H), 6.71 (d, *J* = 7.9 Hz, 1H), 6.48 (d, *J* = 15.2 Hz, 1H), 3.95 (s, 3H), 1.85 (dd, *J* = 6.9, 1.6 Hz, 3H). ^13^C NMR (101 MHz, DMSO-*d*_6_) *δ* 169.41, 163.96, 162.36, 156.36, 154.48, 148.29, 144.10, 144.07, 139.89, 132.67, 128.50, 128.34, 125.31, 125.27, 122.71, 122.63, 119.65, 119.47, 117.75, 115.73, 115.56, 108.51, 104.60, 56.26, 17.56. HRMS (ESI): m/z calcd for C_25_H_19_Cl_2_FN_4_O_3_: 513.0891 [M+H] ^+^; found: 513.0889.

#### (*E*)-*N*-(4-((4-((3,4-Dichloro-2-fluorophenyl)amino)-7-methoxyquinazolin-6-yl) oxy)-3-methoxyphenyl)but-2-enamide (4h)

A pale-yellow solid (193 mg, 53% yield); m.p: 205–207 °C; ^1^H NMR (400 MHz, DMSO-*d*_6_) *δ* 10.02 (s, 1H), 9.64 (s, 1H), 8.40 (s, 1H), 7.66 (d, *J* = 2.2 Hz, 1H), 7.61 (s, 1H), 7.51 (t, *J* = 11.1 Hz, 2 H), 7.34 (s, 6.08–6.12 (m, 1H), 3.99 (s, 3H), 3.76 (s, 3H), 1.86 (d, *J* = 1.6 Hz, 3H).^13^C NMR (101 MHz, DMSO - *d*_6_) *δ* 163.40, 157.03, 155.01, 153.50, 150.40, 148.10, 146.90, 139.83, 139.28, 136.81, 128.95, 127.34, 125.98, 125.26, 125.22, 120.36, 119.62, 119.43, 111.57, 108.56, 108.07, 107.42, 104.92, 56.09, 55.56, 17.54. HRMS (ESI): m/z calcd for C_26_H_21_Cl_2_FN_4_O_4_: 543.0996 [M+H] ^+^; found: 543.0994.

#### (*E*)-*N*-(2-((4-((3,4-Dichloro-2-fluorophenyl)amino)-7-methoxyquinazolin-6-yl) oxy)-5-methoxyphenyl)but-2-enamide (4i)

A pale-yellow solid (189 mg, 52% yield) ; m.p: 242–244 °C; ^1^H NMR (400 MHz, DMSO-*d*_6_) *δ* 9.74 (s, 1H), 9.47 (s, 1H), 8.44 (s, 1H), 7.94 (d, *J* = 7.0 Hz, 2H), 7.53 (d, *J* = 3.4 Hz, 2H), 7.39 (s, 1H), 6.82 (d, *J* = 6.9 Hz, 1H), 6.76 (s, 1H), 6.62 (dd, *J* = 9.0, 3.1 Hz, 1H), 6.43–6.46 (m, 1H), 3.97 (s, 3H), 3.72 (s, 3H), 1.84 (dd, *J* = 6.9, 1.6 Hz, 3H). ^13^C NMR (101 MHz, DMSO-*d*_6_) *δ* 164.07, 164.06, 157.20, 156.08, 154.86, 154.06, 148.82, 145.90, 140.20, 130.14, 128.98, 127.12, 127.10, 126.11, 125.34, 125.30, 119.50, 117.73, 111.82, 109.13, 108.68, 108.39, 56.21, 55.35, 17.56. HRMS (ESI): m/z calcd for C_26_H_21_Cl_2_FN_4_O_4_: 543.0996 [M+H] ^+^; found: 543.0995.

#### (*E*)-*N*-(2-((4-((3,4-Dichloro-2-fluorophenyl)amino)-7-methoxyquinazolin-6-yl) oxy)pyridin-4-yl)but-2-enamide (4j)

A pale-yellow solid (172 mg, 50% yield); m.p: 226–228 °C; ^1^H NMR (400 MHz, DMSO-*d*_6_) *δ* 10.13 (s, 1H), 9.86 (s, 1H), 8.53 (dd, *J* = 7.8, 1.6 Hz, 1H), 8.48 (s, 1H), 8.39 (s, 1H), 7.73 (dd, *J* = 4.9, 1.7 Hz, 1H), 7.55 (s, 2H), 7.38 (s, 1H), 7.09 (dd, *J* = 7.9, 4.9 Hz, 1H), 6.81–6.85 (m, 1H), 6.54 (dd, *J* = 15.2, 1.6 Hz, 1H), 3.85 (s, 3H), 1.86 (dd, *J* = 6.9, 1.6 Hz, 3H). ^13^C NMR (101 MHz, DMSO-*d*_6_) *δ* 164.66, 164.65, 157.55, 156.82, 153.73, 142.22, 140.89, 140.81, 132.54, 130.52, 128.99, 128.73, 127.12, 127.07, 125.77, 125.38, 122.64, 119.76, 119.57, 118.98, 116.64, 56.32, 17.73. HRMS (ESI): m/z calcd for C_24_H_18_Cl_2_FN_5_O_3_: 514.0843 [M+H] ^+^; found: 514.0842.

#### *N*-(4-((4-((3,4-Dichloro-2-fluorophenyl)amino)-7-methoxyquinazolin-6-yl)oxy) phenyl)methacrylamide (4k)

A pale-yellow solid (213 mg, 62% yield); m.p: 239–241 °C; ^1^H NMR (400 MHz, DMSO-*d*_6_) *δ* 9.79 (s, 1H), 9.77 (s, 1H), 8.47 (s, 1H), 8.07 (s, 1H), 7.68 (d, *J* = 9.1 Hz, 2H), 7.53 (s, 2H), 7.40 (s, 1H), 6.97 (s, 2H), 5.79 (s, 1H), 5.49 (s, 1H), 3.94 (s, 3H), 1.94 (s, 3H). ^13^C NMR (101 MHz, DMSO-*d*_6_) *δ* 166.59, 157.21, 156.19, 154.15, 153.08, 151.90, 148.99, 144.89, 140.40, 134.37, 128.88, 126.92, 125.29, 125.25, 121.86, 119.75, 119.67, 119.48, 117.05, 112.78, 108.79, 108.57, 56.17, 18.75. HRMS (ESI): m/z calcd for C_25_H_19_Cl_2_FN_4_O_3_: 513.0891 [M+H] ^+^; found: 513.0889.

#### *N*-(2-((4-((3,4-Dichloro-2-fluorophenyl)amino)-7-methoxyquinazolin-6-yl)oxy)phenyl)methacrylamide (4l)

A pale-yellow solid (188 mg, 55% yield); m.p: 240–242 °C; ^1^H NMR (400 MHz, DMSO-*d*_6_) *δ* 10.11 (s, 1H), 9.23 (s, 1H), 8.45 (s, 1H), 8.24 (s, 1H), 7.86 (dd, *J* = 6.0, 3.6 Hz, 1H), 7.53 (s, 2H), 7.38 (s, 1H), 7.09–7.13 (m, 2H), 6.91 (dd, *J* = 5.8, 3.7 Hz, 1H), 5.83 (s, 1H), 5.48–5.52 (m, 1H), 3.93 (s, 3H), 1.92–1.94 (m, 3H). ^13^C NMR (101 MHz, DMSO-*d*_6_) *δ* 166.40, 162.38, 156.05, 156.03, 149.30, 144.36, 144.33, 144.32, 139.90, 128.32, 125.66, 125.33, 125.28, 124.62, 123.19, 120.56, 119.64, 119.45, 116.87, 114.10, 100.69, 99.50, 56.30, 18.57. HRMS (ESI): m/z calcd for C_25_H_19_Cl_2_FN_4_O_3_: 513.0891 [M+H] ^+^; found: 513.0888.

#### *N*-(4-((4-((3,4-Dichloro-2-fluorophenyl)amino)-7-methoxyquinazolin-6-yl)oxy)-3-methoxyphenyl) methacrylamide (4m)

A pale-yellow solid (182 mg, 50% yield); m.p: 242–244 °C; ^1^H NMR (400 MHz, DMSO-*d*_6_) *δ* 9.84 (s, 1H), 9.65 (s, 1H), 8.41 (s, 1H), 7.67 (d, *J* = 2.2 Hz, 1H), 7.62 (s, 1H), 7.51 (t, *J* = 11.3 Hz, 2H), 7.35 (t, *J* = 5.5 Hz, 2H), 6.96 (d, *J* = 8.7 Hz, 1H), 5.79 (s, 1H), 5.51 (s, 1H), 3.99 (s, 3H), 3.76 (s, 3H), 1.95 (s, 3H). ^13^C NMR (101 MHz, DMSO-*d*_6_) *δ* 166.76, 157.04, 155.03, 153.52, 152.12, 150.21, 148.12, 146.83, 140.45, 139.52, 136.49, 128.94, 127.31, 125.27, 125.23, 120.05, 119.81, 119.42, 112.34, 108.57, 108.09, 107.56, 105.75, 56.09, 55.58, 18.74. HRMS (ESI): m/z calcd for C_26_H_21_Cl_2_FN_4_O_4_: 543.0996 [M+H] ^+^; found: 543.0994.

#### *N*-(2-((4-((3,4-Dichloro-2-fluorophenyl)amino)-7-methoxyquinazolin-6-yl)oxy)-5-methoxyphenyl)acrylamide (4n)

A pale-yellow solid (203 mg, 56% yield); m.p: 180–182 °C; ^1^H NMR (400 MHz, DMSO-*d*_6_) *δ* 9.80 (s, 1H), 9.06 (s, 1H), 8.44 (s, 1H), 7.98 (s, 1H), 7.61 (d, *J* = 3.0 Hz, 1H), 7.53 (s, 2H), 7.37 (s, 1H), 7.00 (d, *J* = 9.0 Hz, 1H), 6.73 (dd, *J* = 9.0, 3.1 Hz, 1H), 5.75 (s, 1H), 5.48 (s, 1H), 3.96 (s, 3H), 3.74 (s, 3H), 1.90 (s, 3H). ^13^C NMR (101 MHz, DMSO-*d*_6_) *δ* 166.31, 157.24, 155.64, 155.27, 154.04, 145.71, 141.66, 139.85, 129.75, 126.91, 125.32, 125.28, 120.61, 119.66, 119.47, 119.04, 111.45, 110.18, 109.43, 108.67, 108.35, 99.48, 56.27, 55.43, 18.39. HRMS (ESI): m/z calcd for C_26_H_21_Cl_2_FN_4_O_4_: 543.0996 [M+H] ^+^; found: 543.0993.

#### *N*-(2-((4-((3,4-Dichloro-2-fluorophenyl)amino)-7-methoxyquinazolin-6-yl)oxy)pyridin-4-yl)methacrylamide (4o)

A pale-yellow solid (203 mg, 59% yield); m.p: 237–239 °C; ^1^H NMR (400 MHz, DMSO-*d*_6_) *δ* 9.85 (s, 1H), 9.46 (s, 1H), 8.50 (s, 1H), 8.27 (s, 1H), 8.21 (d, *J* = 9.2 Hz, 1H), 7.84 (d, *J* = 6.5 Hz, 1H), 7.55 (s, 2H), 7.39 (s, 1H), 7.14 (dd, *J* = 7.7, 4.9 Hz, 1H), 5.94 (s, 1H), 5.58 (s, 1H), 3.87 (s, 3H), 2.00 (s, 3H). ^13^C NMR (101 MHz, DMSO-*d*_6_) *δ* 167.02, 157.45, 156.74, 155.22, 154.52, 149.83, 142.20, 139.55, 132.96, 128.90, 127.01, 126.87, 125.35, 125.31, 121.99, 121.15, 119.71, 119.52, 118.96, 116.31, 108.68, 108.24, 56.23, 18.62. HRMS (ESI): m/z calcd for C_24_H_18_Cl_2_FN_5_O_3_: 514.0843 [M+H] ^+^; found: 514.0842.

#### *N*-(4-((4-((3,4-Dichloro-2-fluorophenyl)amino)-7-methoxyquinazolin-6-yl)oxy)phenyl)-3-methylbut-2-enamide (4p)

A pale-yellow solid (190 mg, 54% yield); m.p: 211–213 °C; ^1^H NMR (400 MHz, DMSO-*d*_6_) *δ* 10.03 (s, 1H), 9.92 (s, 1H), 8.45 (s, 1H), 8.12 (s, 1H), 7.66 (d, *J* = 9.0 Hz, 2H), 7.53 (d, *J* = 3.9 Hz, 2H), 7.38 (s, 1H), 6.95 (d, *J* = 9.0 Hz, 2H), 5.91 (s, 1H), 3.93 (s, 3H), 2.13 (s, 3H), 1.83 (s, 3H). ^13^C NMR (101 MHz, DMSO-*d*_6_) *δ* 164.48, 157.28, 156.20, 154.49, 154.15, 152.59, 151.99, 150.78, 148.99, 145.00, 135.09, 128.87, 127.21, 127.08, 126.99, 125.29, 125.25, 120.67, 119.29, 117.22, 112.83, 108.82, 108.52, 56.18, 27.02, 19.47. HRMS (ESI): m/z calcd for C_26_H_21_Cl_2_FN_4_O_3_: 527.1047 [M+H] ^+^; found: 527.1046.

#### *N*-(2-((4-((3,4-Dichloro-2-fluorophenyl)amino)-7-methoxyquinazolin-6-yl)oxy)phenyl)-3-methylbut-2-enamide (4q)

A pale-yellow solid (201 mg, 57% yield); m.p: 214–216 °C; ^1^H NMR (400 MHz, DMSO-*d*_6_) *δ* 9.81 (s, 1H), 9.35 (s, 1H), 8.48 (s, 1H), 8.14 (s, 1H), 7.54 (d, *J* = 3.5 Hz, 2H), 7.42 (s, 1H), 6.97–7.08 (m, 3H), 6.72 (dd, *J* = 8.0, 1.5 Hz, 1H), 6.19 (s, 1H), 3.95 (s, 3H), 2.16 (d, *J* = 1.0 Hz, 3H), 1.84–1.85 (m, 3H). ^13^C NMR (101 MHz, DMSO-*d*_6_) *δ* 164.93, 157.31, 156.40, 154.35, 151.46, 149.31, 148.06, 144.29, 128.93, 128.74, 126.93, 125.32, 125.27, 124.14, 122.61, 119.71, 119.52, 119.30, 115.52, 114.05, 108.82, 108.61, 56.23, 27.02, 19.54. HRMS (ESI): m/z calcd for C_26_H_21_Cl_2_FN_4_O_3_: 527.1047 [M+H] ^+^; found: 527.1043.

#### *N*-(4-((4-((3,4-Dichloro-2-fluorophenyl)amino)-7-methoxyquinazolin-6-yl)oxy)-3-methoxyphenyl)-3-methylbut-2-enamide (4r)

A pale-yellow solid (257 mg, 69% yield); m.p: 162–164 °C; ^1^H NMR (400 MHz, DMSO-*d*_6_) *δ* 9.95 (s, 1H), 9.68 (s, 1H), 8.40 (s, 1H), 7.69 (s, 1H), 7.60 (s, 1H), 7.51 (s, 2H), 7.34 (s, 1H), 7.19 (d, *J* = 7.4 Hz, 1H), 6.95 (d, *J* = 8.7 Hz, 1H), 5.88 (s, 1H), 3.99 (s, 3H), 3.75 (s, 3H), 2.15 (s, 3H), 1.86 (s, 3H). ^13^C NMR (101 MHz, DMSO-*d*_6_) *δ* 164.56, 157.03, 154.99, 153.29, 151.26, 150.38, 146.99, 138.95, 137.16, 128.91, 127.29, 125.25, 125.23, 125.21, 120.35, 119.61, 119.42, 119.16, 111.35, 108.60, 107.38, 104.71, 56.08, 55.54, 27.05, 19.49. HRMS (ESI): m/z calcd for C_27_H_23_Cl_2_FN_4_O_4_: 557.1153 [M+H] ^+^; found: 557.1152.

#### *N*-(2-((4-((3,4-Dichloro-2-fluorophenyl)amino)-7-methoxyquinazolin-6-yl)oxy)-5-methoxyphenyl)-3-methylbut-2-enamide (4s)

A pale-yellow solid (227 mg, 61% yield); m.p: 207–209 °C; ^1^H NMR (400 MHz, DMSO-*d*_6_) *δ* 9.75 (s, 1H), 9.25 (s, 1H), 8.43 (s, 1H), 7.92 (s, 2H), 7.52 (s, 2H), 7.37 (s, 1H), 6.76 (s, 1H), 6.60 (dd, *J* = 8.9, 3.0 Hz, 1H), 6.15 (s, 1H), 3.96 (s, 3H), 3.72 (s, 3H), 2.13 (s, 3H), 1.83 (s, 3H). ^13^C NMR (101 MHz, DMSO-*d*_6_) *δ* 165.03, 164.06, 156.12, 154.96, 154.91, 151.87, 146.10, 146.00, 140.22, 130.47, 130.20, 127.06, 126.11, 125.36, 125.30, 119.71, 119.52, 119.28, 117.79, 111.87, 109.17, 108.05, 99.49, 56.23, 55.36, 19.59, 17.56. HRMS (ESI): m/z calcd for C_27_H_23_Cl_2_FN_4_O_4_: 557.1153 [M+H] ^+^; found: 557.1152.

#### *N*-(2-((4-((3,4-Dichloro-2-fluorophenyl)amino)-7-methoxyquinazolin-6-yl)oxy)pyridin-4-yl)-3-methylbut-2-enamide (4t)

A pale-yellow solid (197 mg, 56% yield); m.p: 246–248 °C; ^1^H NMR (400 MHz, DMSO-*d*_6_) *δ* 9.79 (s, 1H), 9.65 (s, 1H), 8.53 (dd, *J* = 7.9, 1.6 Hz, 1H), 8.50 (s, 1H), 8.28 (s, 1H), 7.72 (dd, *J* = 4.9, 1.7 Hz, 1H), 7.56 (s, 2H), 7.40 (s, 1H), 7.09 (dd, *J* = 7.9, 4.9 Hz, 1H), 6.27 (s, 1H), 3.88 (s, 3H), 2.19 (d, *J* = 1.1 Hz, 3H), 1.87 (d, *J* = 1.1 Hz, 3H). ^13^C NMR (101 MHz, DMSO-*d*_6_) *δ* 165.44, 157.39, 156.79, 154.52, 153.50, 152.51, 151.85, 149.85, 142.24, 140.36, 128.91, 126.90, 125.34, 125.30, 122.81, 119.71, 119.53, 118.92, 118.83, 116.23, 108.67, 108.26, 56.21, 27.13, 19.65. HRMS (ESI): m/z calcd for C_25_H_20_Cl_2_FN_5_O_3_: 528.1000 [M+H] ^+^; found: 528.1001.

## Design, synthesis, and in vitro antitumor activity of 6-aryloxyl substituted quinazoline derivatives

[Table t2-turkjchem-46-3-849]

**Table t2-turkjchem-46-3-849:** Table of contents

1.	The general synthetic procedure for intermediates **2a–2e** and **3a–3e**.	S2–S4
2.	^1^H and ^13^C NMR spectra of compounds **4a–4t**	S5–S29

### 1. Experimental procedures and physical data of compounds

#### 1.1 General procedure for the preparation of intermediates 2a–2e

##### *N*-(3,4-Dichloro-2-fluorophenyl)-7-methoxy-6-(4-nitrophenoxy)quinazolin-4-amine (2a)

To a solution of compound **1** (5.00 g, 14.1 mmol), potassium carbonate (K_2_CO_3_) (5.85 g, 42.3 mmol) in *N*,*N*-dimethylformamide (DMF) (50.0 mL), was dropwised 1-fluoro-4-nitrobenzene (1.50 mL, 14.1 mmol) slowly at 0 °C. After addition, the reaction mixture was allowed to stir for 6 h at 50 °C and TLC indicated complete consumption of the starting material. The reaction mixture was partitioned between water and ethyl acetate, the organic layers were combined, dried, filtered, and concentrated. The residue was purified by chromatography to afford the compound **2a** as yellow powder (5.8 g, 86.6%).^1^H NMR (400 MHz, DMSO-*d*_6_) *δ* 9.78 (s, 1H), 8.48 (s, 1H), 8.30 (s, 1H), 8.21 (d, *J* = 9.3 Hz, 2H), 7.52 (s, 2H), 7.44 (s, 1H), 7.13 (d, *J* = 9.3 Hz, 2H), 3.87 (s, 3H).

##### *N*-(3,4-Dichloro-2-fluorophenyl)-7-methoxy-6-(2-nitrophenoxy)quinazolin-4-amine (2b)

The synthetic method of compound **2b** was the same to compound **2a**. Compound **2b** as yellow solid (5.5 g, 82.1%). ^1^H NMR (400 MHz, DMSO-*d*_6_) *δ* 9.80 (s, 1H), 8.51 (s, 1H), 8.25 (s, 1H), 8.08 (dd, *J* = 8.1, 1.6 Hz, 1H), 7.63 (m, 1H), 7.56 (d, *J* = 4.5 Hz, 2H), 7.45 (s, 1H), 7.32–7.35 (m, 1H), 7.05 (dd, *J* = 8.4, 1.1 Hz, 1H), 3.93 (s, 3H).

##### *N*-(3,4-Dichloro-2-fluorophenyl)-7-methoxy-6-(2-methoxy-4-nitrophenoxy)-quinazolin-4-amine (2c)

The synthetic method of compound **2c** was the same to compound **2a**. Compound **2c** as yellow solid (5.7 g, 85.1%). ^1^H NMR (400 MHz, DMSO-*d_6_*) *δ* 9.69 (s, 1H), 8.46 (s, 1H), 8.11 (s, 1H), 7.91 (d, *J* = 2.6 Hz, 1H), 7.80 (dd, *J* = 8.9, 2.7 Hz, 1H), 7.52 - 7.51 (m, 2H), 7.42 (s, 1H), 6.90 (d, *J* = 8.9 Hz, 1H), 3.95 (s, 3H), 3.90 (s, 3H).

##### *N-*(3,4-Dichloro-2-fluorophenyl)-7-methoxy-6-(4-methoxy-2-nitrophenoxy)-quinazolin-4-amine (2d)

The synthetic method of compound **2d** was the same to compound **2a**. Compound **2d** as yellow solid (5.6 g, 83.6%). ^1^H NMR (400 MHz, DMSO-*d*_6_) *δ* 9.72 (s, 1H), 8.46 (s, 1H), 8.00 (s, 1H), 7.66 (d, *J* = 3.1 Hz, 1H), 7.54 (s, 2H), 7.41 (s, 1H), 7.29 (dd, *J* = 9.2, 3.1 Hz, 1H), 7.16 (d, *J* = 9.2 Hz, 1H), 3.95 (s, 3H), 3.85 (s, 3H).

##### *N*-(3,4-Dichloro-2-fluorophenyl)-7-methoxy-6-((4-nitropyridin-2-yl)oxy)-quinazolin-4-amine (2e)

The synthetic method of compound **2e** was the same to compound **2a**. Compound **2e** as yellow solid (5.5 g, 82.1%). ^1^H NMR (400 MHz, DMSO-*d*_6_) *δ* 9.82 (s, 1H), 8.63 (dd, *J* = 8.0, 1.6 Hz, 1H), 8.52 (s, 1H), 8.41 (dd, *J* = 4.8, 1.7 Hz, 1H), 8.37 (s, 1H), 7.58 (d, *J* = 8.2 Hz, 2H), 7.43 (d, *J* = 4.8 Hz, 1H), 7.41 (d, *J* = 4.8 Hz, 1H), 3.86 (s, 3H).

#### 1.2 General procedure for the preparation of intermediates 3a–3e

##### 6-(4-aminophenoxy)-*N*-(3,4-dichloro-2-fluorophenyl)-7-methoxyquinazolin-4-amine (3a)

A suspension of **2a** (1.00 g, 2.10 mmol), Fe (0.35 g, 6.30 mmol), and NH_4_Cl (0.58 g, 10.5 mmol) in CH_3_OH/H_2_O (10 mL/10 mL) was stirred at 80 °C for 4 h. The mixture was cooled to room temperature, quenched with aqueous Na_2_CO_3_ solution, and filtered through celite. The aqueous layer was separated and extracted with ethyl acetate. The combined organic layers were washed with water and brine, dried over Na_2_SO_4_, and concentrated. The residue was purified by chromatography to afford the compound **3a** as yellow powder (0.7 g, 75.3 %). ^1^H NMR (400 MHz, DMSO-*d*_6_) *δ* 9.67 (s, 1H), 8.38 (s, 1H), 7.76 (s, 1H), 7.49 (s, 2H), 7.30 (s, 1H), 6.77 (d, *J* = 8.9 Hz, 2H), 6.59 (d, *J* = 9.1 Hz, 2H), 4.92 (s, 2H), 3.94 (s, 3H).

##### 6-(2-aminophenoxy)-*N*-(3,4-dichloro-2-fluorophenyl)-7-methoxyquinazolin-4-amine (3b)

The synthetic method of compound **3b** was the same to compound **3a**. Compound **3b** as yellow solid (0.75 g, 80.6%). ^1^H NMR (400 MHz, DMSO-*d*_6_) *δ* 9.74 (s, 1H), 8.44 (s, 1H), 7.89 (s, 1H), 7.52 (s, 2H), 7.38 (s, 1H), 6.87 (m, 1H), 6.81 (dd, *J* = 7.9, 1.7 Hz, 1H), 6.65 (d, *J* = 6.9 Hz, 1H), 6.53–6.49 (m, 1H), 4.97 (s, 2H), 3.97 (s, 3H).

##### 6-(4-amino-2-methoxyphenoxy)-*N*-(3,4-dichloro-2-fluorophenyl)-7-methoxyquinazolin-4-ami ne (3c)

The synthetic method of compound **3c** was the same to compound **3a**. Compound **3c** as yellow solid (0.8 g, 85.1%). ^1^H NMR (400 MHz, DMSO-*d*_6_) *δ* 9.63 (s, 1H), 8.36 (s, 1H), 7.50 (s, 2H), 7.42 (s, 1H), 7.28 (s, 1H), 6.77 (d, *J* = 8.5 Hz, 1H), 6.41 (d, *J* = 2.4 Hz, 1H), 6.18 (dd, *J* = 8.5, 2.5 Hz, 1H), 5.07 (s, 2H), 3.99 (s, 3H), 3.66 (s, 3H).

##### 6-(2-amino-4-methoxyphenoxy)-*N*-(3,4-dichloro-2-fluorophenyl)-7-methoxyquinazolin-4-ami ne (3d)

The synthetic method of compound **3d** was the same to compound **3a**. Compound **3d** as yellow solid (0.66 g, 71.0%). ^1^H-NMR (400 MHz, DMSO-*d*_6_) *δ* 9.70 (s, 1H), 8.40 (s, 1H), 7.68 (s, 1H), 7.53 (d, *J* = 10.1 Hz, 1H), 7.50–7.45 (m, 1H), 7.34 (s, 1H), 6.72 (d, *J* = 8.7 Hz, 1H), 6.41 (d, *J* = 2.9 Hz, 1H), 6.13 (dd, *J* = 8.8, 3.0 Hz, 1H), 4.96 (s, 2H), 3.99 (s, 3H), 3.67 (s, 3H).

##### 6-((4-aminopyridin-2-yl)oxy)-*N*-(3,4-dichloro-2-fluorophenyl)-7-methoxyquinazolin-4-amine (3e)

To a solution of compound **2e** (2.0 g, 4.21 mmol) in 40 mL of methanol was added 1.0 g of 10% Pd on carbon catalyst. The reaction mixture was allowed to stir for 2 h at 40 °C under H_2_ atmosphere. The solution was filtered through a pad of celite, dried and concentrated to give compound **3e** as yellow powder (0.82 g, 88.2 %). ^1^H NMR (400 MHz, DMSO-*d*_6_) δ 9.70 (s, 1H), 8.39 (s, 1H), 8.35 (d, *J* = 9.4 Hz, 1H), 7.51 (t, *J* = 7.8 Hz, 1H), 7.29–7.28 (m, 1H), 7.28 (s, 1H), 7.26–7.25 (m, 1H), 7.23 (d, *J* = 3.2 Hz, 1H), 7.21 (s, 1H), 7.20 (s, 2H), 3.88 (s, 3H).

### 2. ^1^H and ^13^C NMR spectra of compounds 2, 3, and 4

^1^HNMR spectrum of 2a

^1^HNMR spectrum of 2b

^1^HNMR spectrum of 2c

^1^HNMR spectrum of 2d

^1^HNMR spectrum of 2e

^1^HNMR spectrum of 3a

^1^HNMR spectrum of 3b

^1^HNMR spectrum of 3c

^1^HNMR spectrum of 3d

^1^HNMR spectrum of 3e

^1^H NMR spectrum of 4a

^13^C NMR spectrum of 4a

^1^H NMR spectrum of 4b

^13^C NMR spectrum of 4b

^1^H NMR spectrum of 4c

^13^C NMR spectrum of 4c

^1^H NMR spectrum of 4d

^13^C NMR spectrum of 4d

^1^H NMR spectrum of 4e

^13^C NMR spectrum of 4e

^1^H NMR spectrum of 4f

^13^C NMR spectrum of 4f

^1^H NMR spectrum of 4g

^13^C NMR spectrum of 4g

^1^H NMR spectrum of 4h

^13^C NMR spectrum of 4h

^1^H NMR spectrum of 4i

^13^C NMR spectrum of 4i

^1^H NMR spectrum of 4j

^13^C NMR spectrum of 4j

^1^H NMR spectrum of 4k

^13^C NMR spectrum of 4k

^1^H NMR spectrum of 4l

^13^C NMR spectrum of 4l

^1^H NMR spectrum of 4m

^13^C NMR spectrum of 4m

^1^H NMR spectrum of 4n

^13^C NMR spectrum of 4n

^1^H NMR spectrum of 4o

^13^C NMR spectrum of 4o

^1^H NMR spectrum of 4p

^13^C NMR spectrum of 4p

^1^H NMR spectrum of 4q

^13^C NMR spectrum of 4q

^1^H NMR spectrum of 4r

^13^C NMR spectrum of 4r

^1^H NMR spectrum of 4s

^13^C NMR spectrum of 4s

^1^H NMR spectrum of 4t

^13^C NMR spectrum of 4t

## Figures and Tables

**Scheme.**
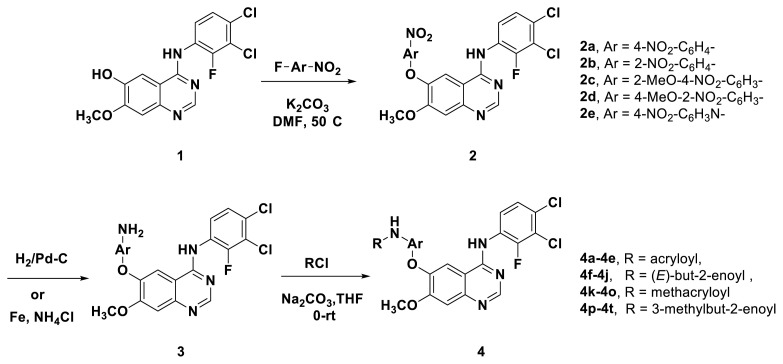
Synthetic route of target compounds **4a**–**4t**.

**Table.** The structure and antiproliferative activities of compounds **4a**–**4t**.

Compound	R	IC_50_^a^		
		N87(nM)	H1975(nM)	A549(μM)
**4a**	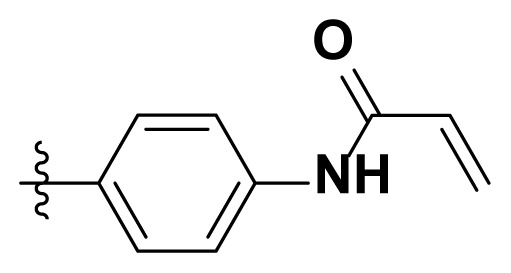	4.7 ± 1.5	12.3 ± 2.4	36.3 ± 1.5
**4b**	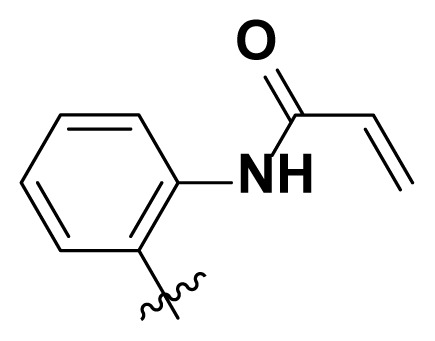	46.4 ± 6.7	88.5 ± 23.1	40.4 ± 1.7
**4c**	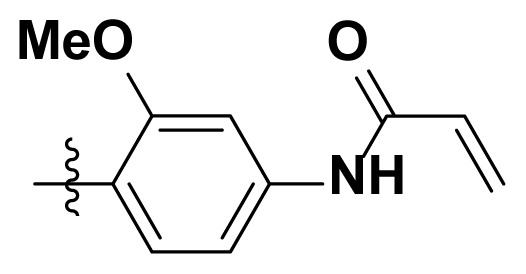	12.3 ± 2.1	35.4 ± 11.1	38.6 ± 2.8
**4d**	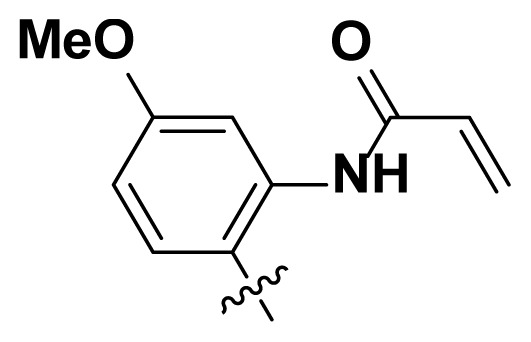	9.6 ± 1.1	21.3 ± 9.2	30.2 ± 1.6
**4e**	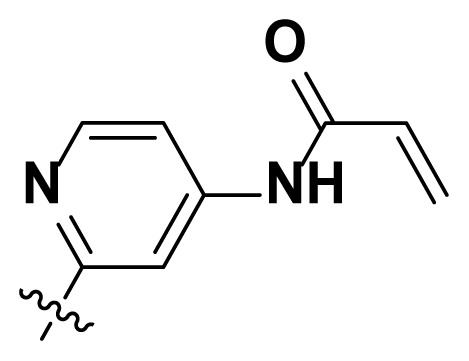	121 ± 20.5	434 ± 32.5	58.3 ± 2.8
**4f**	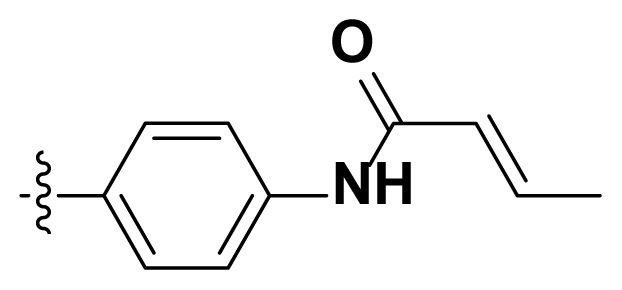	43.4 ± 5.1	63.6 ± 15.3	43.7 ± 1.4
**4g**	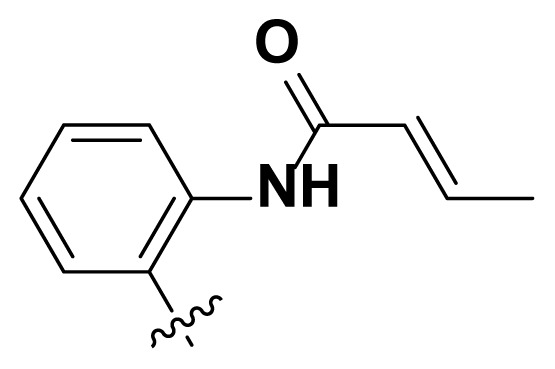	15.7 ± 6.1	8.3 ± 2.1	28.9 ± 1.6
**4h**	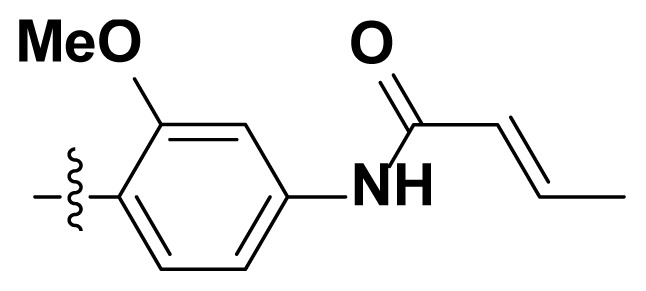	46.7 ± 11.2	98.5 ± 18.5	45.4 ± 2.0
**4i**	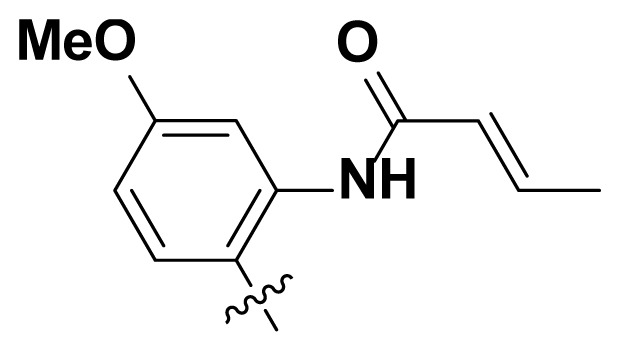	25.2 ± 1.7	53.1 ± 14.4	40.7 ± 2.5
**4j**	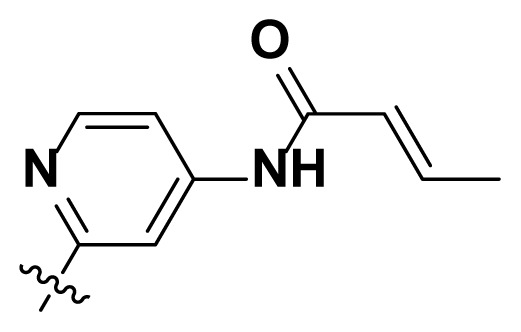	780 ± 45.8	1250 ± 78.0	73.3 ± 1.6
**4k**	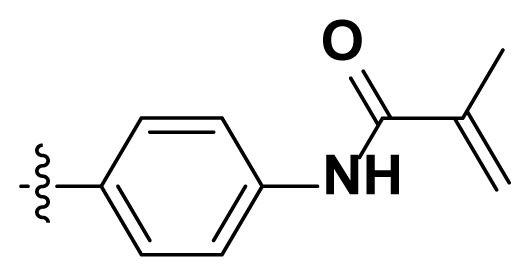	5.3 ± 1.1	9.5 ± 3.5	35.1 ± 1.7
**4l**	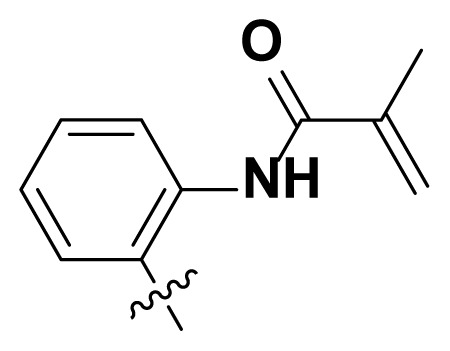	37.5 ± 14.7	24.6 ± 5.8	38.2 ± 3.3
**4m**	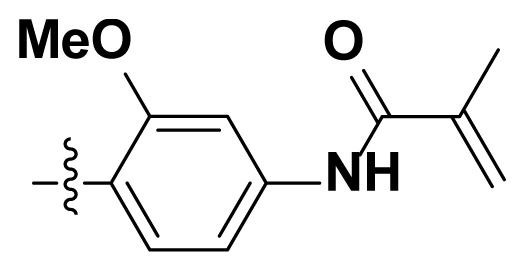	6.3 ± 1.7	7.5 ± 4.4	29.9 ± 2.5
**4n**	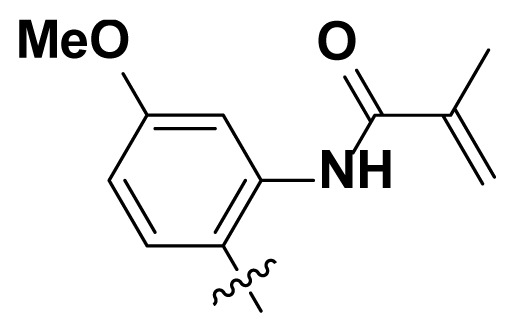	27.3 ± 3.2	41.5 ± 17.3	35.8 ± 1.5
**4o**	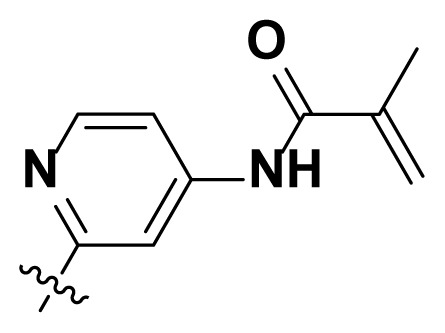	272 ± 27.9	327 ± 25.8	57.4 ± 2.4
**4p**	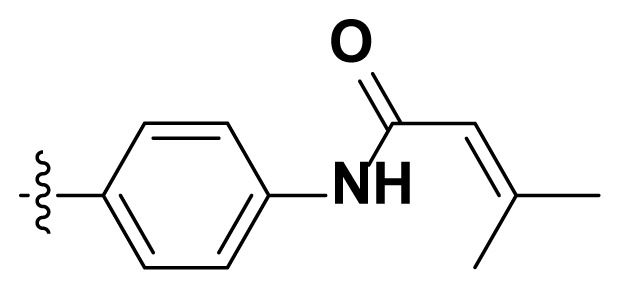	16.9 ± 3.7	77.5 ± 12.4	36.3 ± 3.0
**4q**	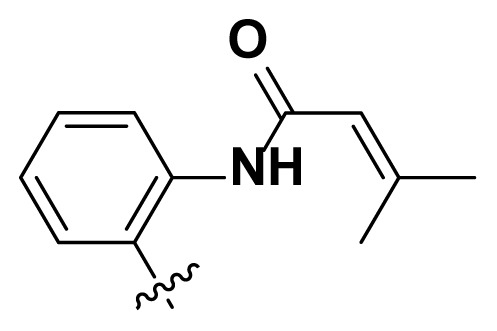	74.3 ± 10.6	14.6 ± 5.8	42.3 ± 1.6
**4r**	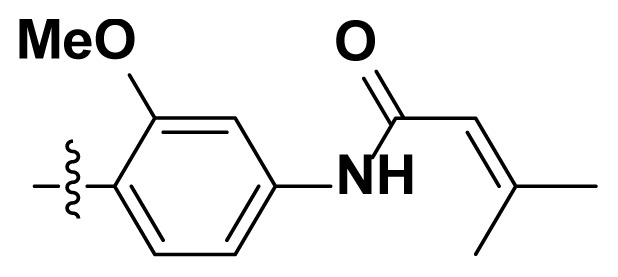	83.6 ± 24.3	125.3 ± 27.4	50.6 ± 2.5
**4s**	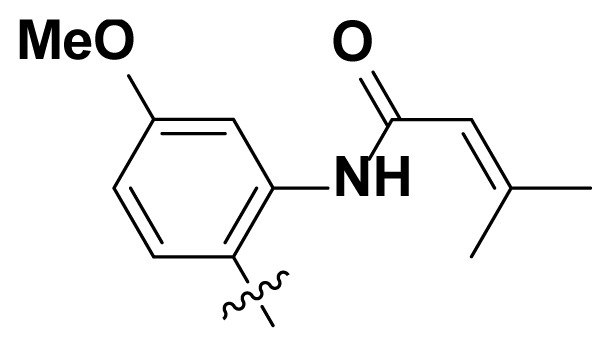	97.2 ± 7.9	173.3 ± 45.7	55.3 ± 1.4
**4t**	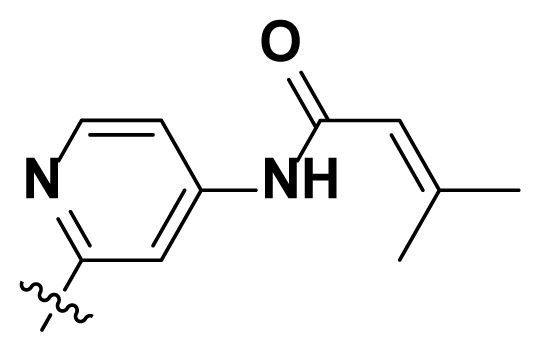	567 ± 39.5	870 ± 24.2	68.4 ± 1.3
**Poziotinib**	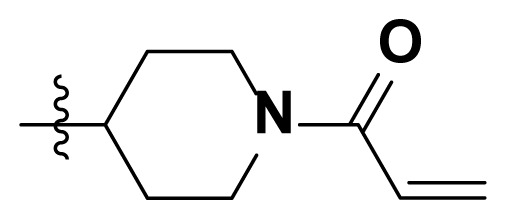	1.1 ± 0.03	6.9 ± 0.4	23.5 ± 2.1

a The IC_50_ values represent an average of three experiments ± SD.
