# Supplementary figures and images for: Design, synthesis, and in vitro antitumor activity of 6-aryloxyl substituted quinazoline derivatives
Source: Turk J Chem. 2022 Feb 23;46(3):849–58. doi: 10.55730/1300-0527.3373 (PMC10503992; doi:10.55730/1300-0527.3373)

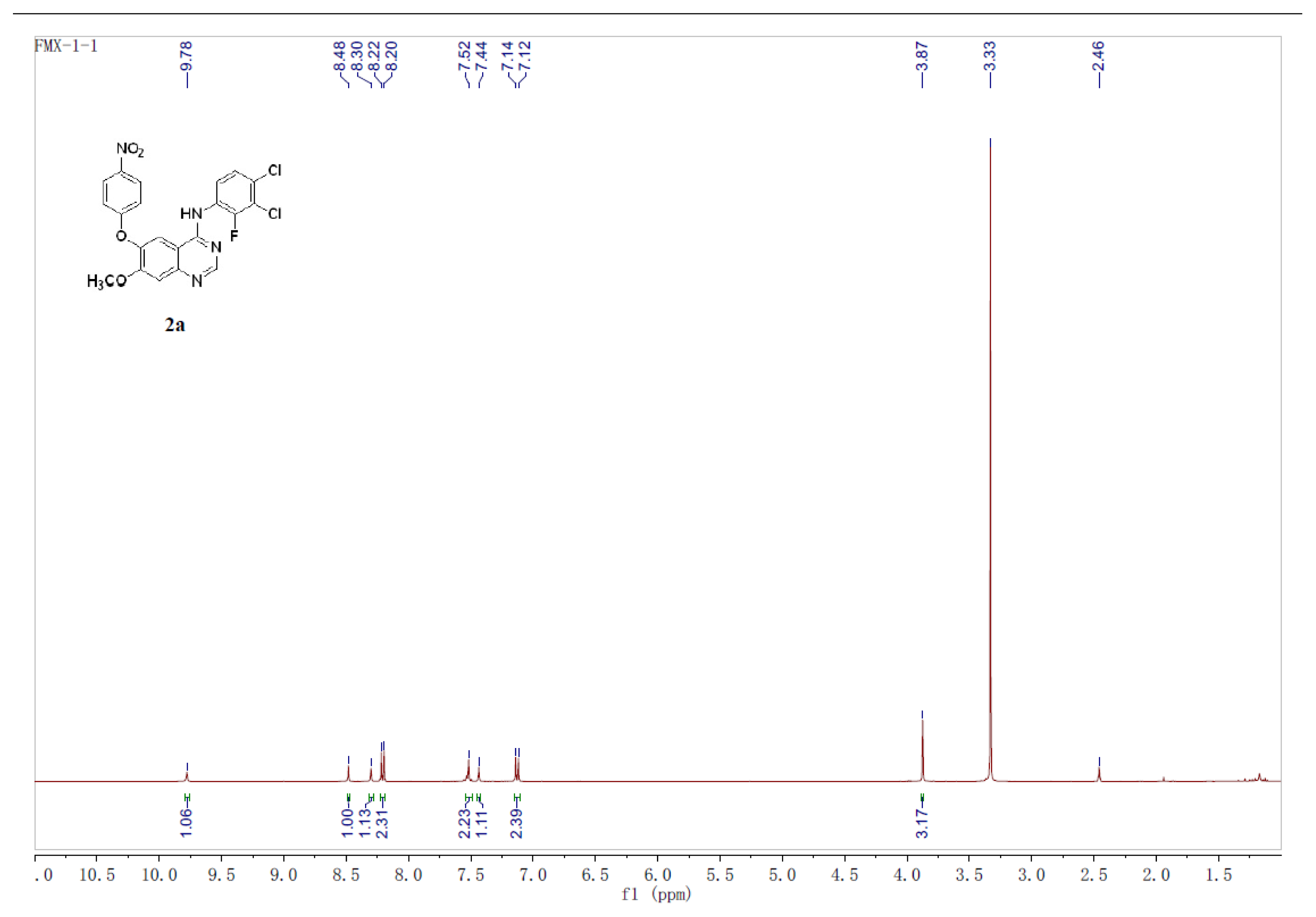

Supplement: Supplementary file 1 — 1HNMR spectrum of 2a [file turkjchem-46-3-849s1.tif]

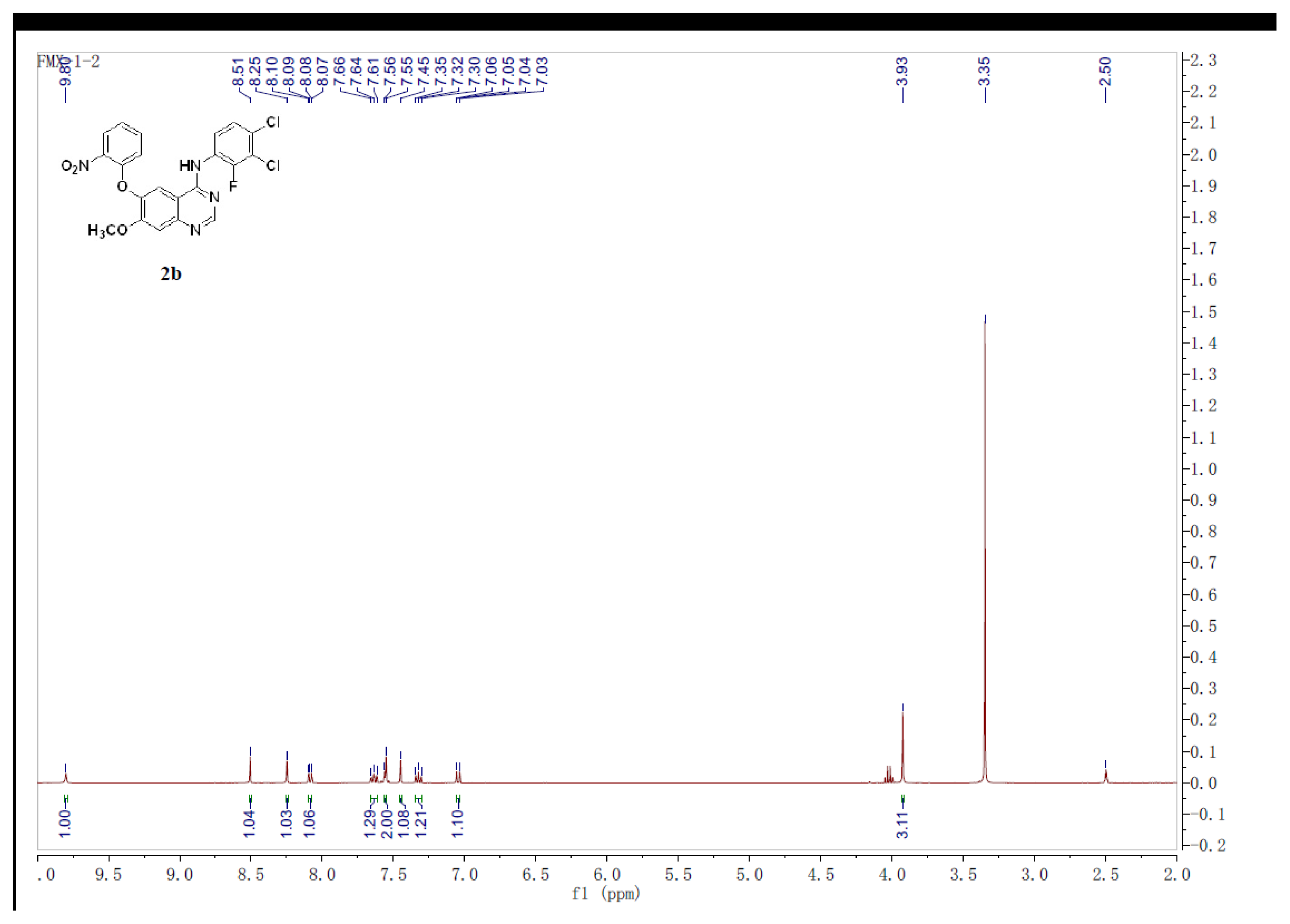

Supplement: Supplementary file 2 — 1HNMR spectrum of 2b [file turkjchem-46-3-849s2.tif]

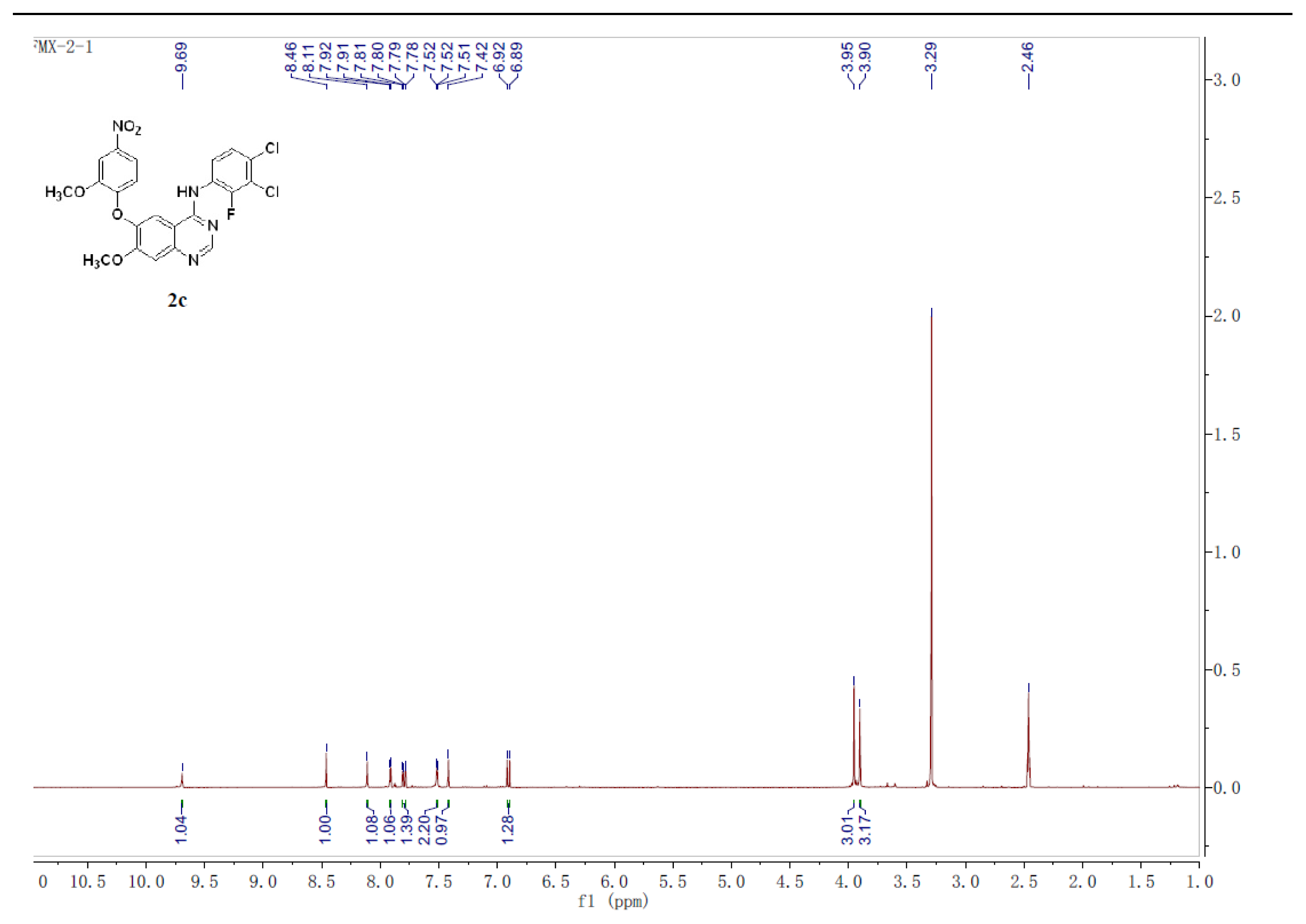

Supplement: Supplementary file 3 — 1HNMR spectrum of 2c [file turkjchem-46-3-849s3.tif]

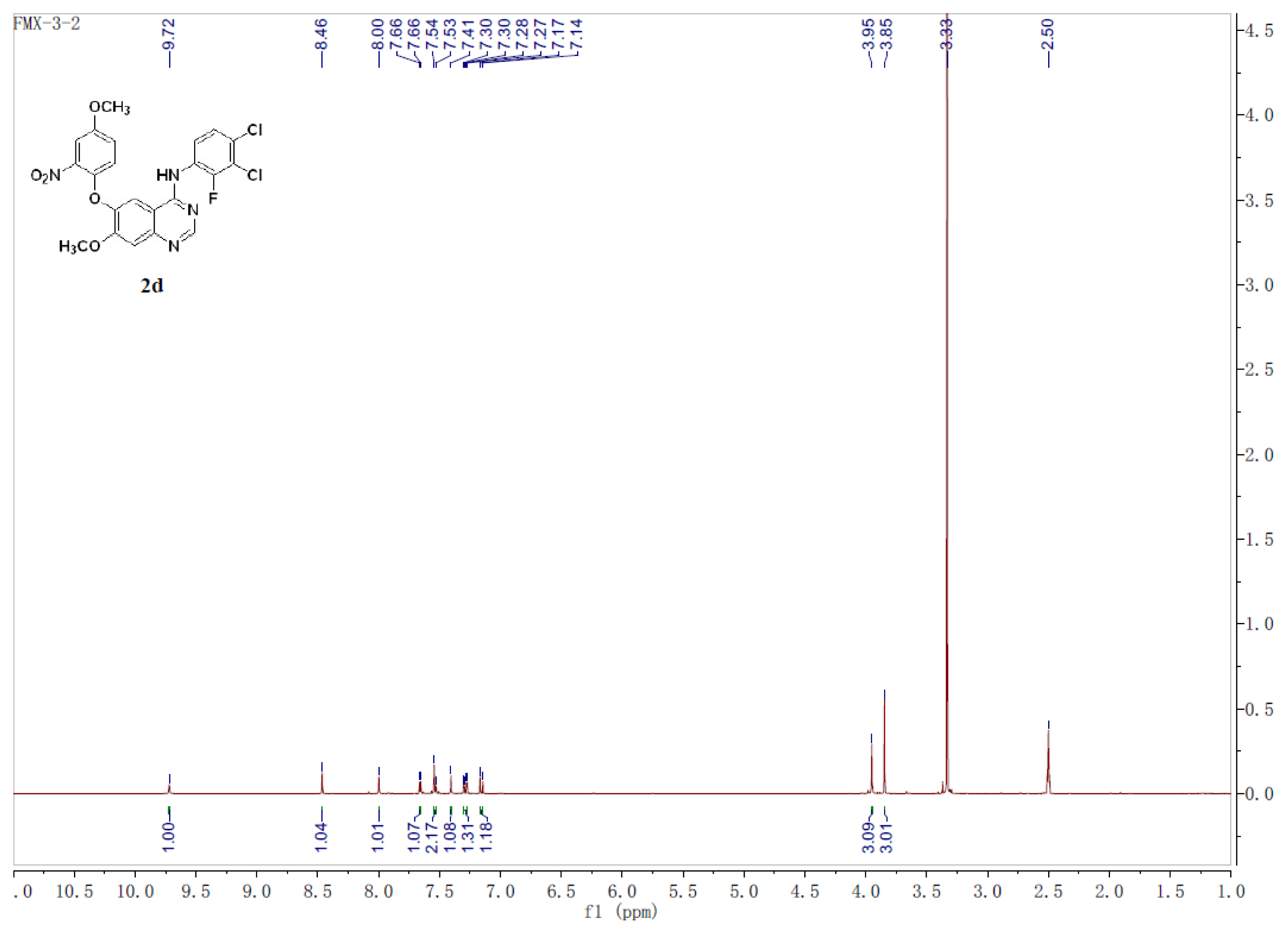

Supplement: Supplementary file 4 — 1HNMR spectrum of 2d [file turkjchem-46-3-849s4.tif]

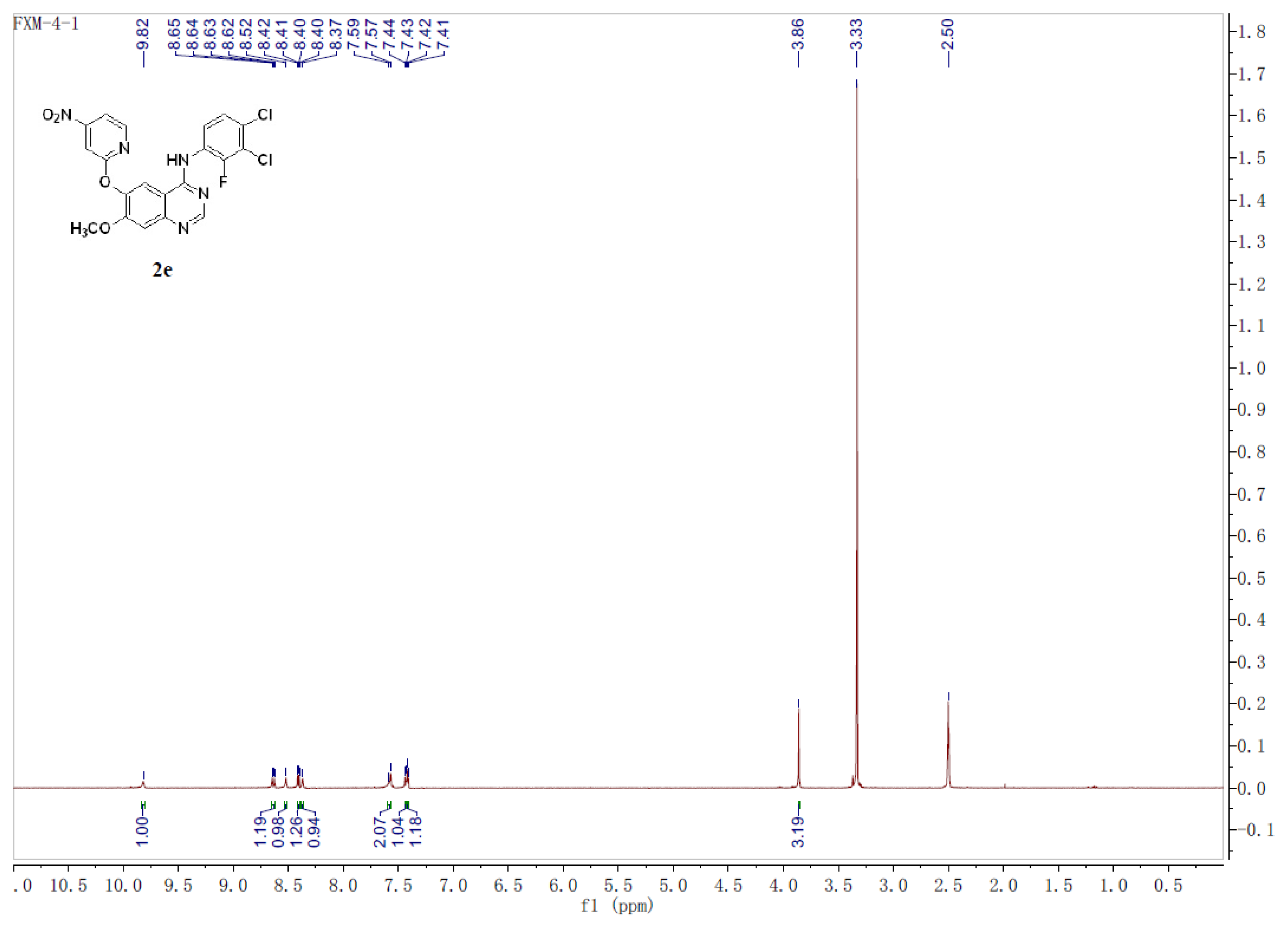

Supplement: Supplementary file 5 — 1HNMR spectrum of 2e [file turkjchem-46-3-849s5.tif]

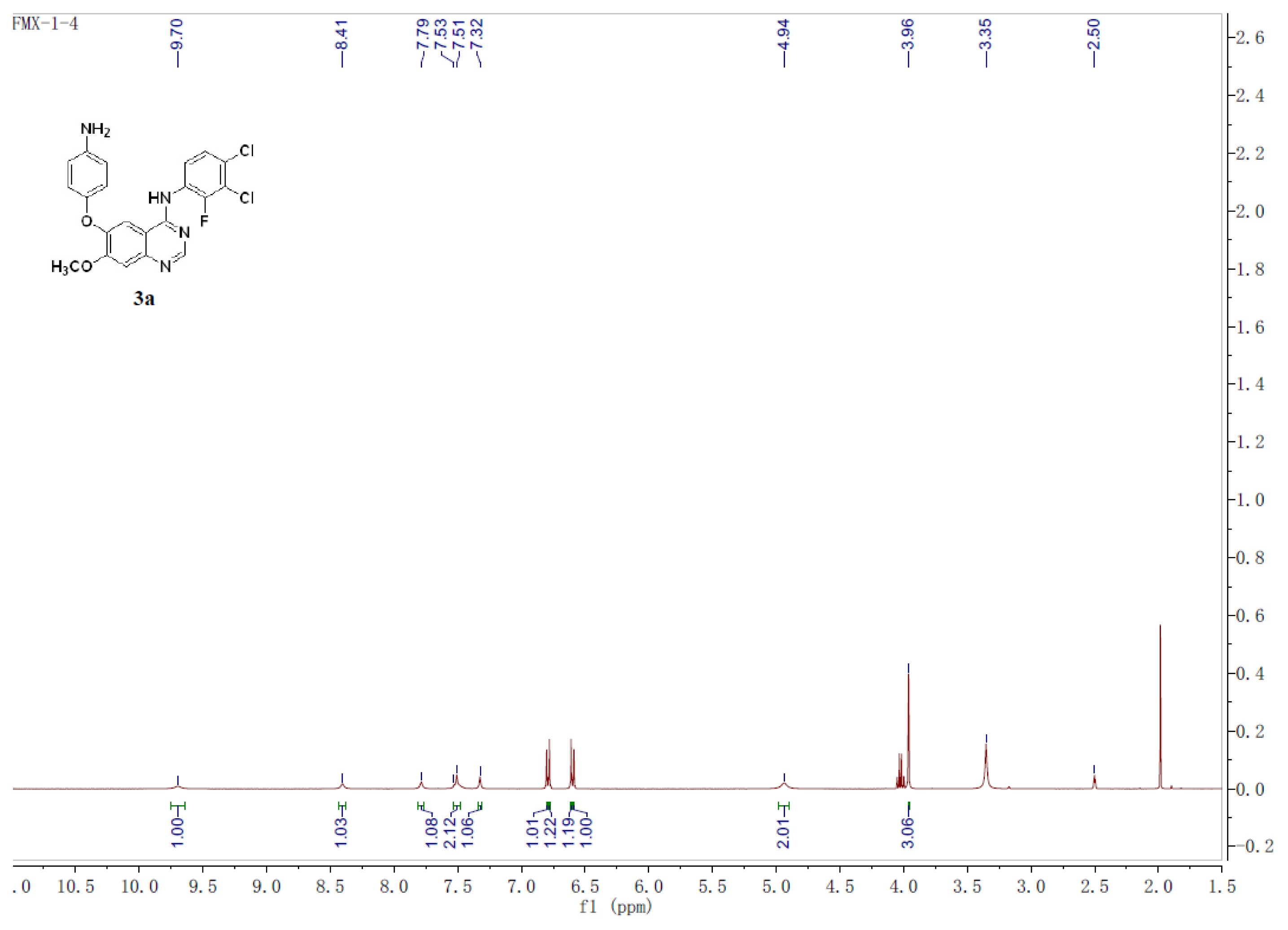

Supplement: Supplementary file 6 — 1HNMR spectrum of 3a [file turkjchem-46-3-849s6.tif]

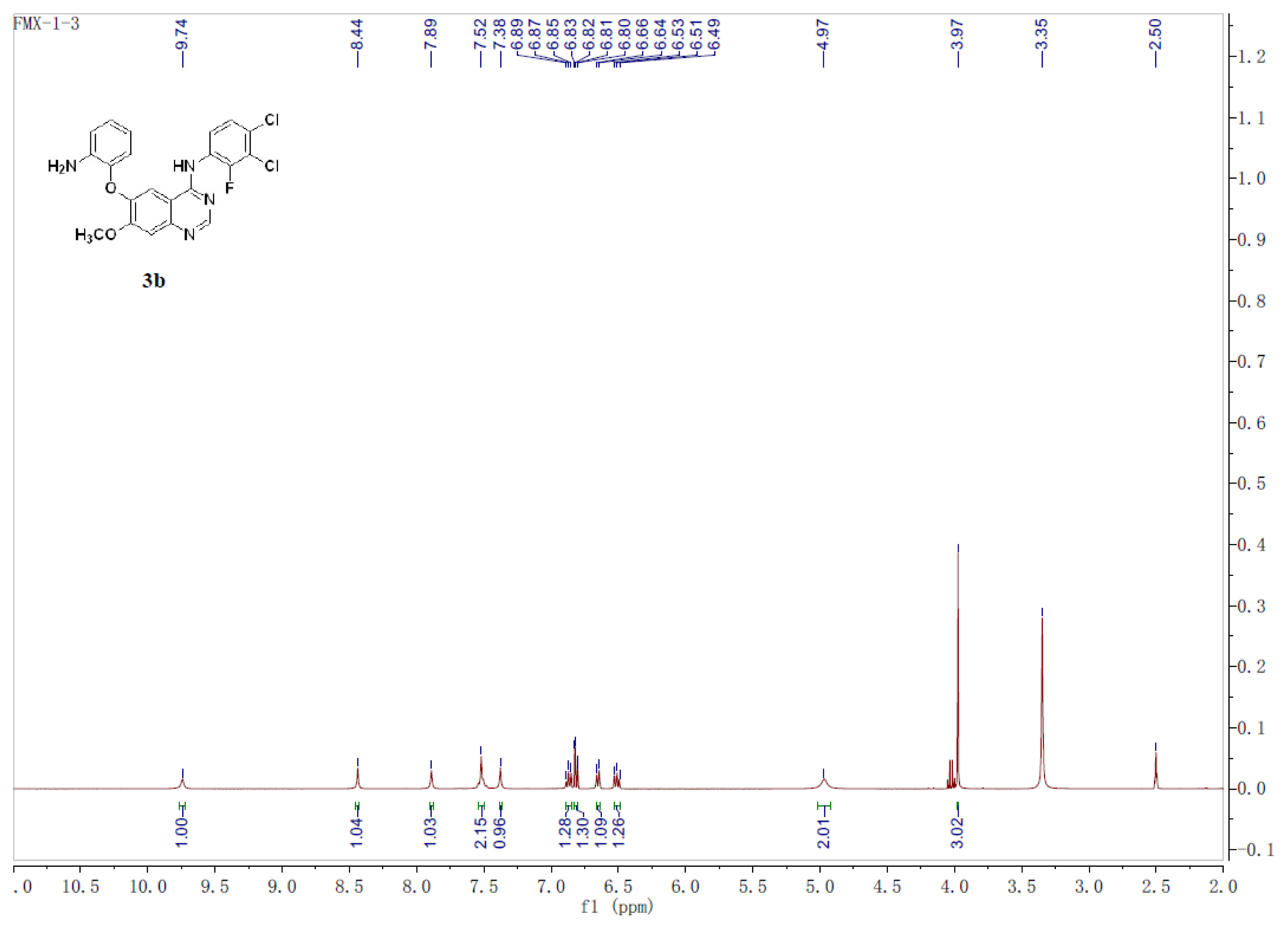

Supplement: Supplementary file 7 — 1HNMR spectrum of 3b [file turkjchem-46-3-849s7.tif]

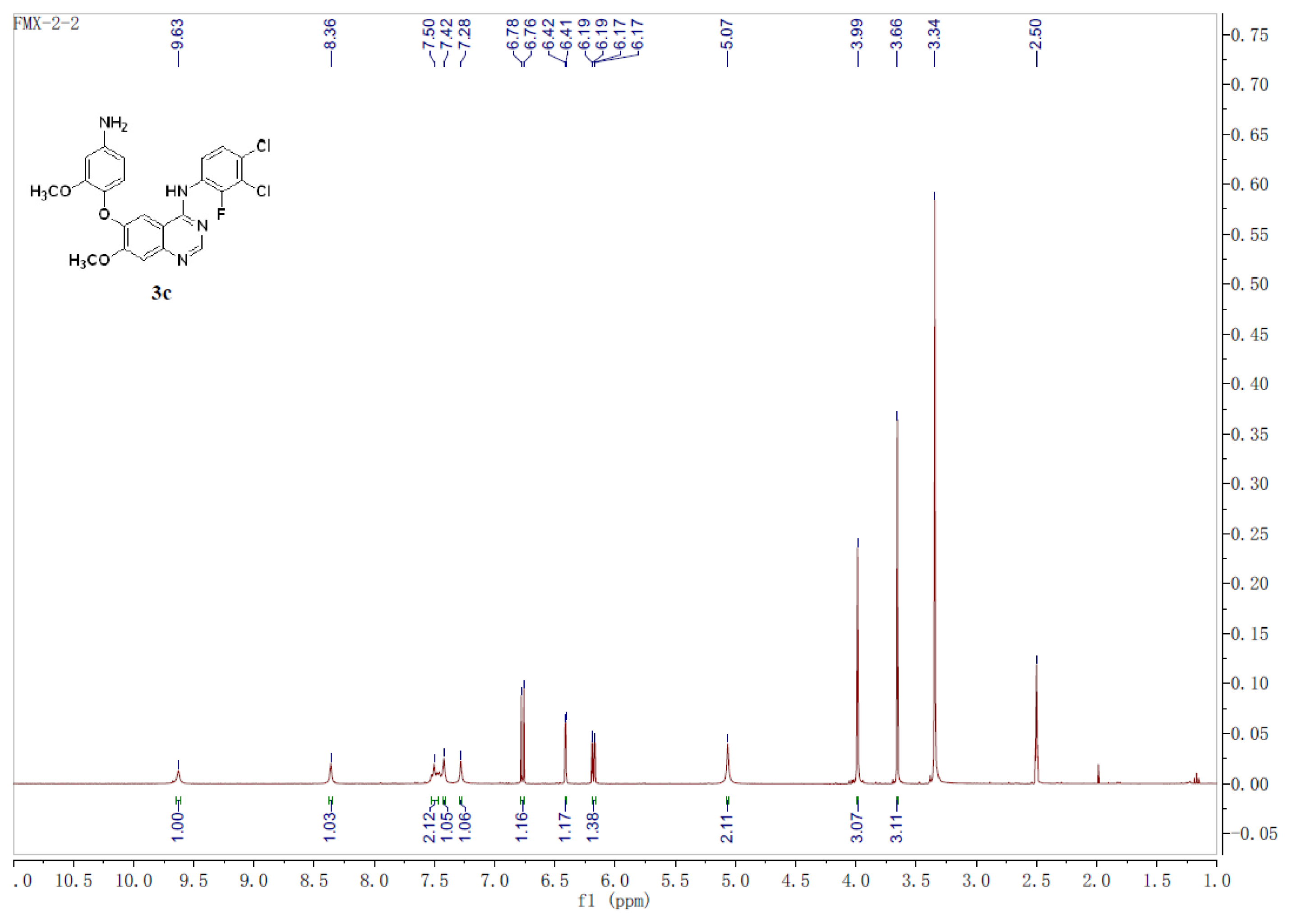

Supplement: Supplementary file 8 — 1HNMR spectrum of 3c [file turkjchem-46-3-849s8.tif]

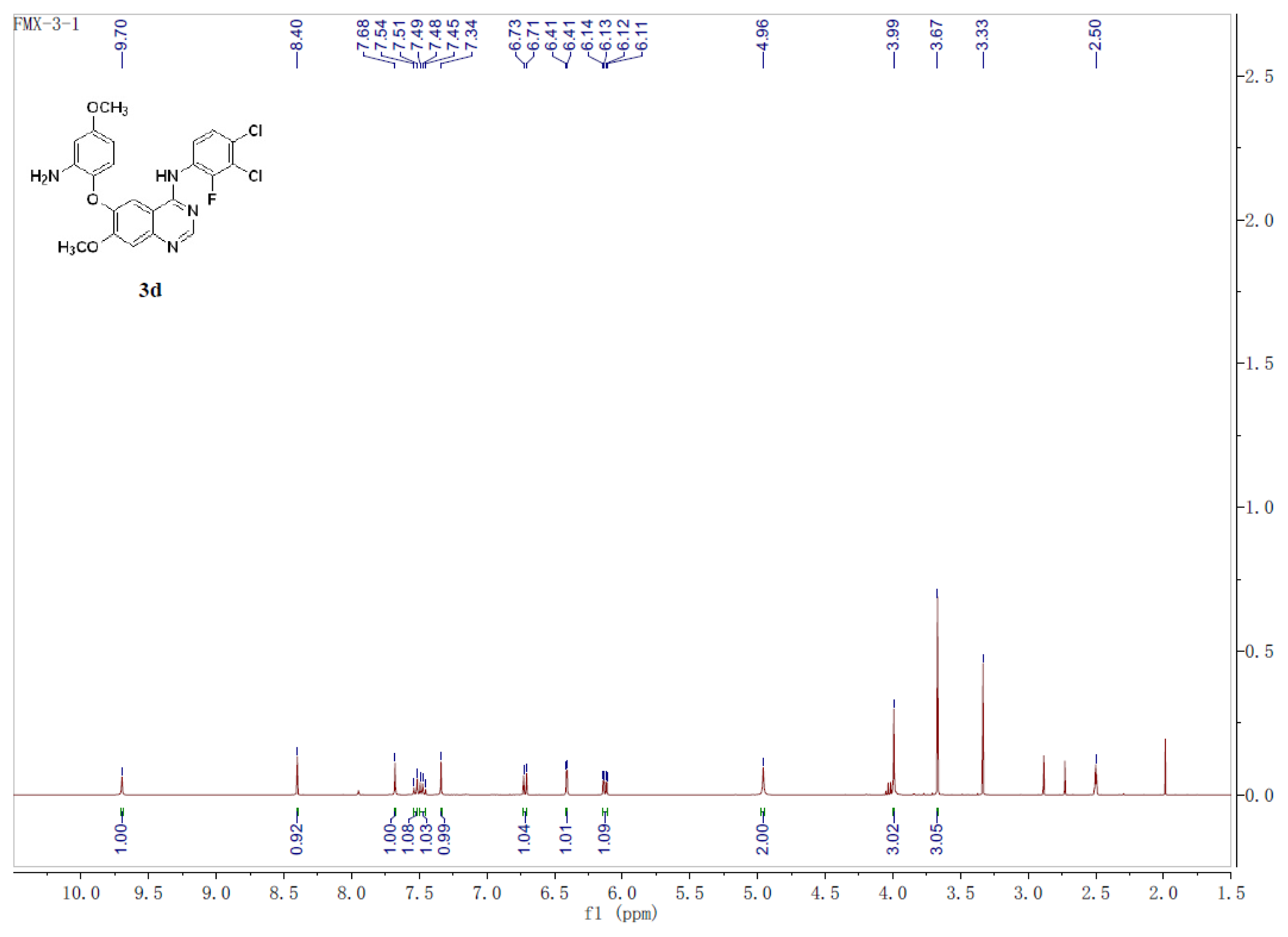

Supplement: Supplementary file 9 — 1HNMR spectrum of 3d [file turkjchem-46-3-849s9.tif]

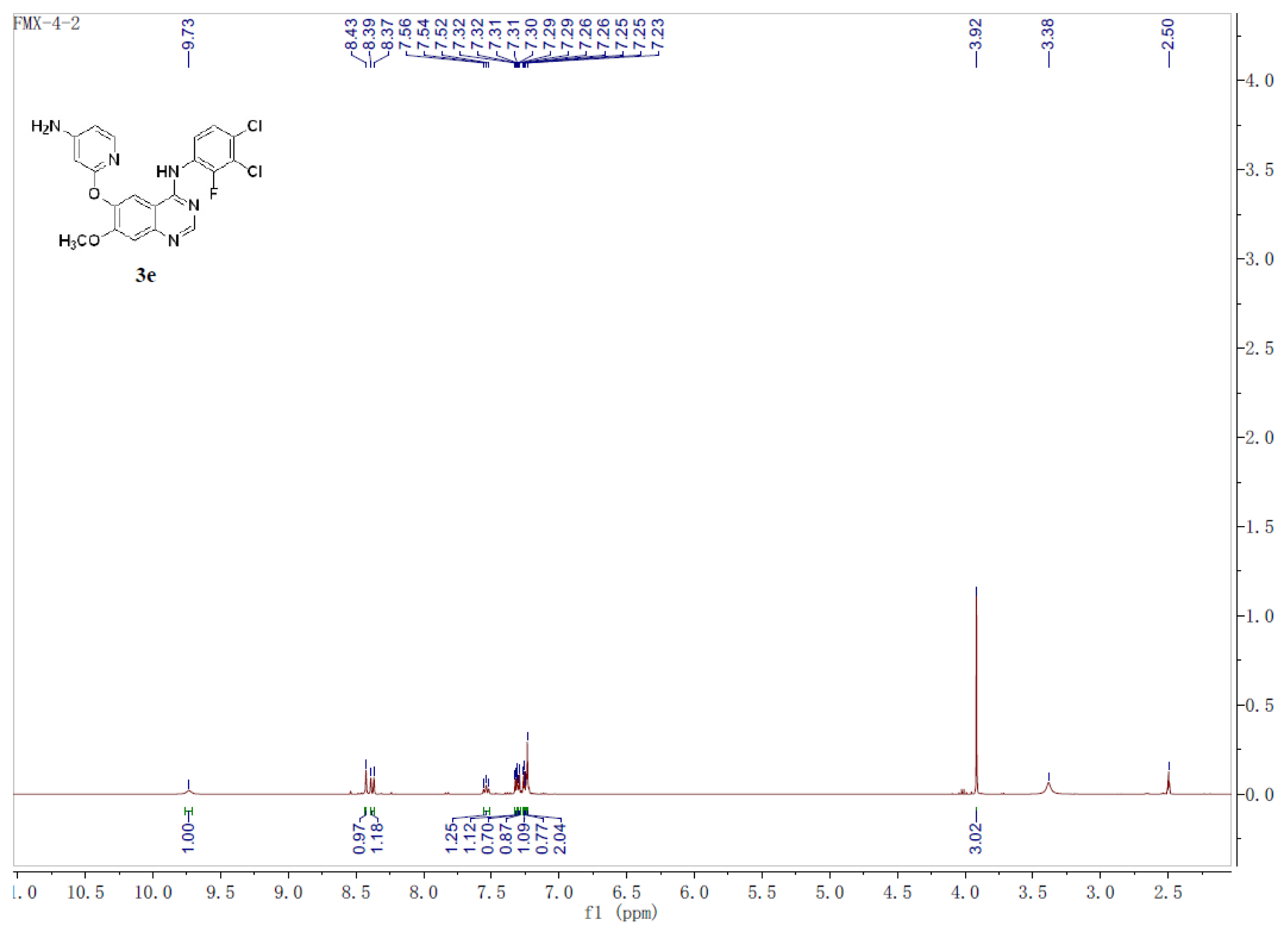

Supplement: Supplementary file 10 — 1HNMR spectrum of 3e [file turkjchem-46-3-849s10.tif]

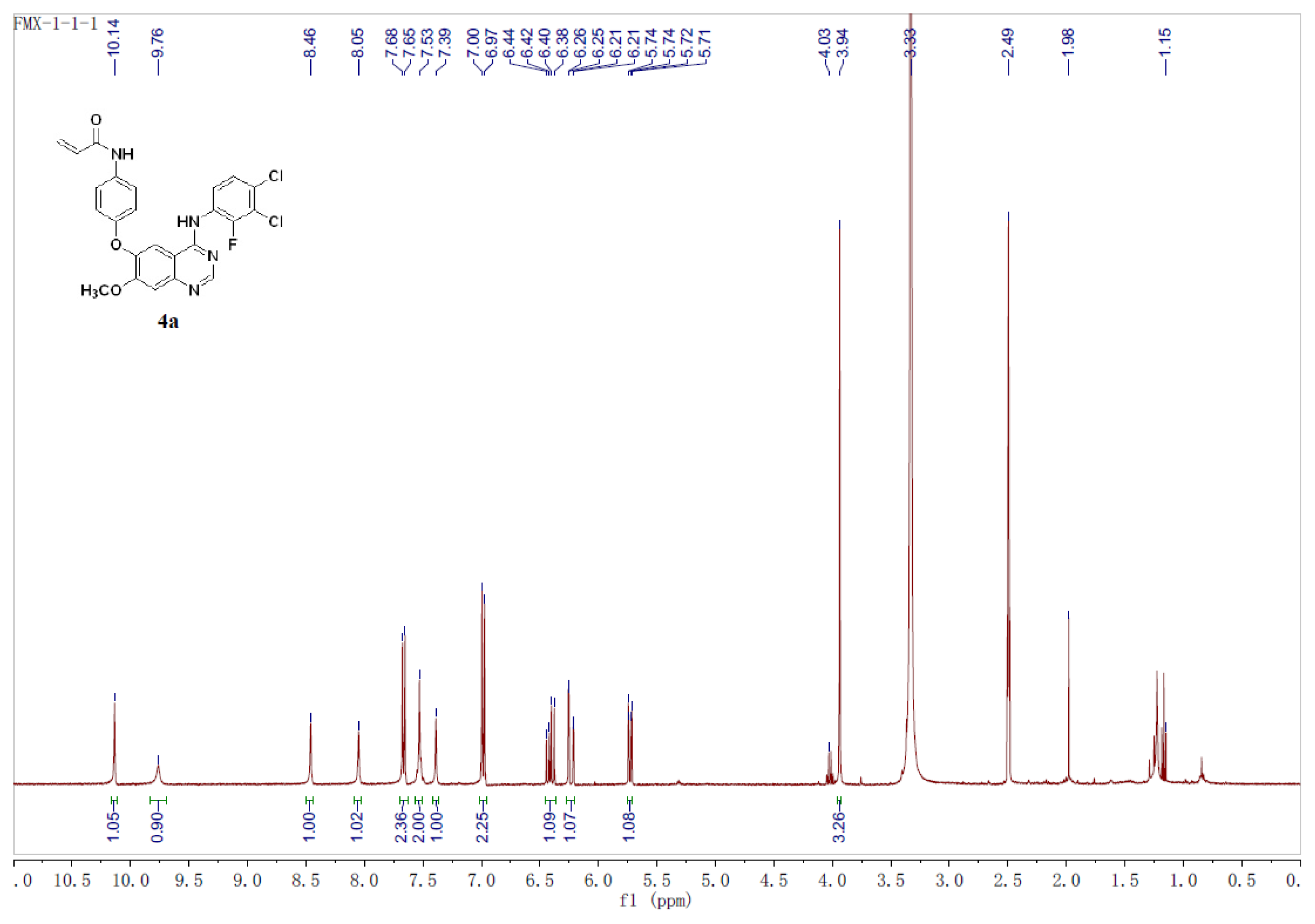

Supplement: Supplementary file 11 — 1H NMR spectrum of 4a [file turkjchem-46-3-849s11.tif]

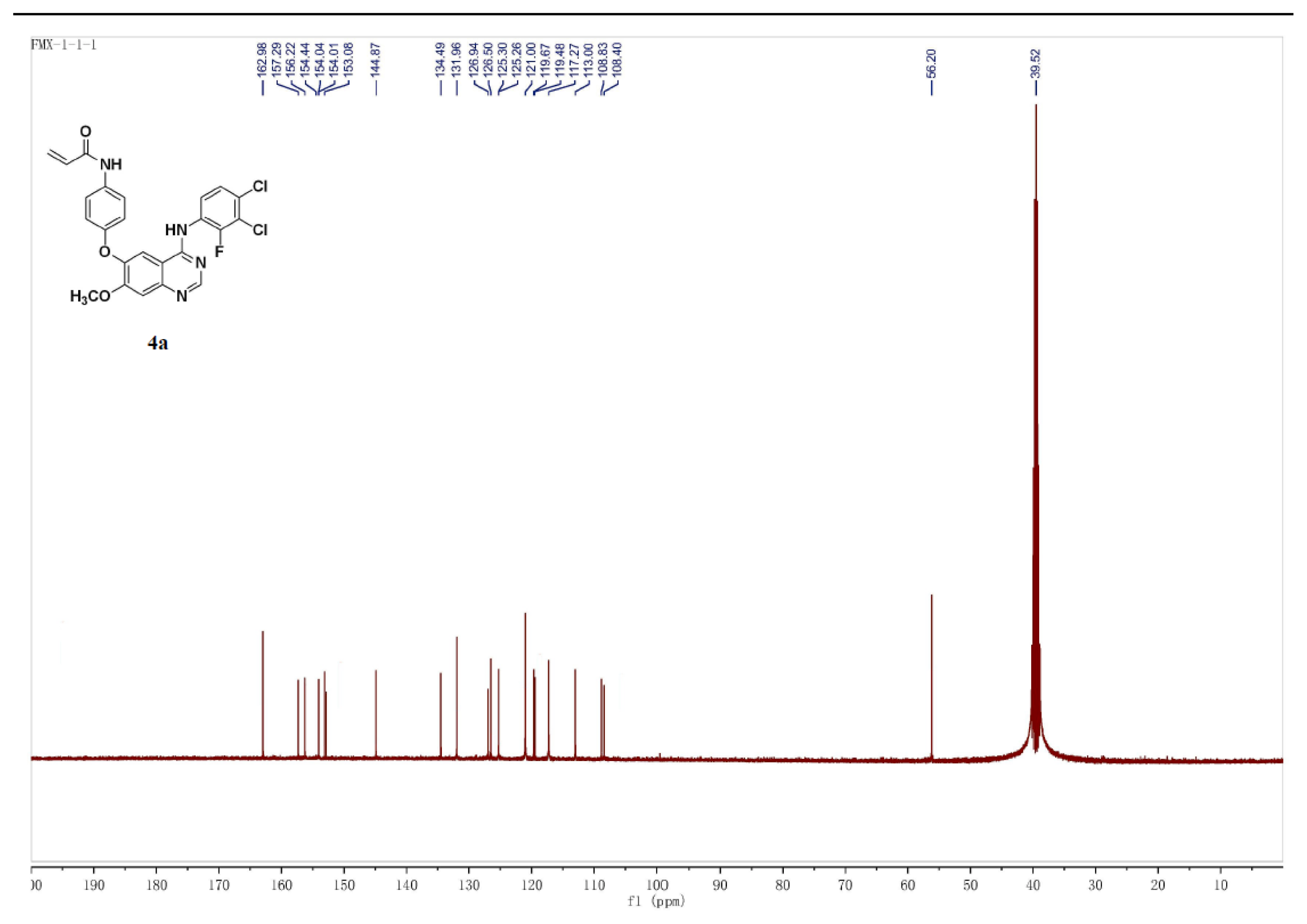

Supplement: Supplementary file 12 — 13C NMR spectrum of 4a [file turkjchem-46-3-849s12.tif]

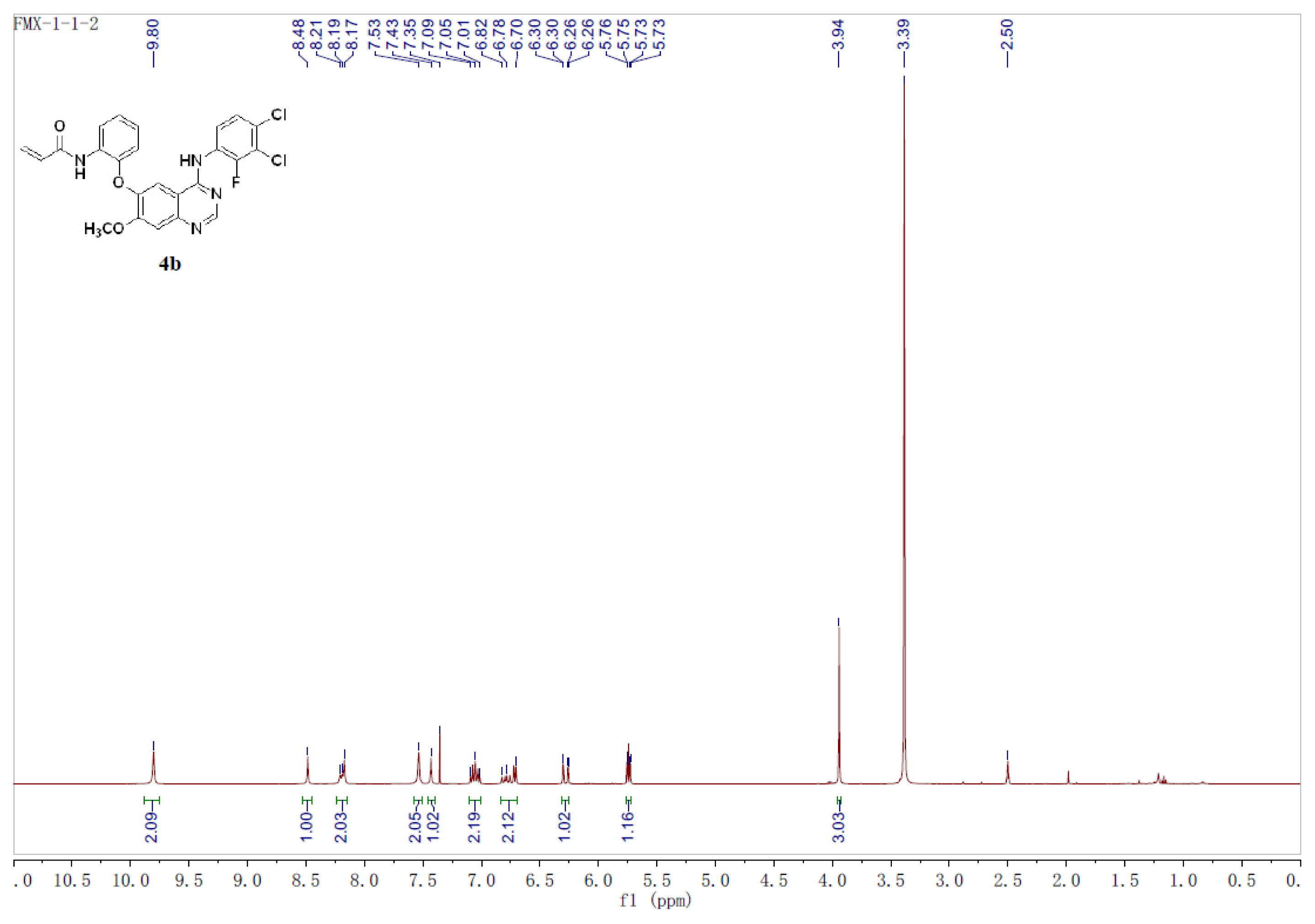

Supplement: Supplementary file 13 — 1H NMR spectrum of 4b [file turkjchem-46-3-849s13.tif]

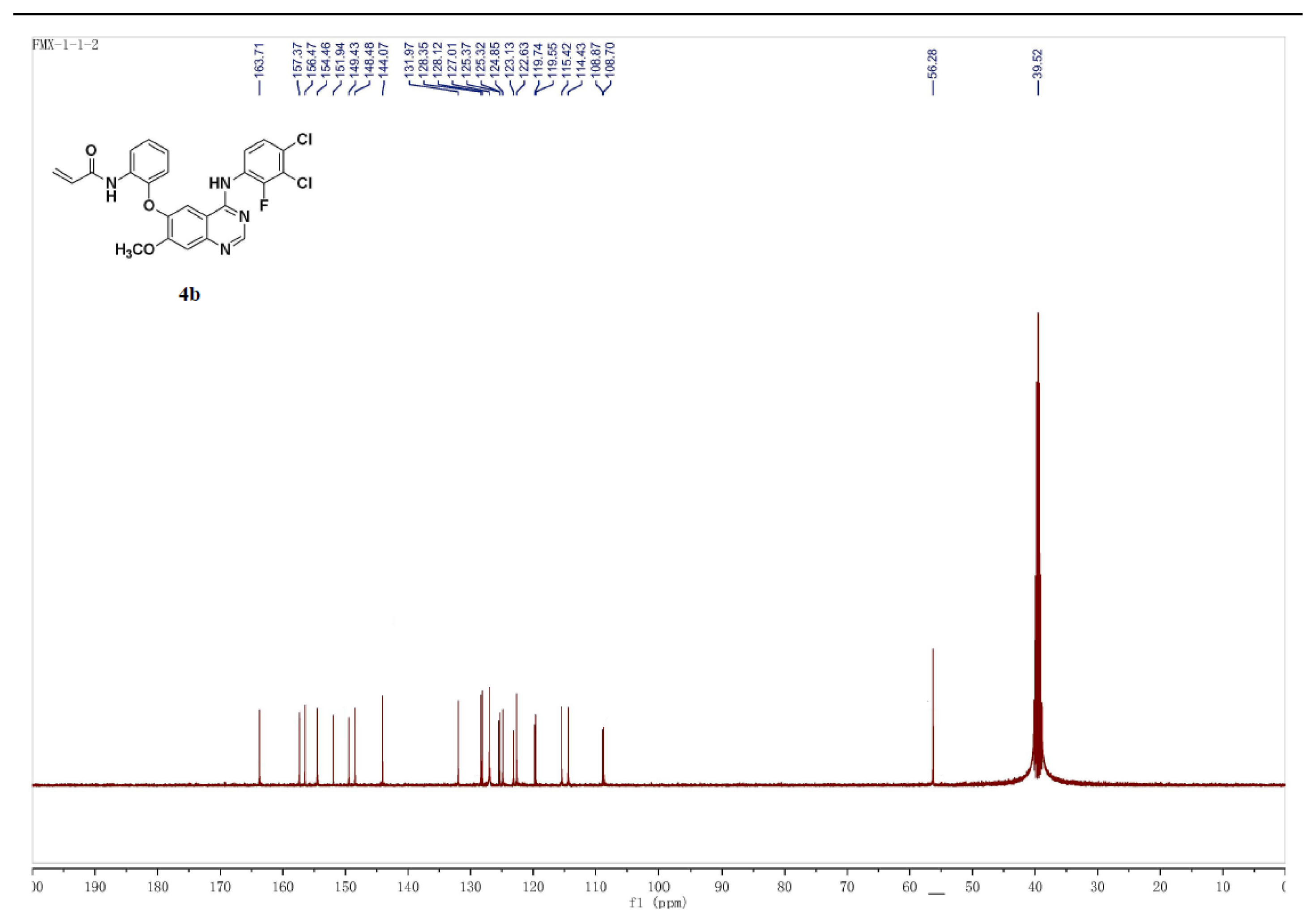

Supplement: Supplementary file 14 — 13C NMR spectrum of 4b [file turkjchem-46-3-849s14.tif]

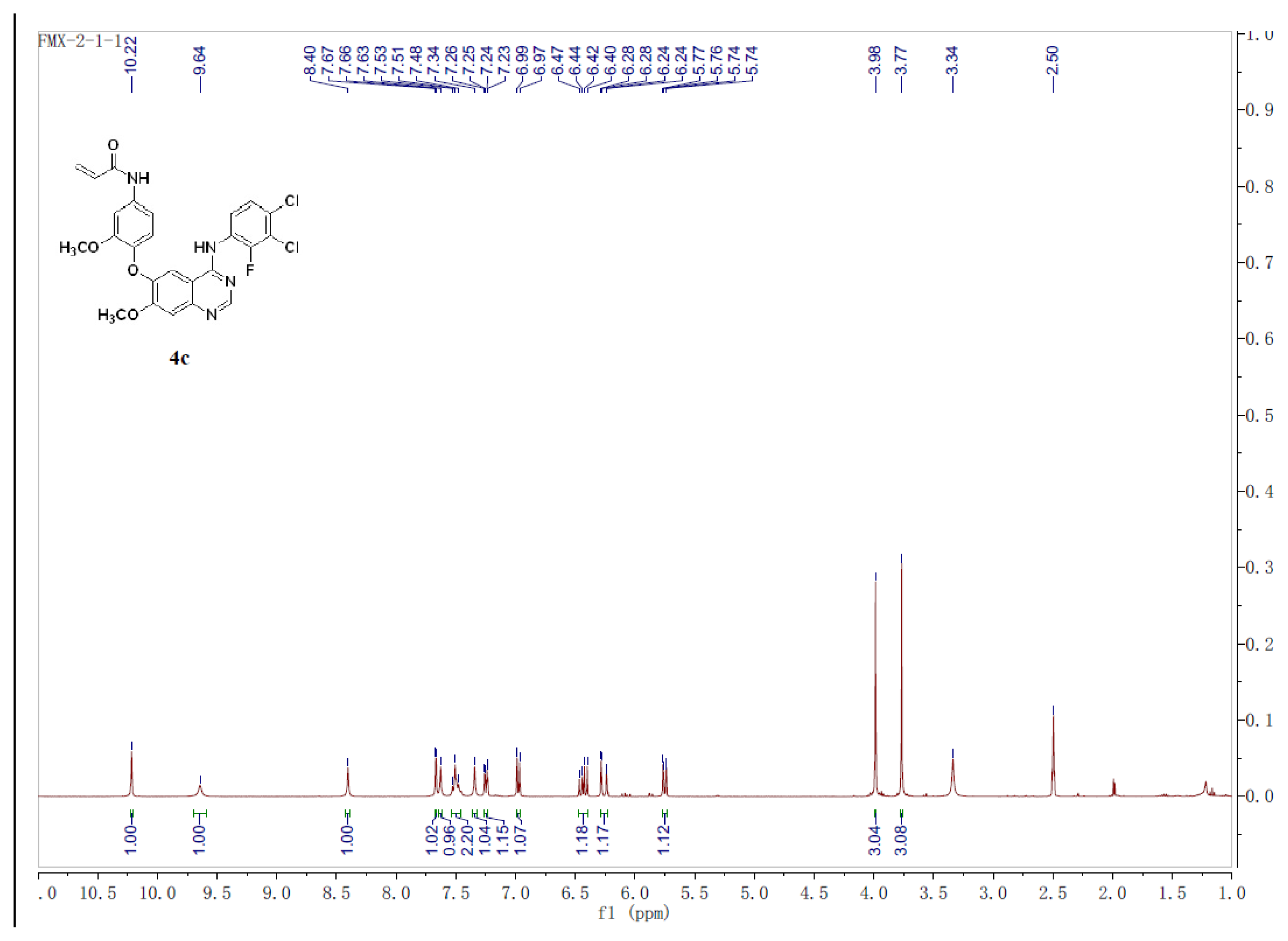

Supplement: Supplementary file 15 — 1H NMR spectrum of 4c [file turkjchem-46-3-849s15.tif]

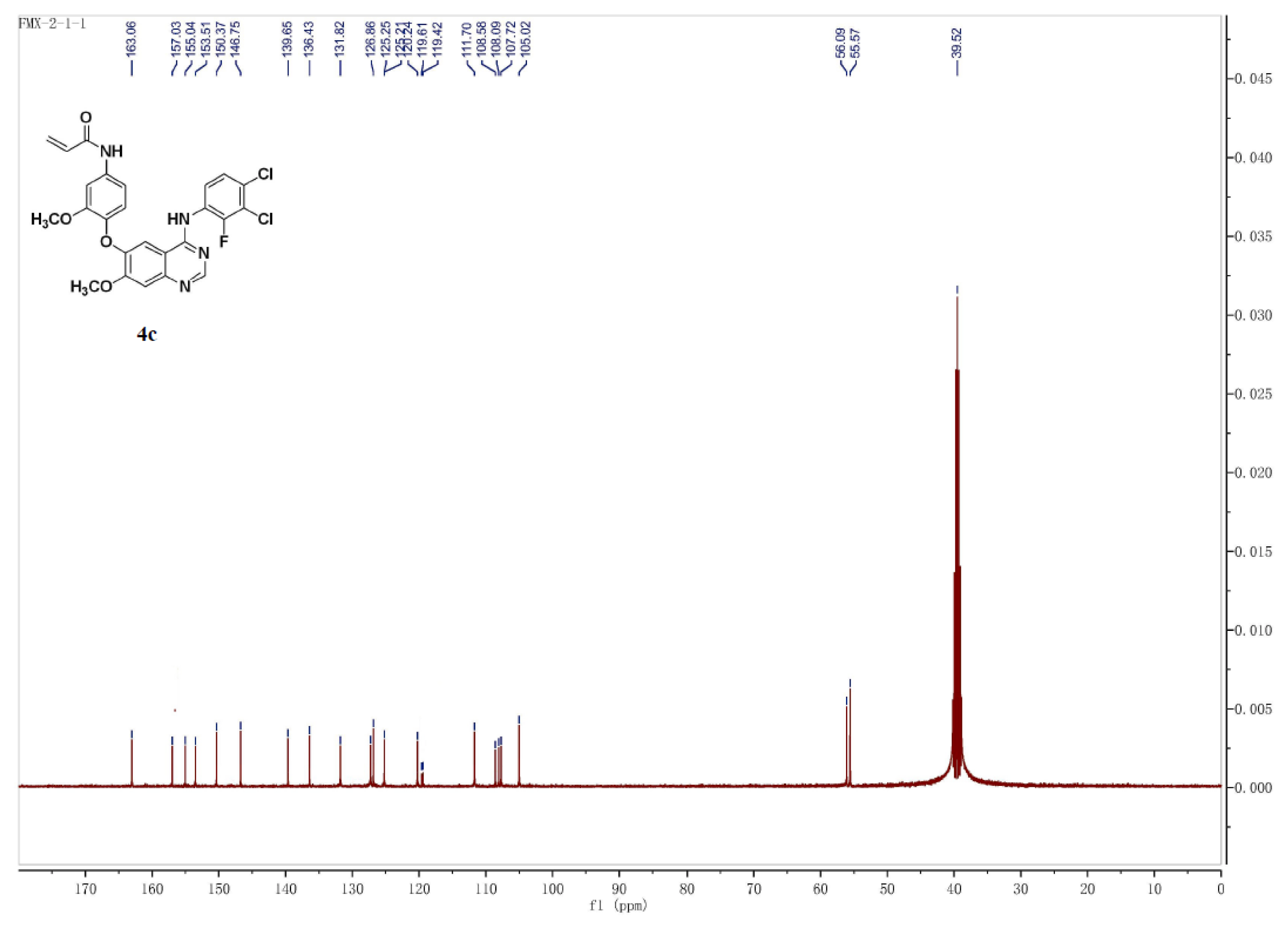

Supplement: Supplementary file 16 — 13C NMR spectrum of 4c [file turkjchem-46-3-849s16.tif]

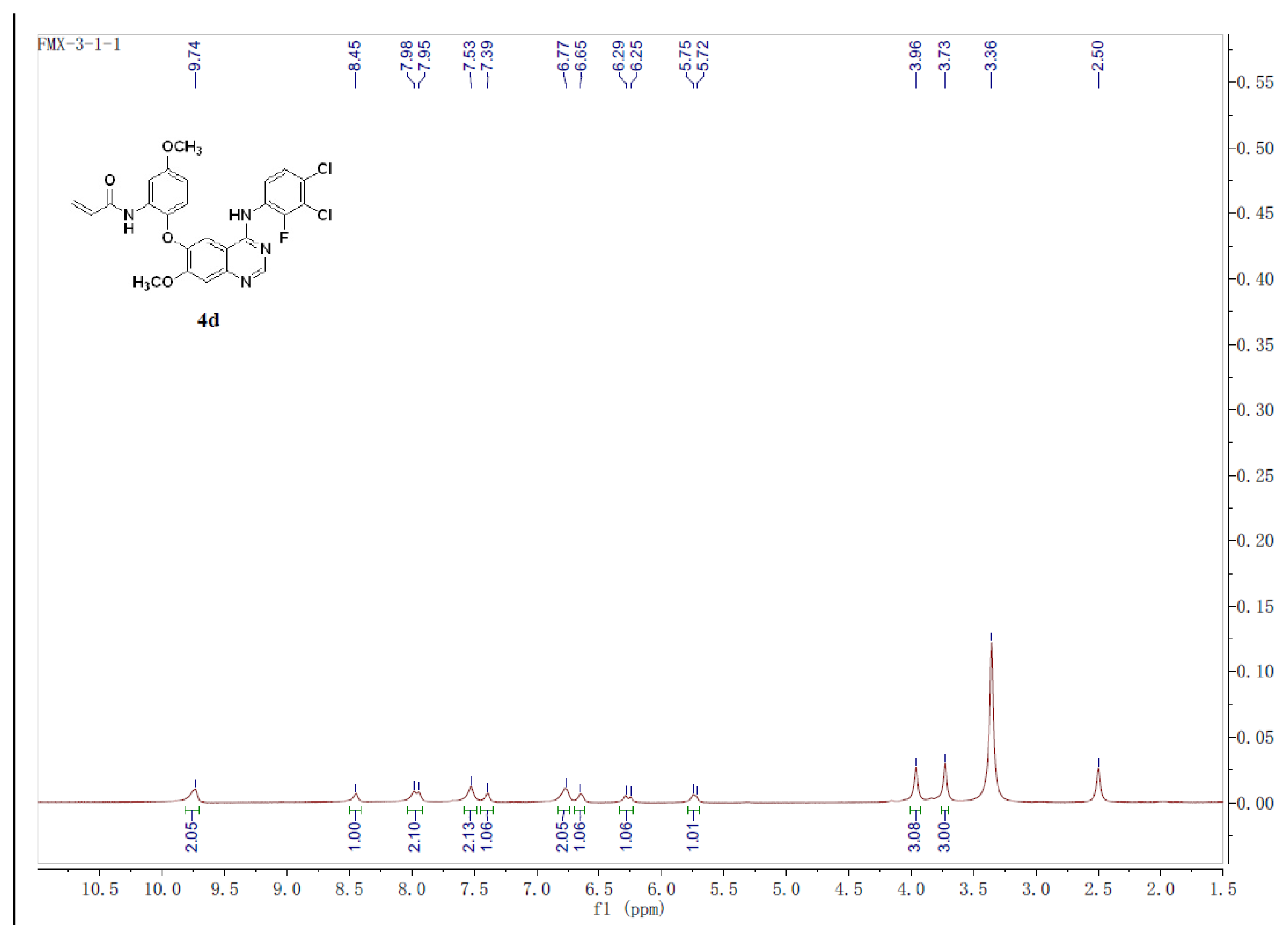

Supplement: Supplementary file 17 — 1H NMR spectrum of 4d [file turkjchem-46-3-849s17.tif]

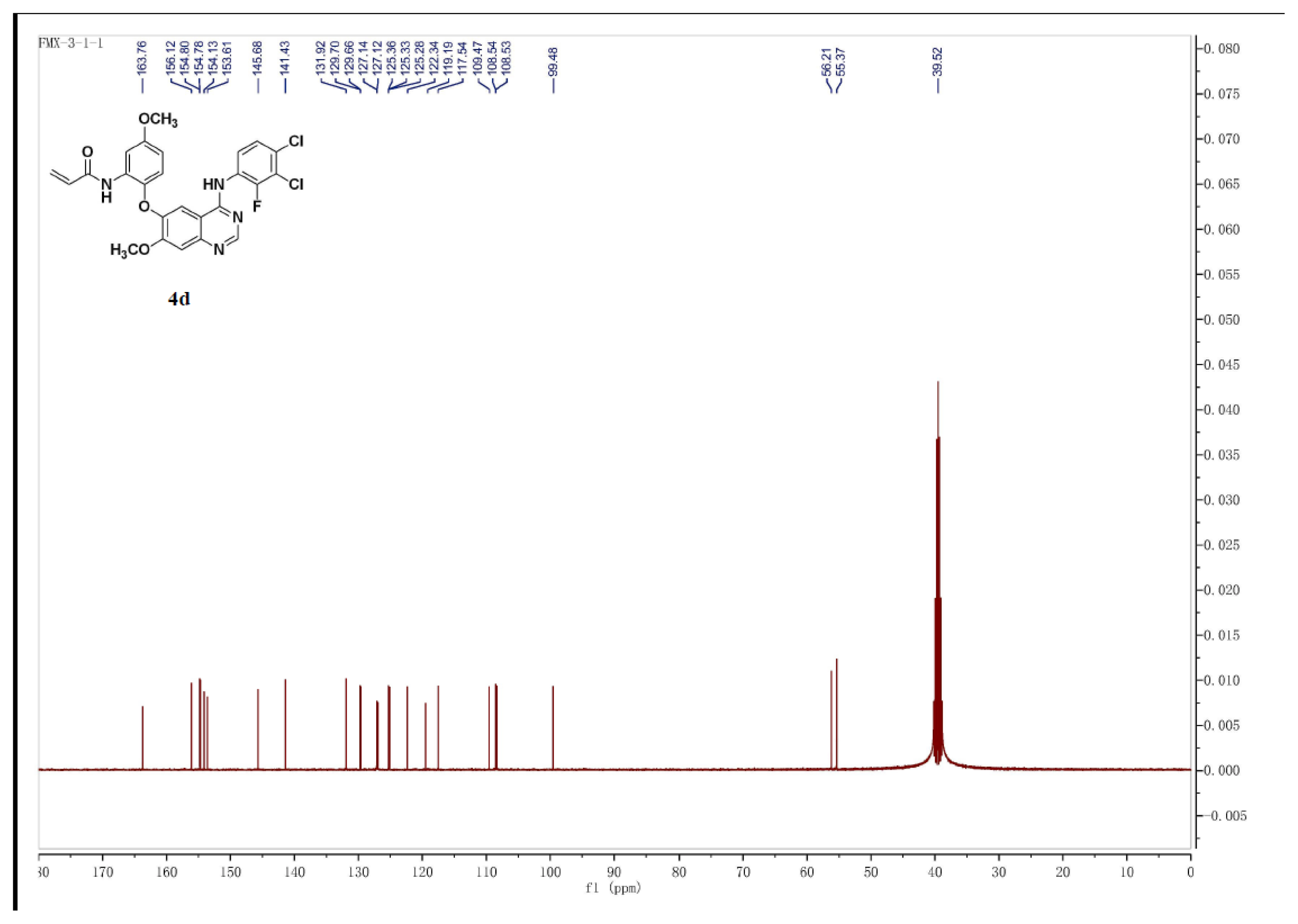

Supplement: Supplementary file 18 — 13C NMR spectrum of 4d [file turkjchem-46-3-849s18.tif]

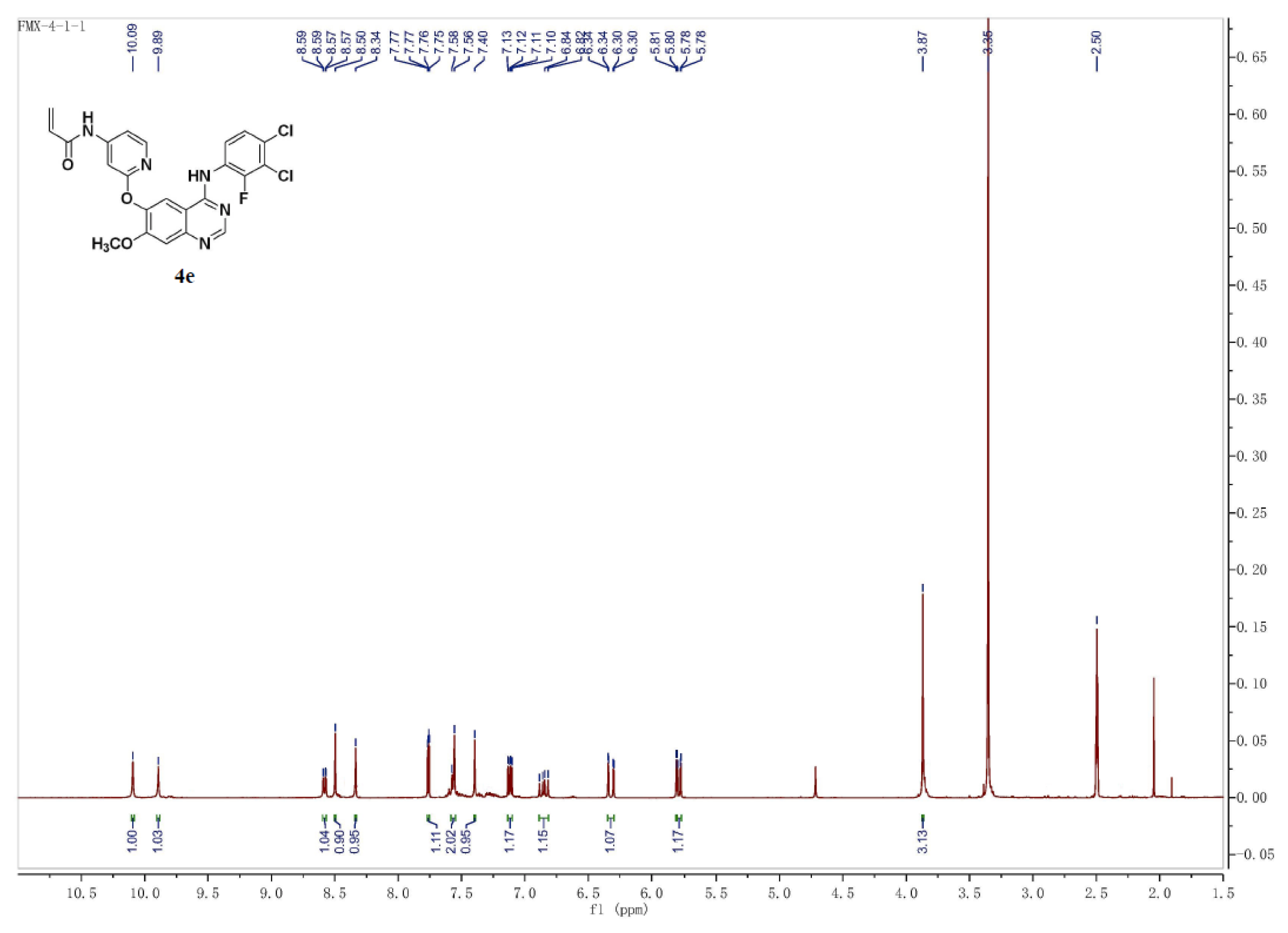

Supplement: Supplementary file 19 — 1H NMR spectrum of 4e [file turkjchem-46-3-849s19.tif]

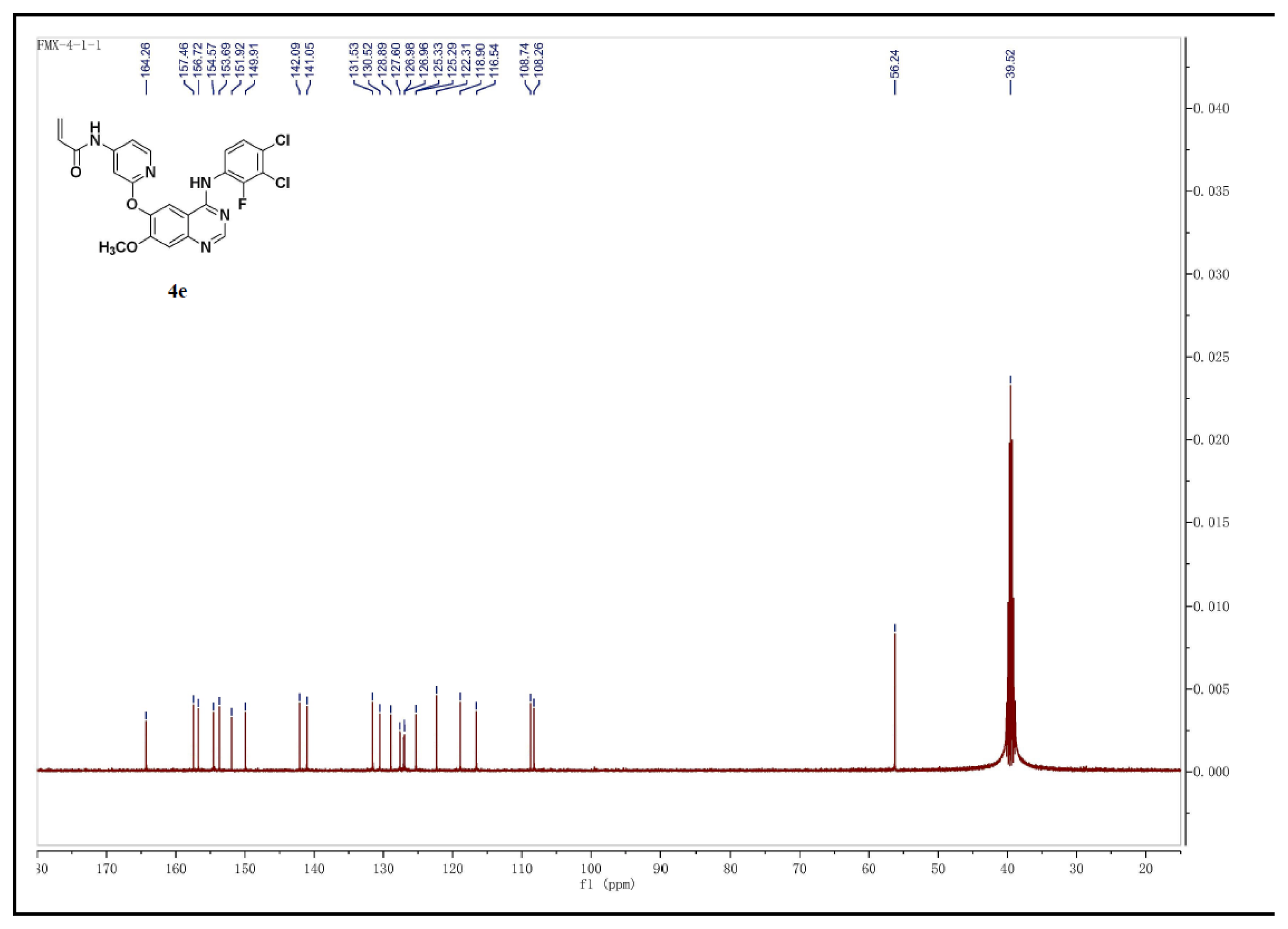

Supplement: Supplementary file 20 — 13C NMR spectrum of 4e [file turkjchem-46-3-849s20.tif]

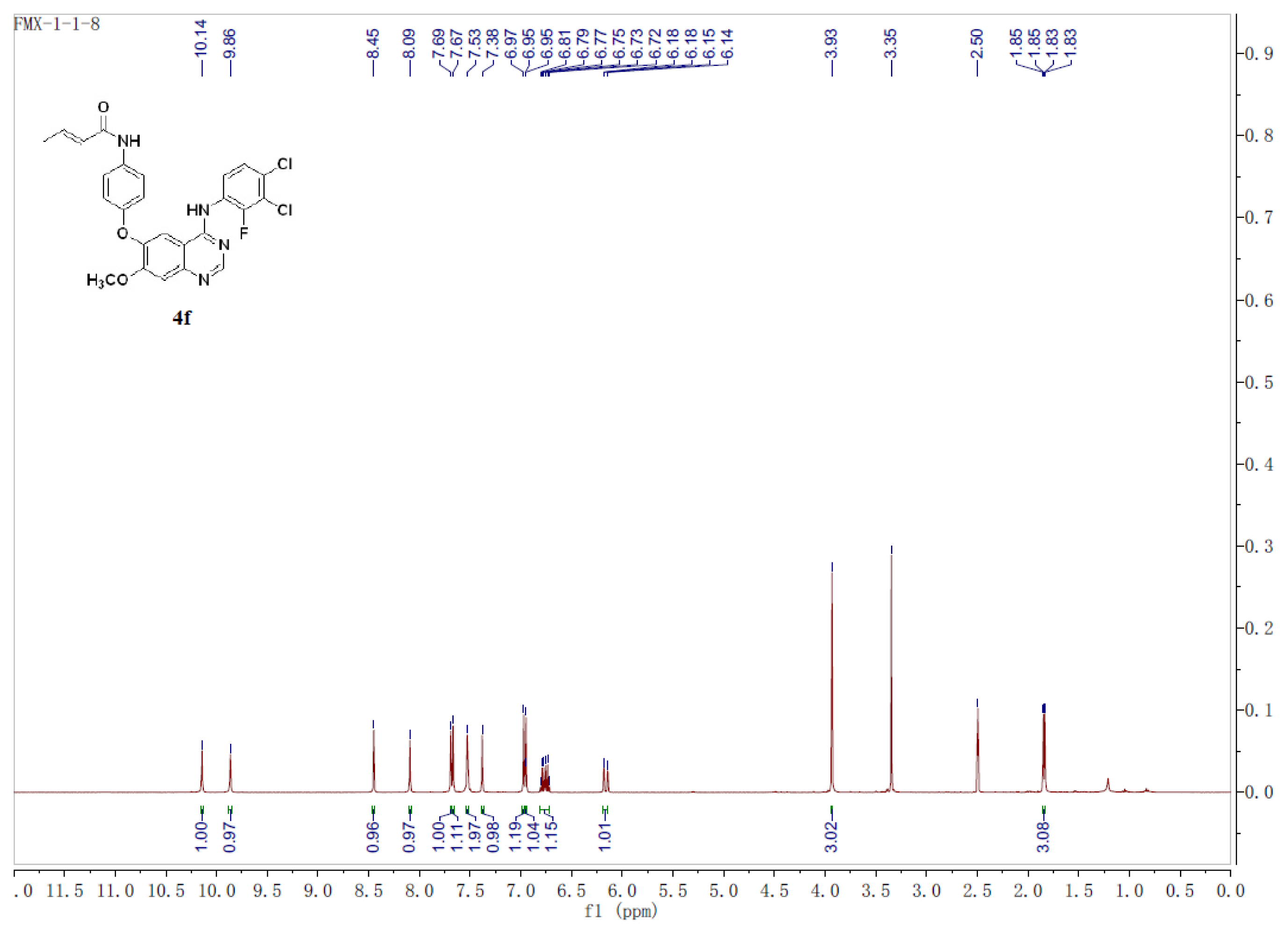

Supplement: Supplementary file 21 — 1H NMR spectrum of 4f [file turkjchem-46-3-849s21.tif]

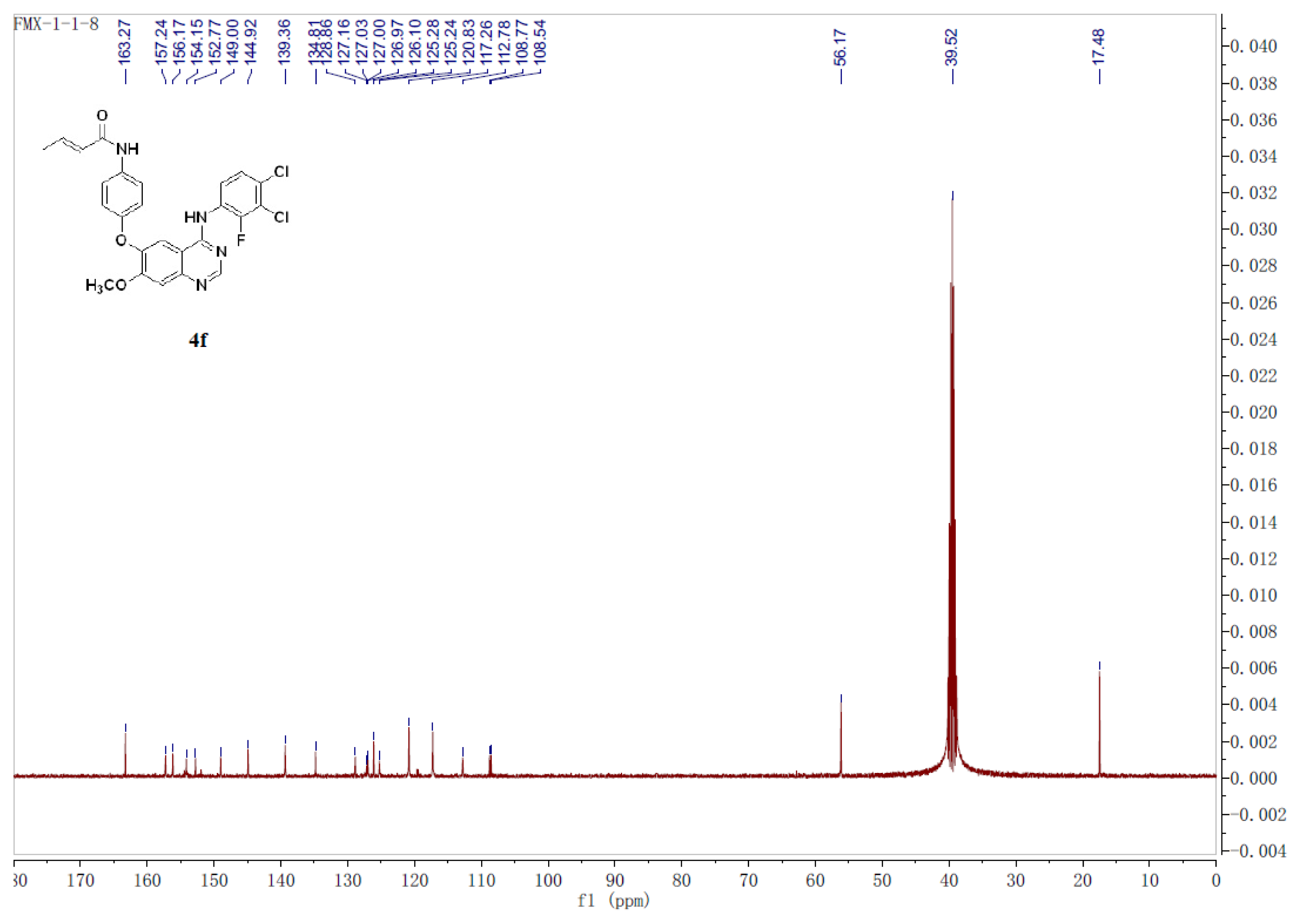

Supplement: Supplementary file 22 — 13C NMR spectrum of 4f [file turkjchem-46-3-849s22.tif]

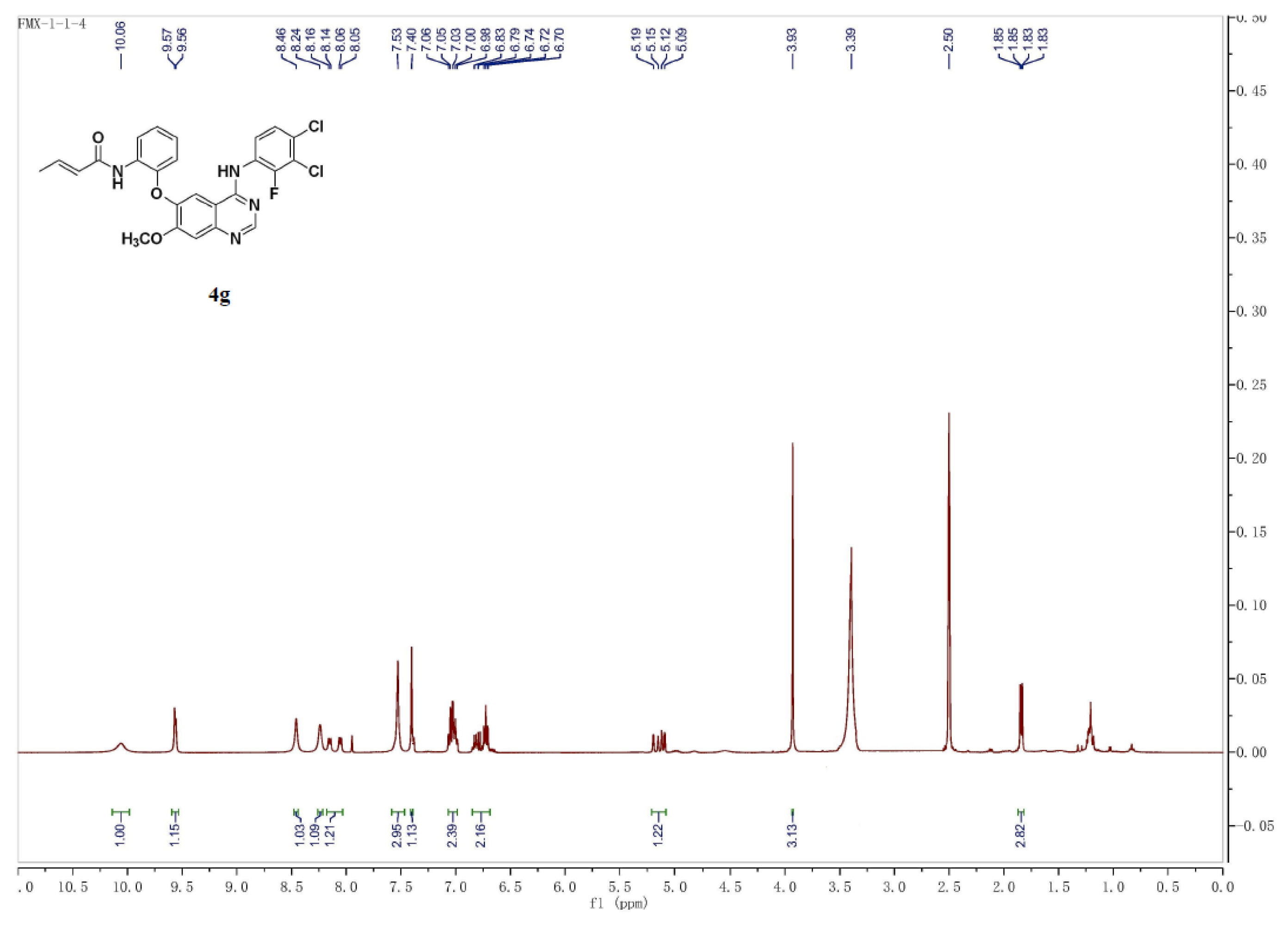

Supplement: Supplementary file 23 — 1H NMR spectrum of 4g [file turkjchem-46-3-849s23.tif]

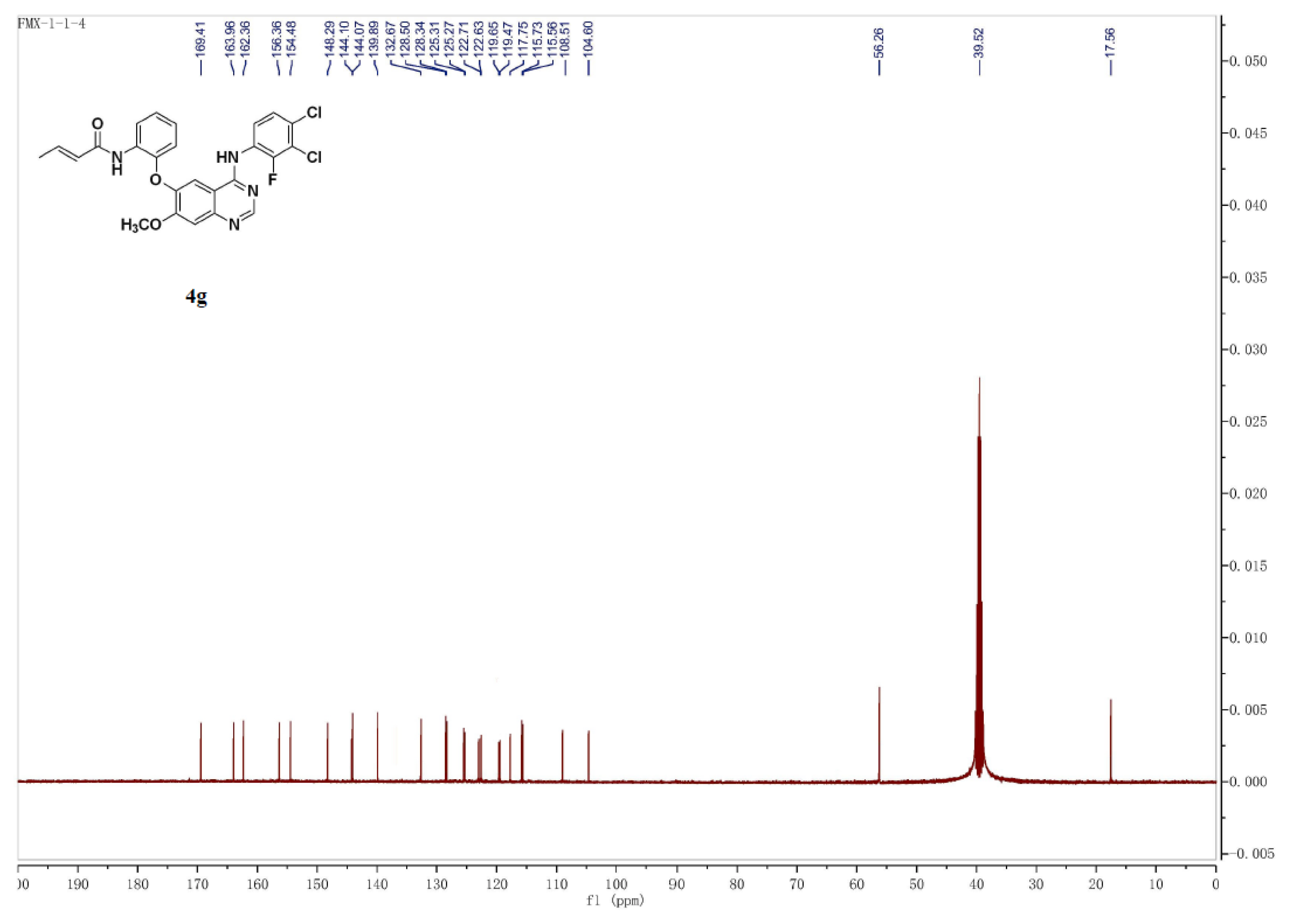

Supplement: Supplementary file 24 — 13C NMR spectrum of 4g [file turkjchem-46-3-849s24.tif]

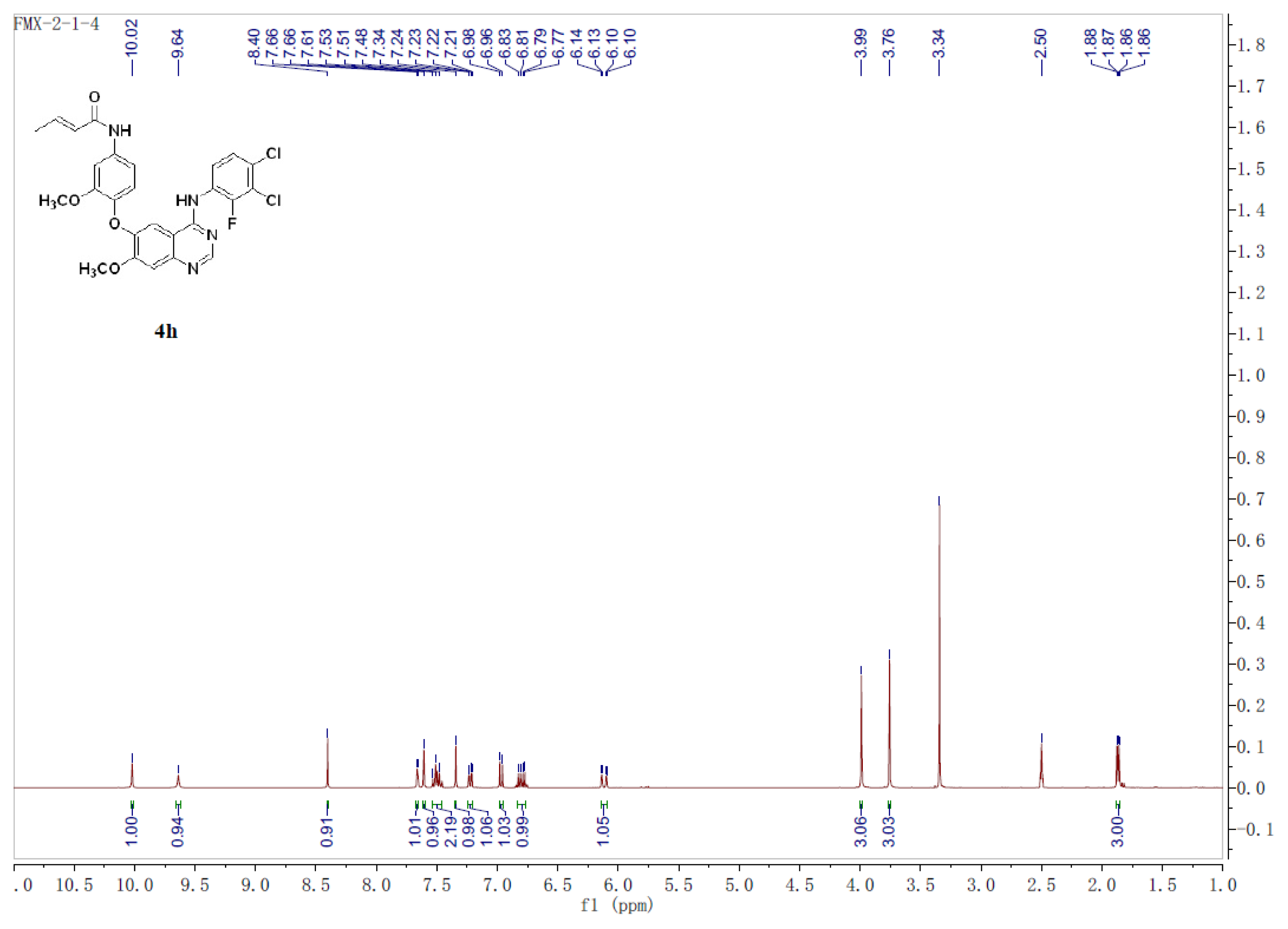

Supplement: Supplementary file 25 — 1H NMR spectrum of 4h [file turkjchem-46-3-849s25.tif]

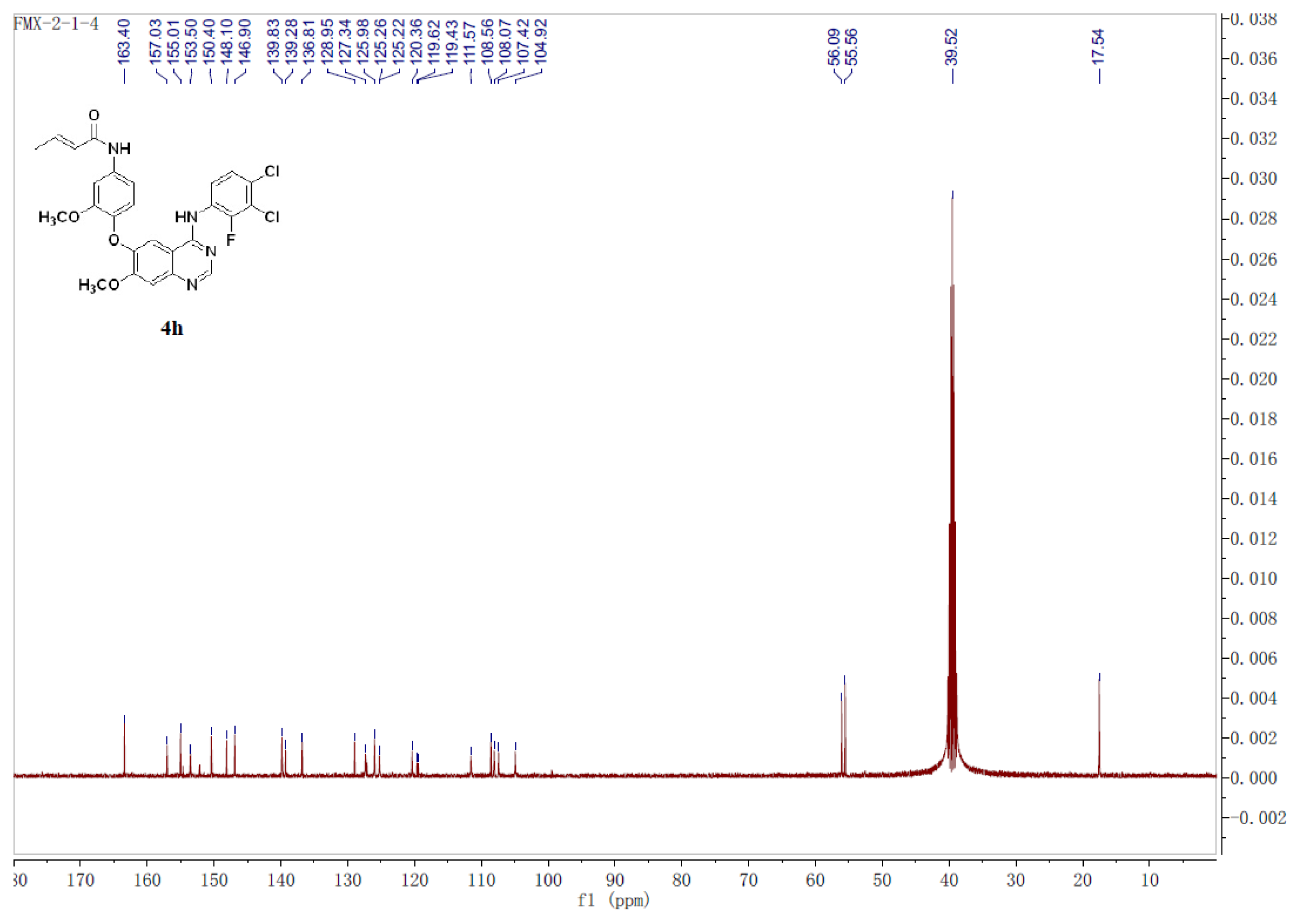

Supplement: Supplementary file 26 — 13C NMR spectrum of 4h [file turkjchem-46-3-849s26.tif]

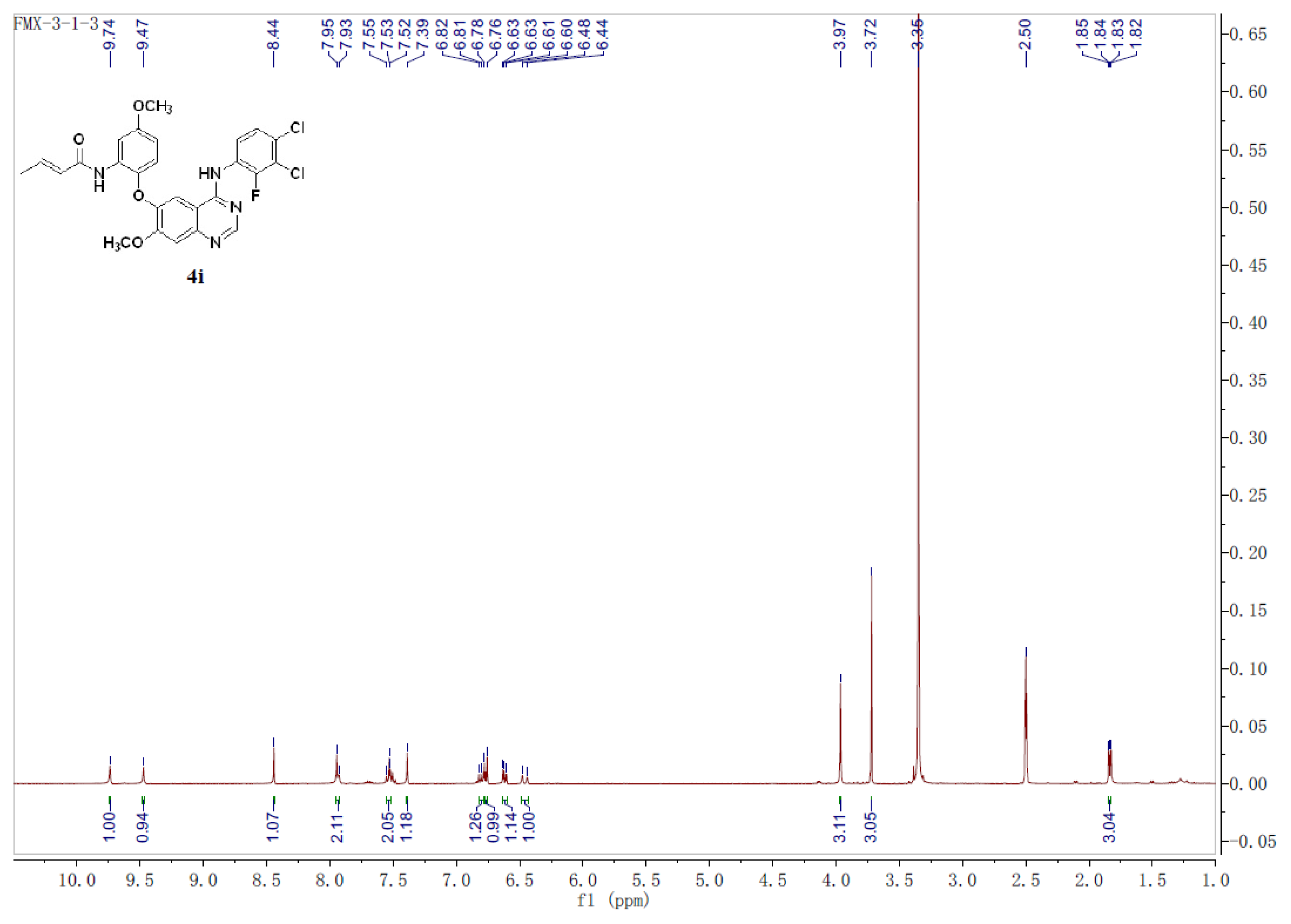

Supplement: Supplementary file 27 — 1H NMR spectrum of 4i [file turkjchem-46-3-849s27.tif]

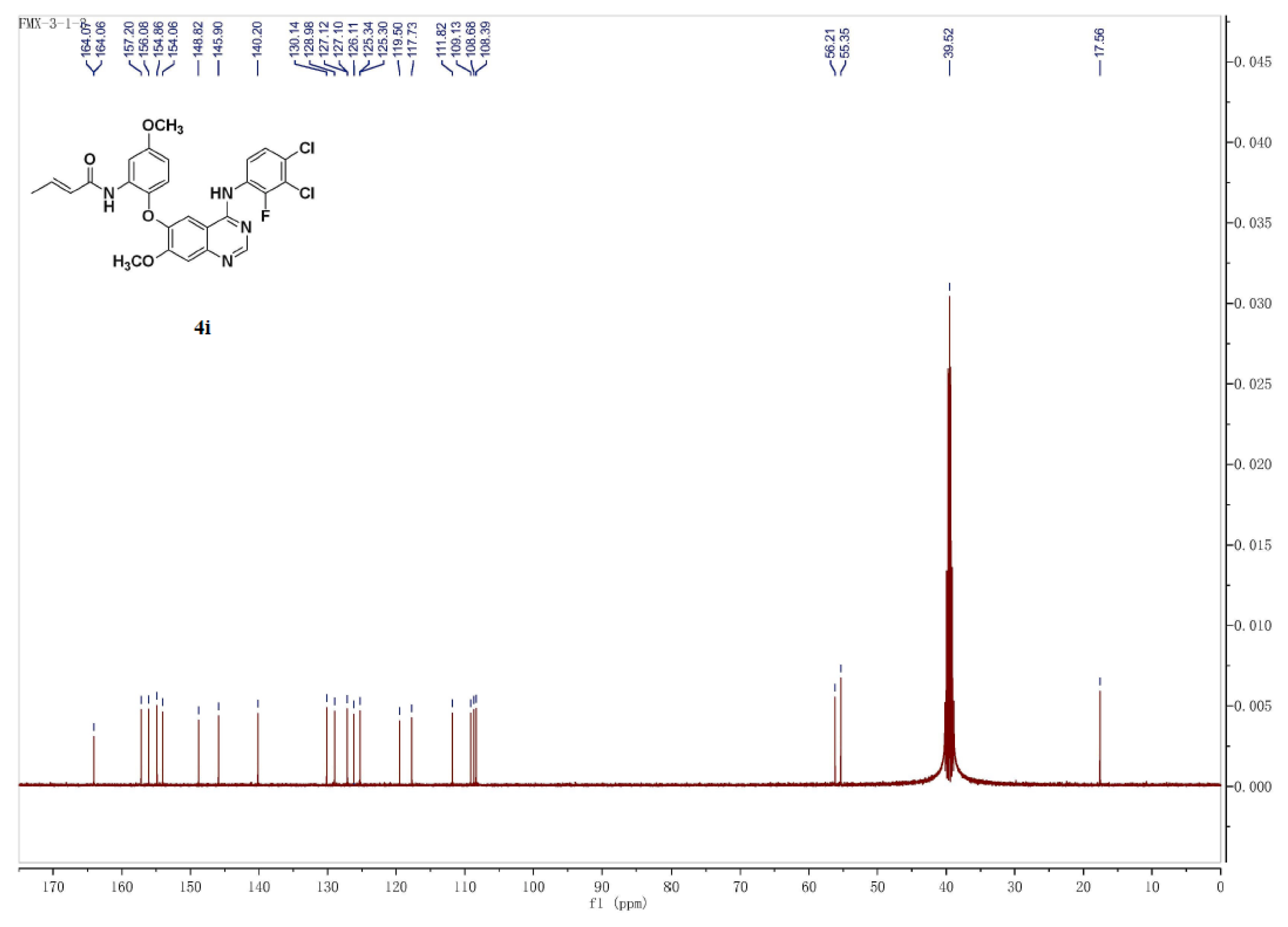

Supplement: Supplementary file 28 — 13C NMR spectrum of 4i [file turkjchem-46-3-849s28.tif]

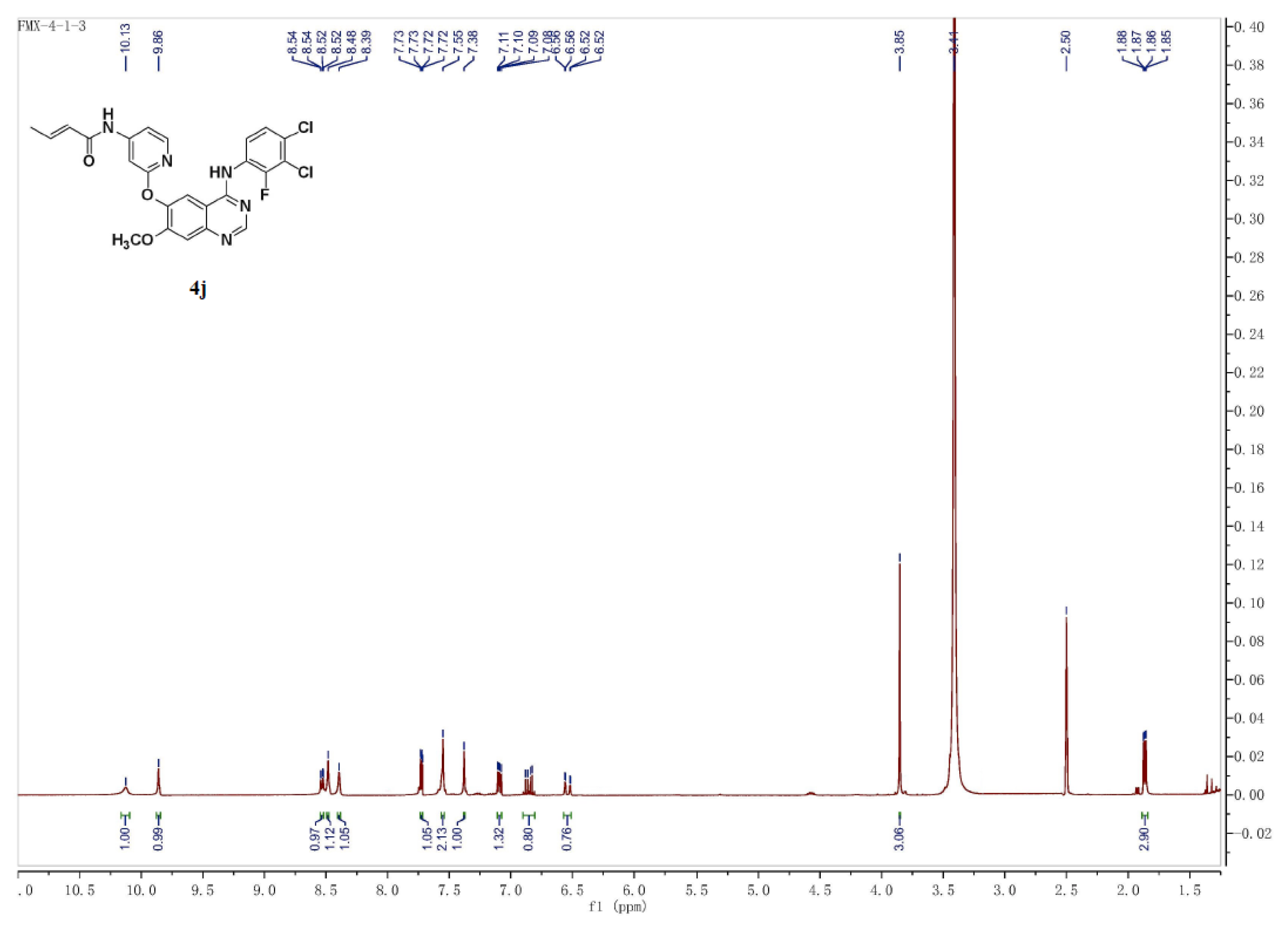

Supplement: Supplementary file 29 — 1H NMR spectrum of 4j [file turkjchem-46-3-849s29.tif]

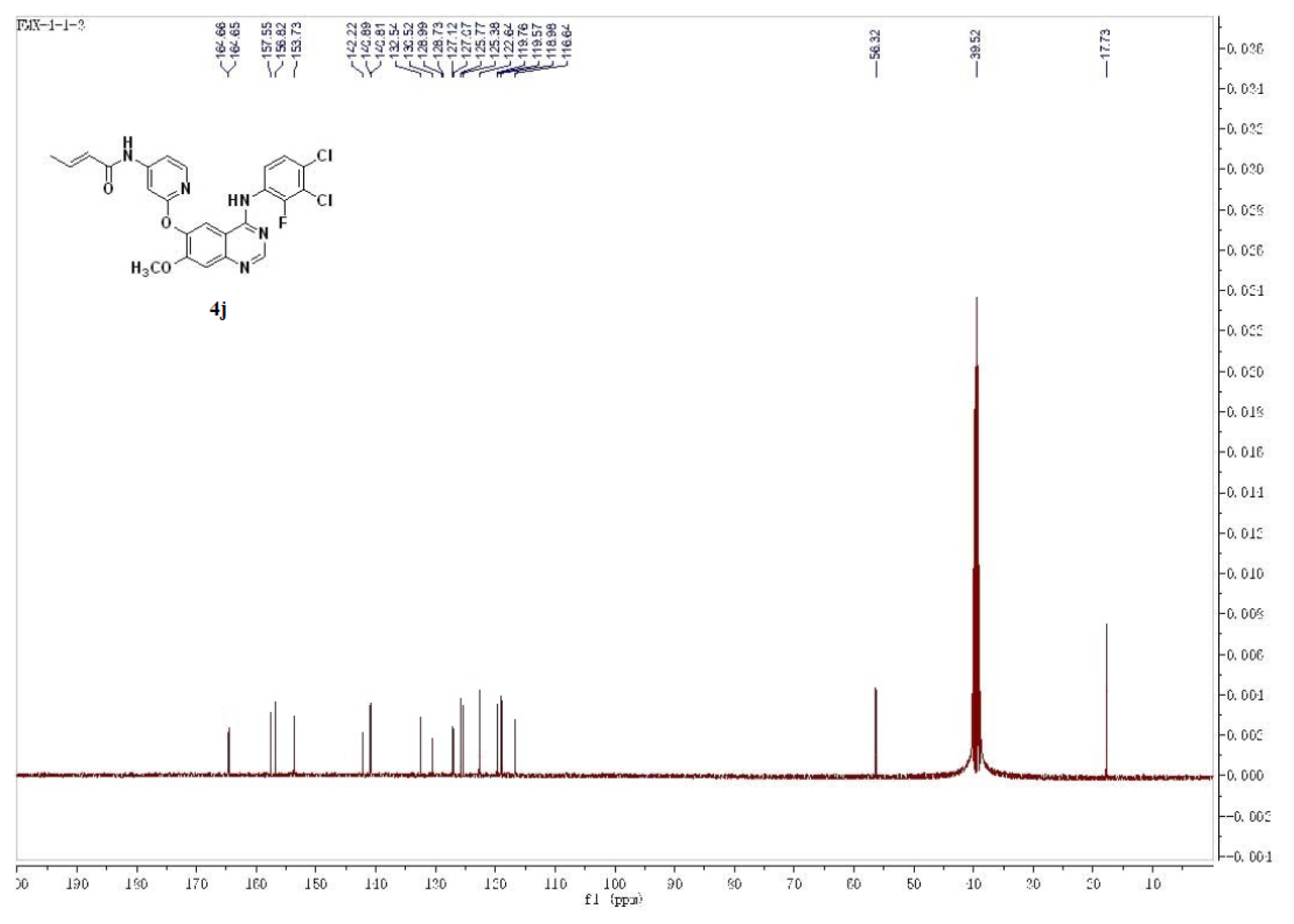

Supplement: Supplementary file 30 — 13C NMR spectrum of 4j [file turkjchem-46-3-849s30.tif]

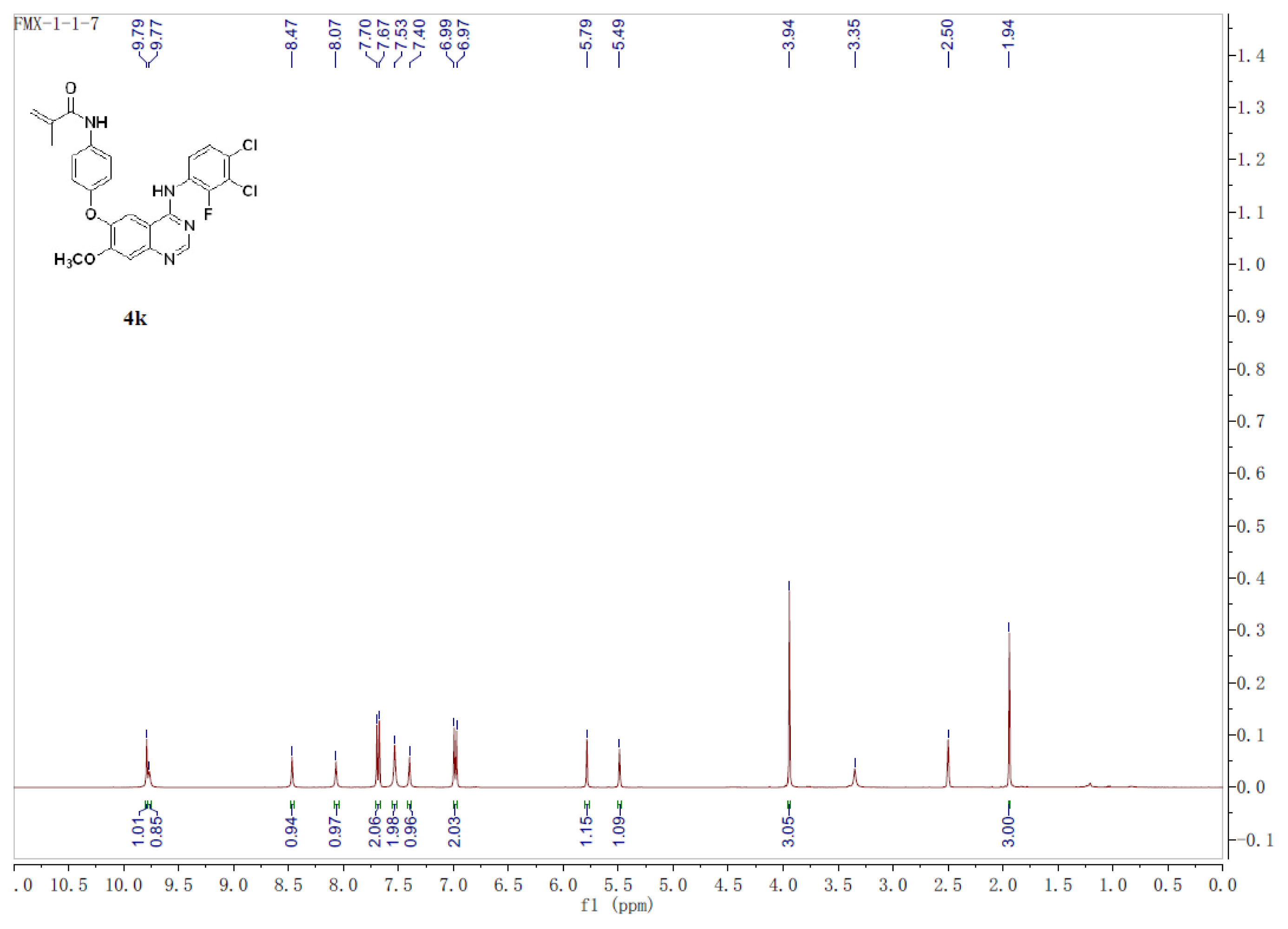

Supplement: Supplementary file 31 — 1H NMR spectrum of 4k [file turkjchem-46-3-849s31.tif]

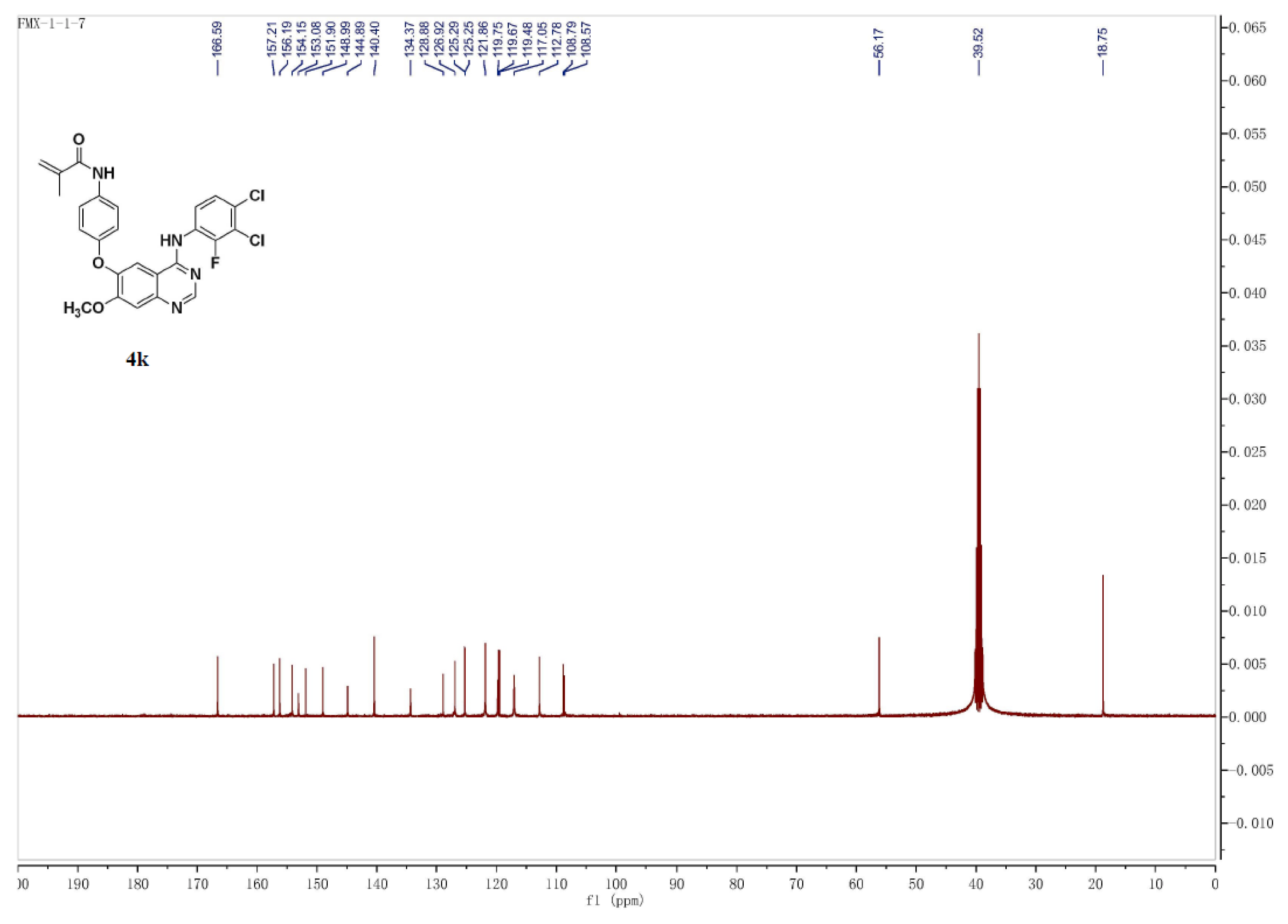

Supplement: Supplementary file 32 — 13C NMR spectrum of 4k [file turkjchem-46-3-849s32.tif]

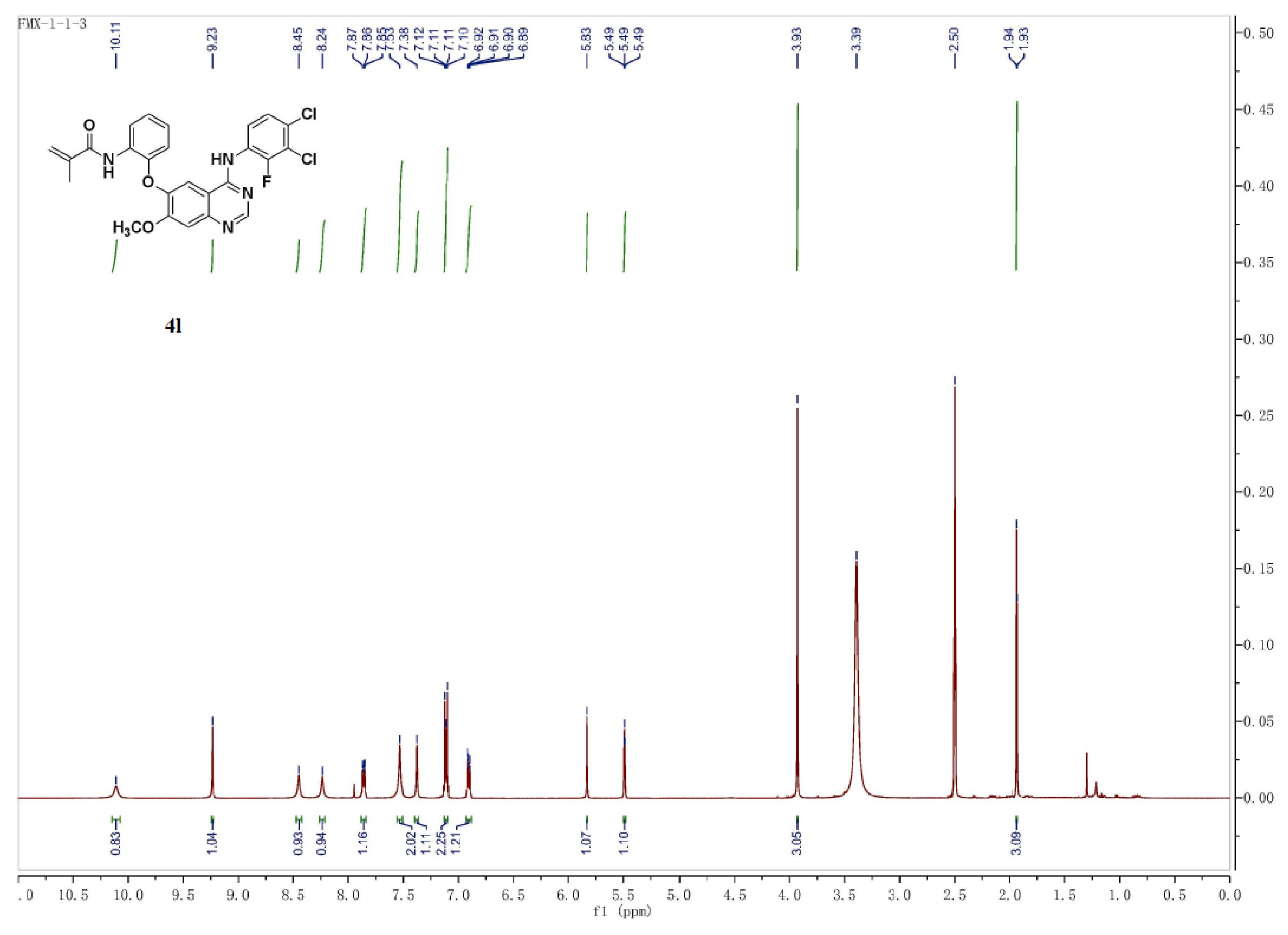

Supplement: Supplementary file 33 — 1H NMR spectrum of 4l [file turkjchem-46-3-849s33.tif]

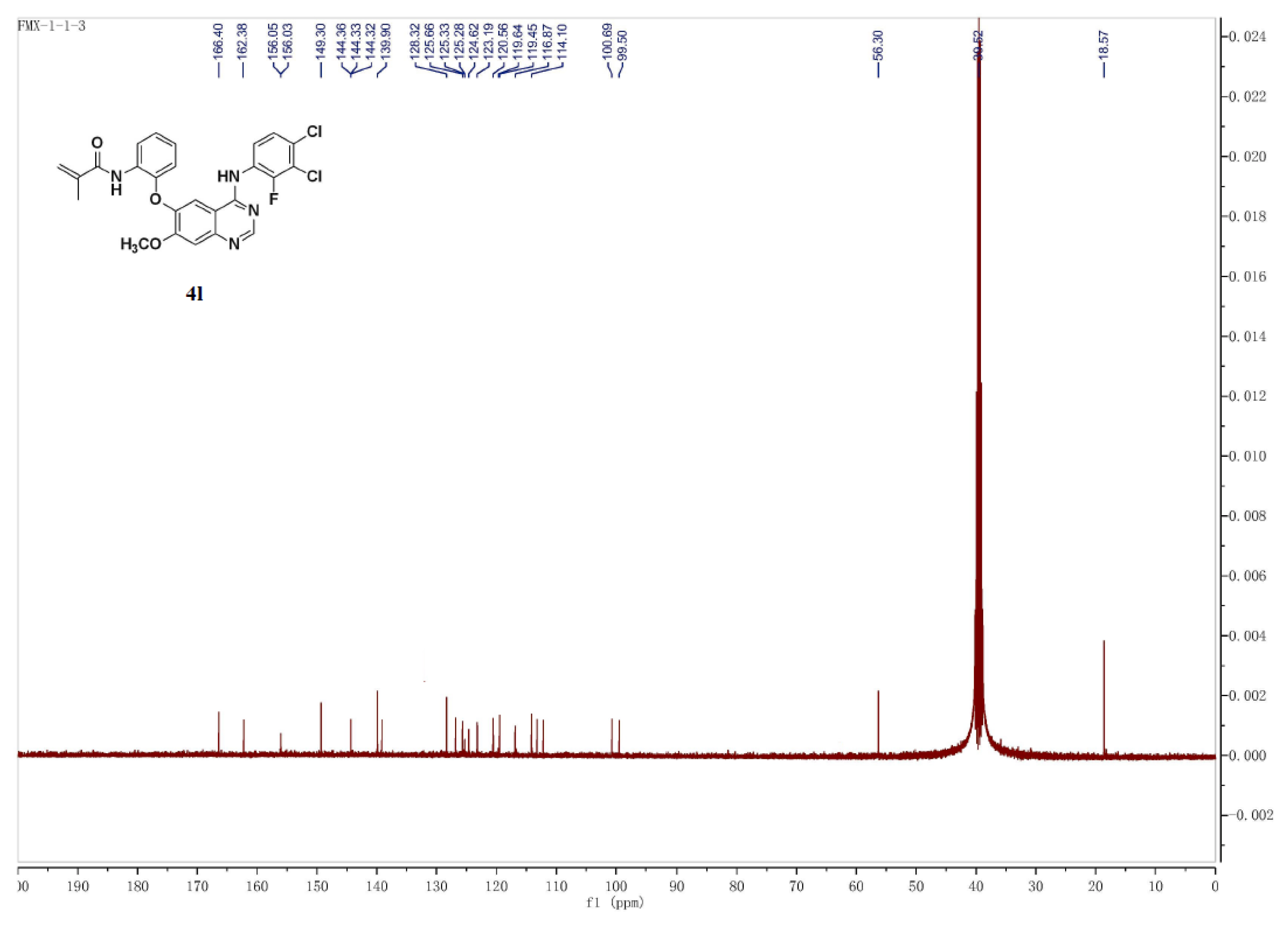

Supplement: Supplementary file 34 — 13C NMR spectrum of 4l [file turkjchem-46-3-849s34.tif]

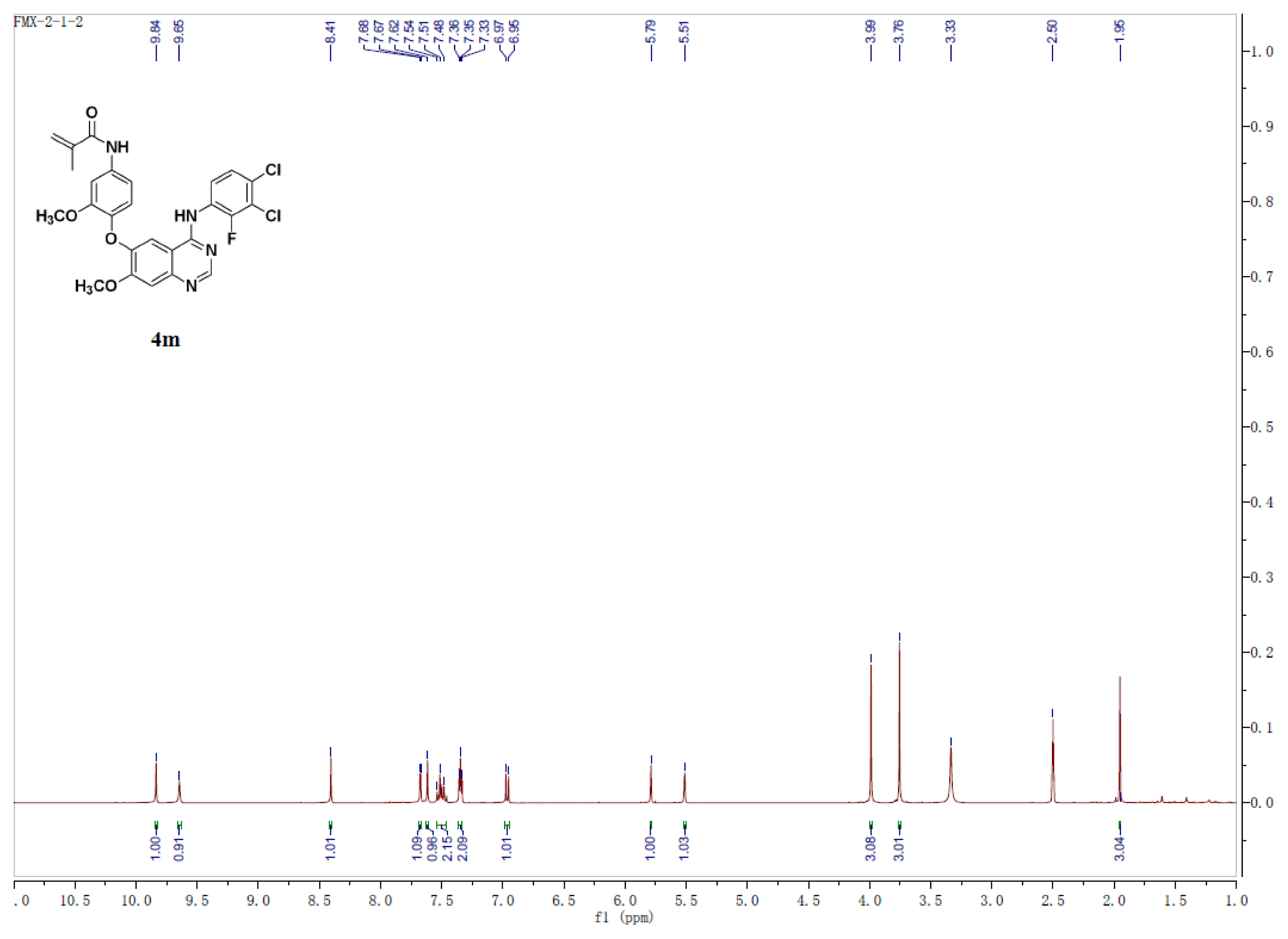

Supplement: Supplementary file 35 — 1H NMR spectrum of 4m [file turkjchem-46-3-849s35.tif]

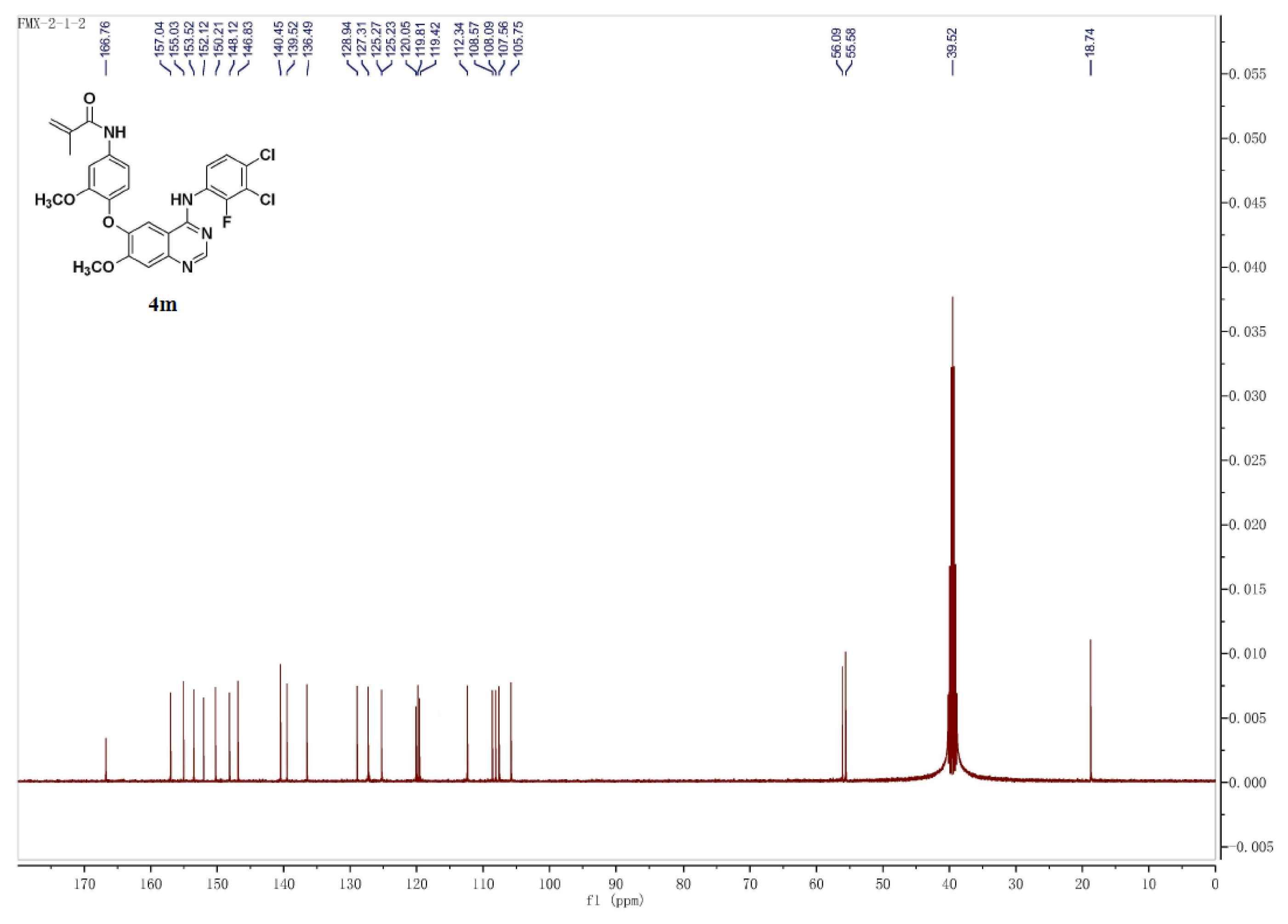

Supplement: Supplementary file 36 — 13C NMR spectrum of 4m [file turkjchem-46-3-849s36.tif]

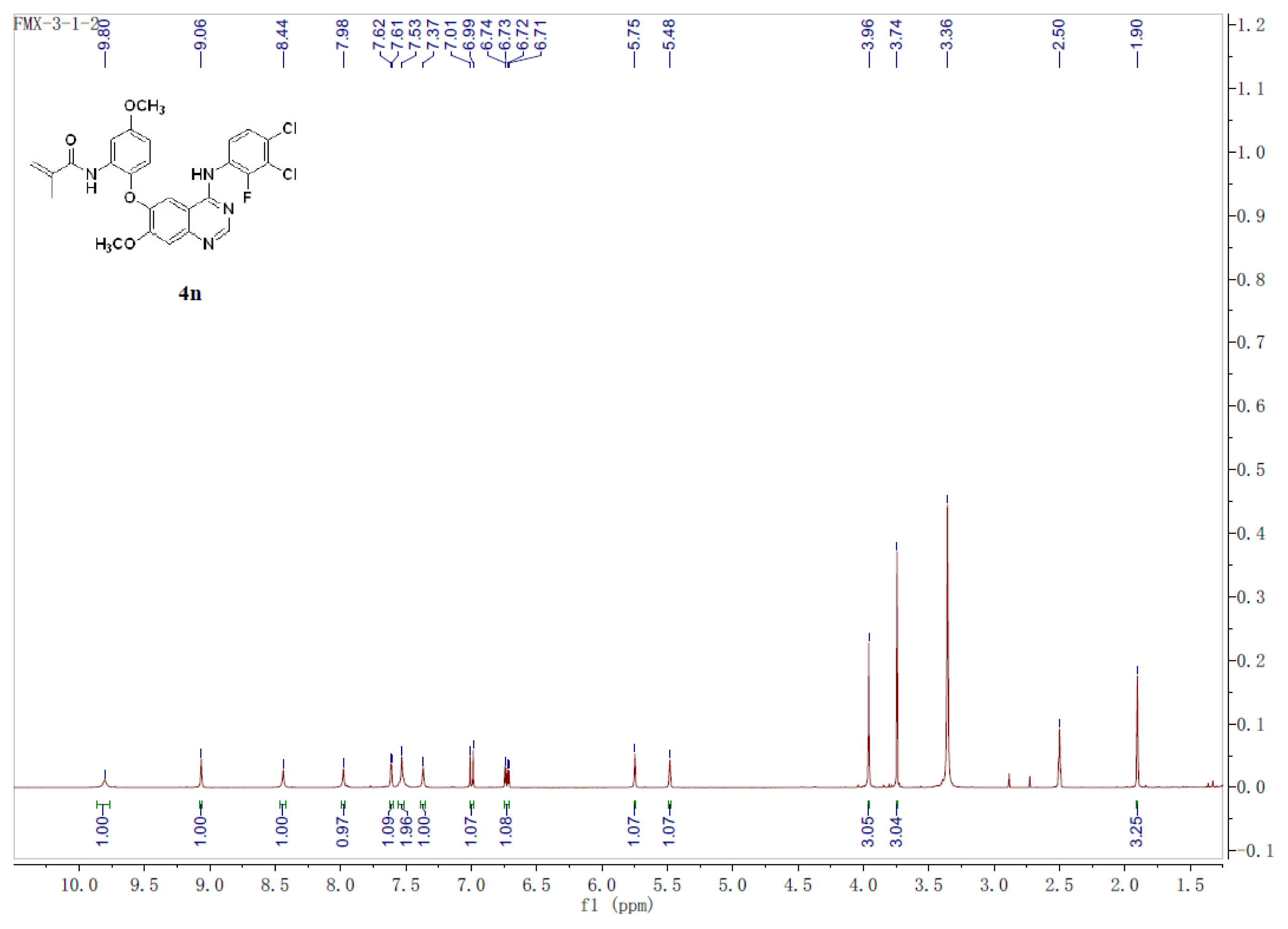

Supplement: Supplementary file 37 — 1H NMR spectrum of 4n [file turkjchem-46-3-849s37.tif]

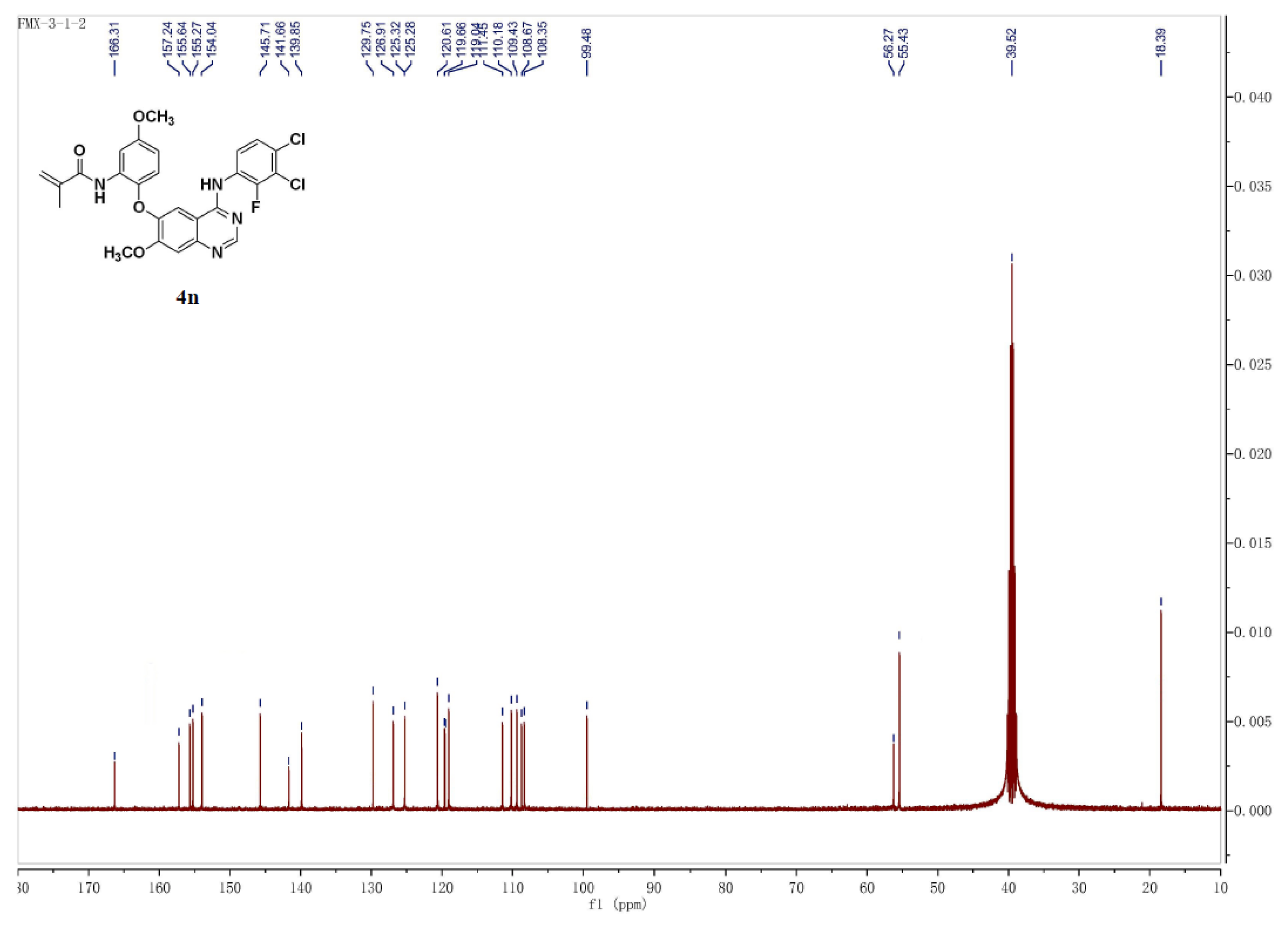

Supplement: Supplementary file 38 — 13C NMR spectrum of 4n [file turkjchem-46-3-849s38.tif]

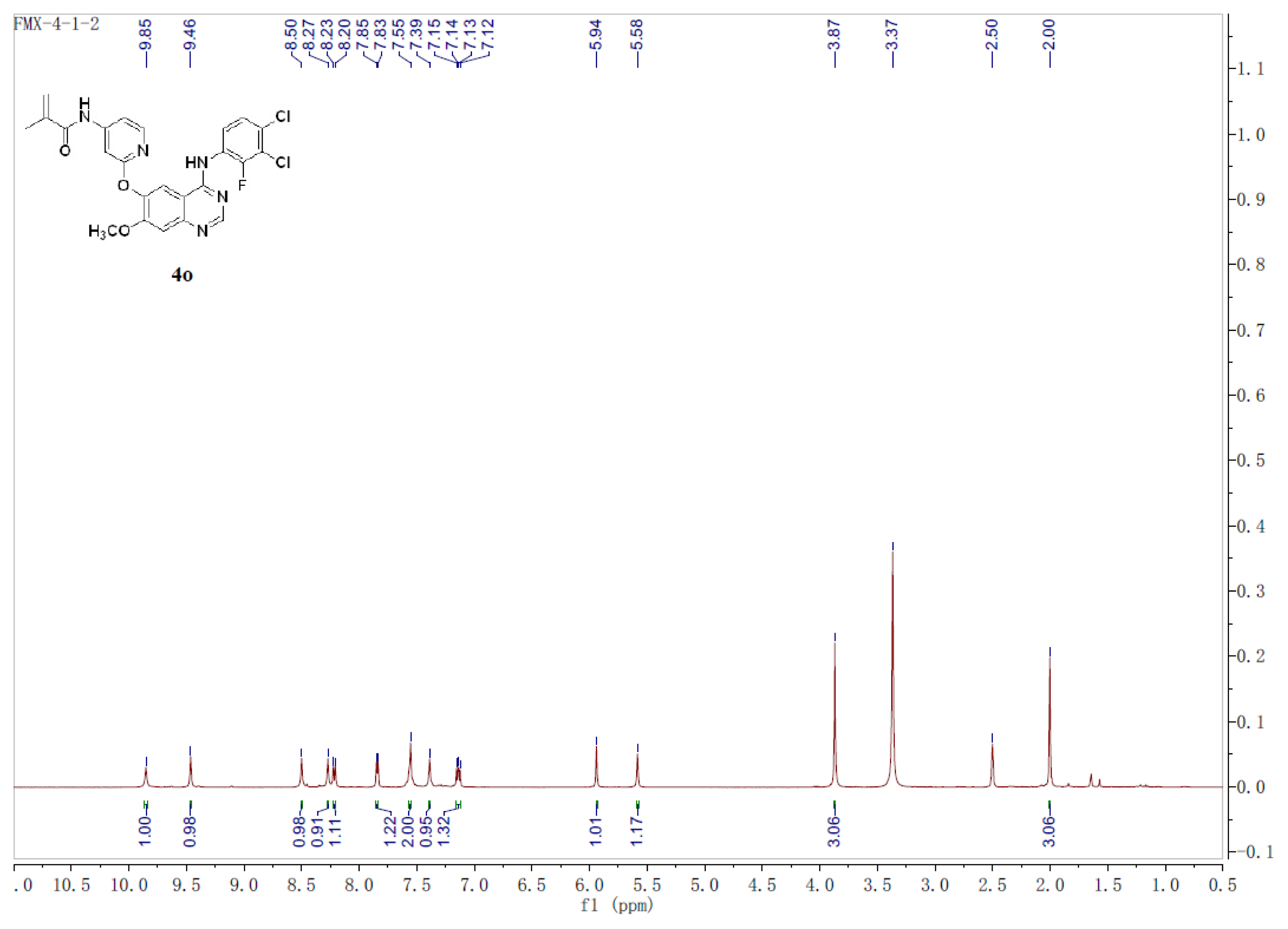

Supplement: Supplementary file 39 — 1H NMR spectrum of 4o [file turkjchem-46-3-849s39.tif]

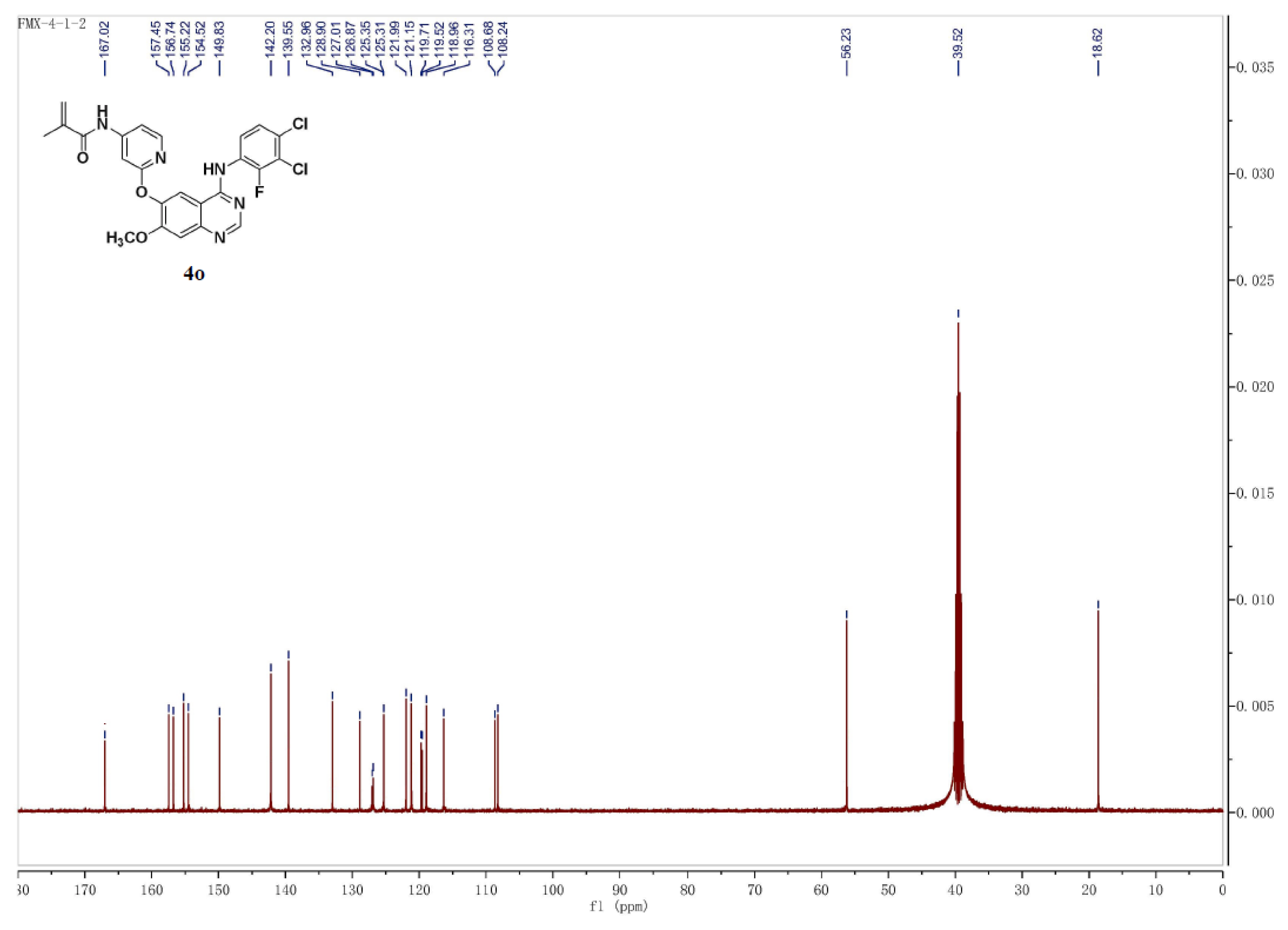

Supplement: Supplementary file 40 — 13C NMR spectrum of 4o [file turkjchem-46-3-849s40.tif]

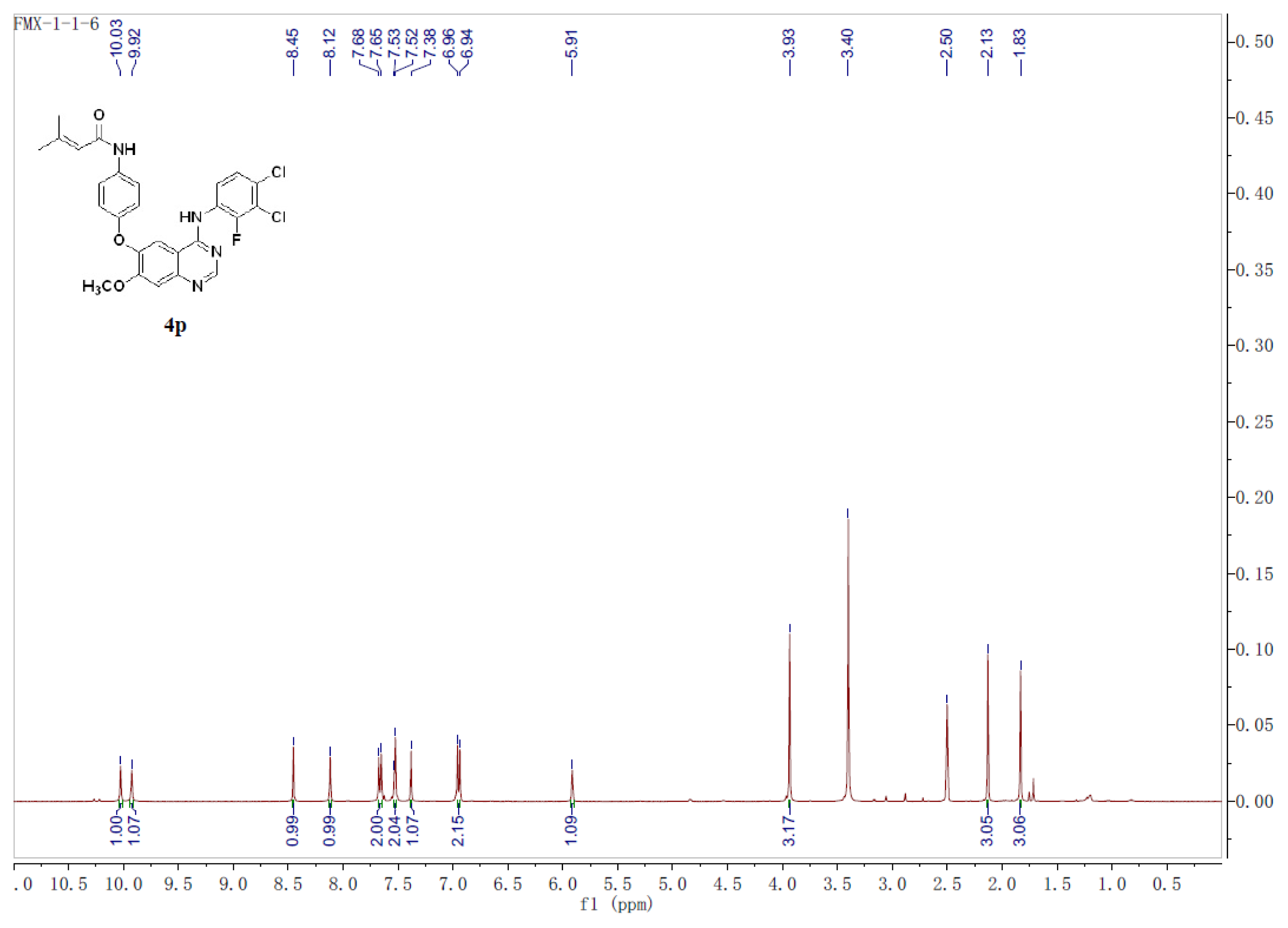

Supplement: Supplementary file 41 — 1H NMR spectrum of 4p [file turkjchem-46-3-849s41.tif]

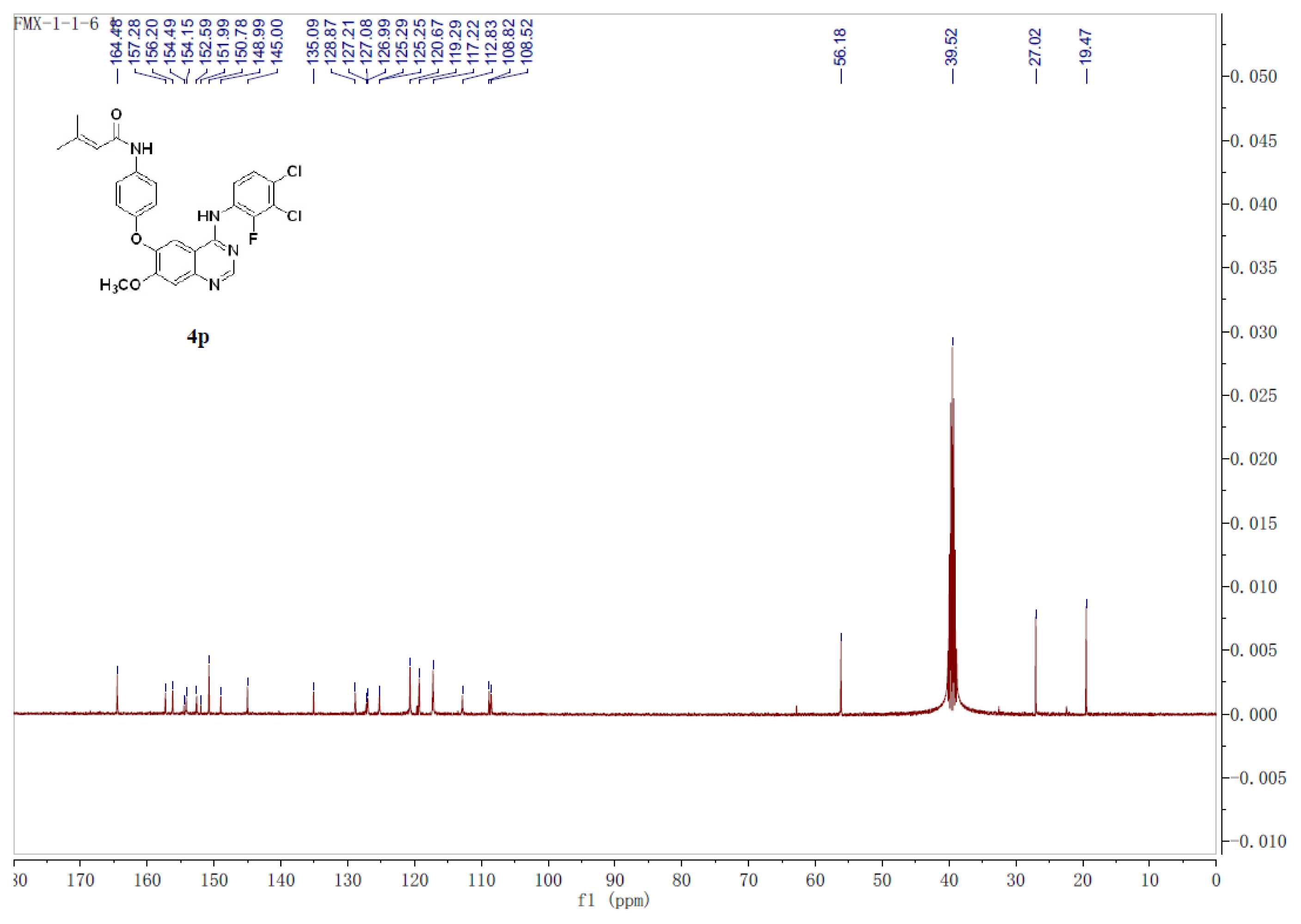

Supplement: Supplementary file 42 — 13C NMR spectrum of 4p [file turkjchem-46-3-849s42.tif]

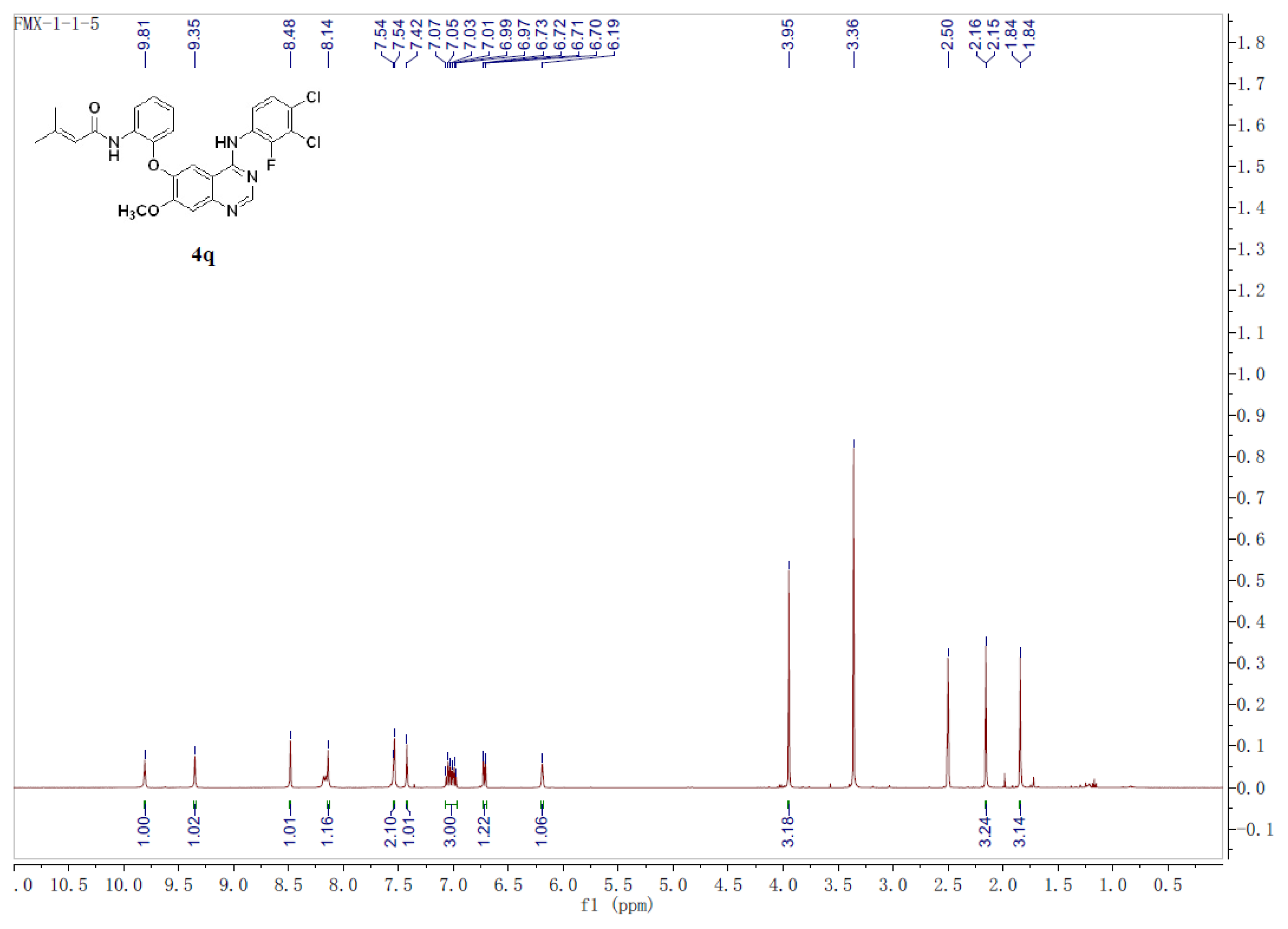

Supplement: Supplementary file 43 — 1H NMR spectrum of 4q [file turkjchem-46-3-849s43.tif]

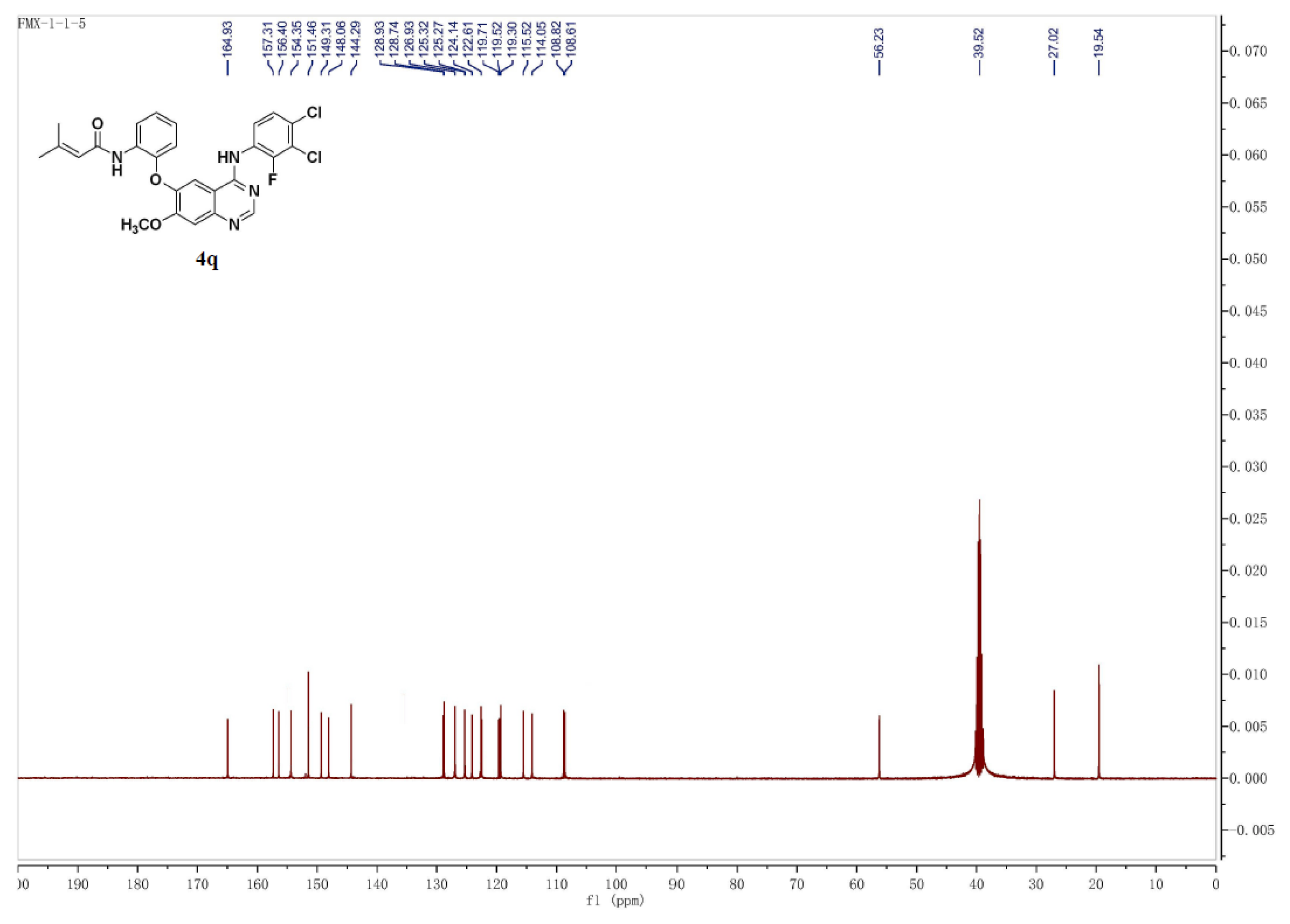

Supplement: Supplementary file 44 — 13C NMR spectrum of 4q [file turkjchem-46-3-849s44.tif]

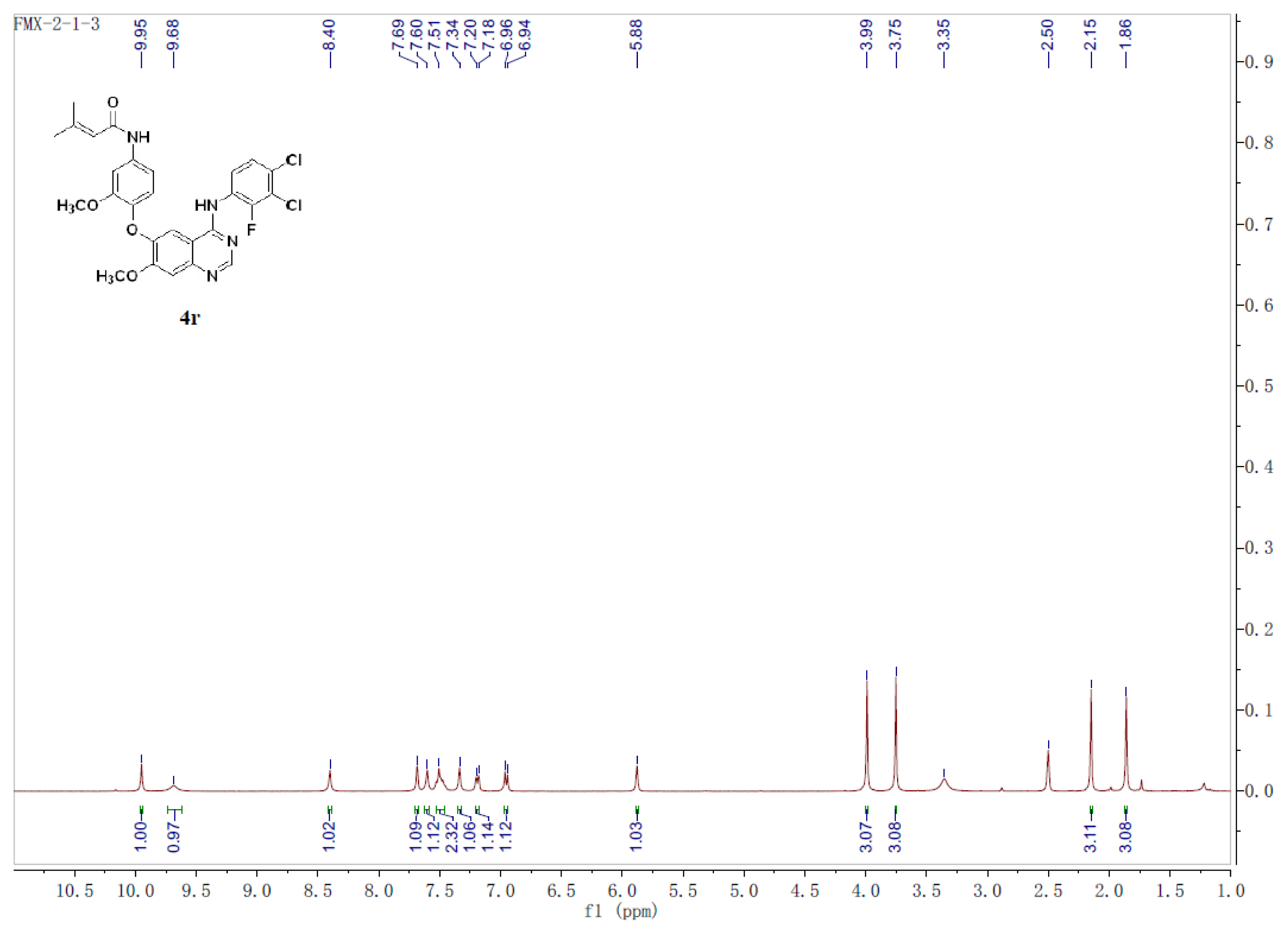

Supplement: Supplementary file 45 — 1H NMR spectrum of 4r [file turkjchem-46-3-849s45.tif]

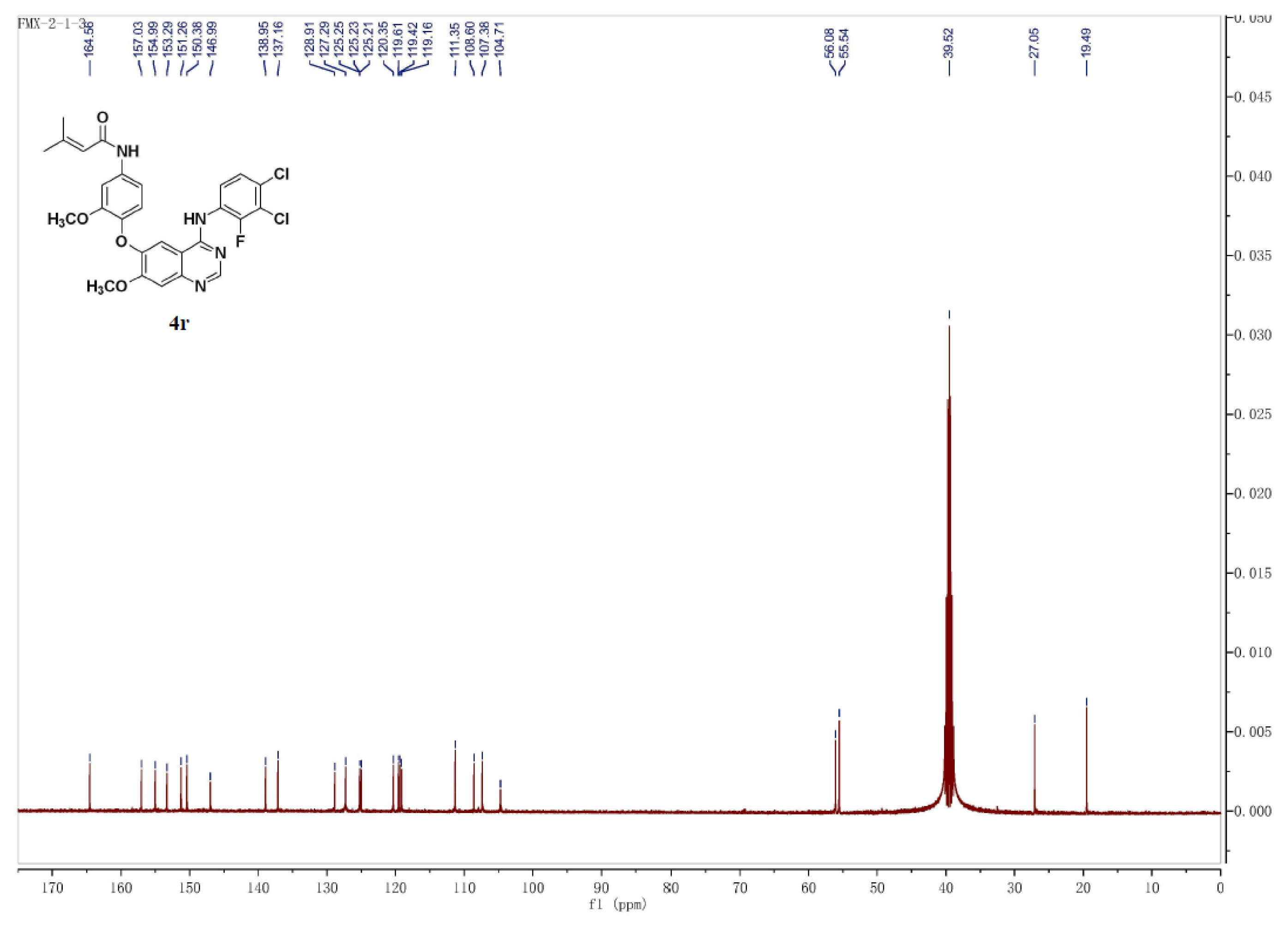

Supplement: Supplementary file 46 — 13C NMR spectrum of 4r [file turkjchem-46-3-849s46.tif]

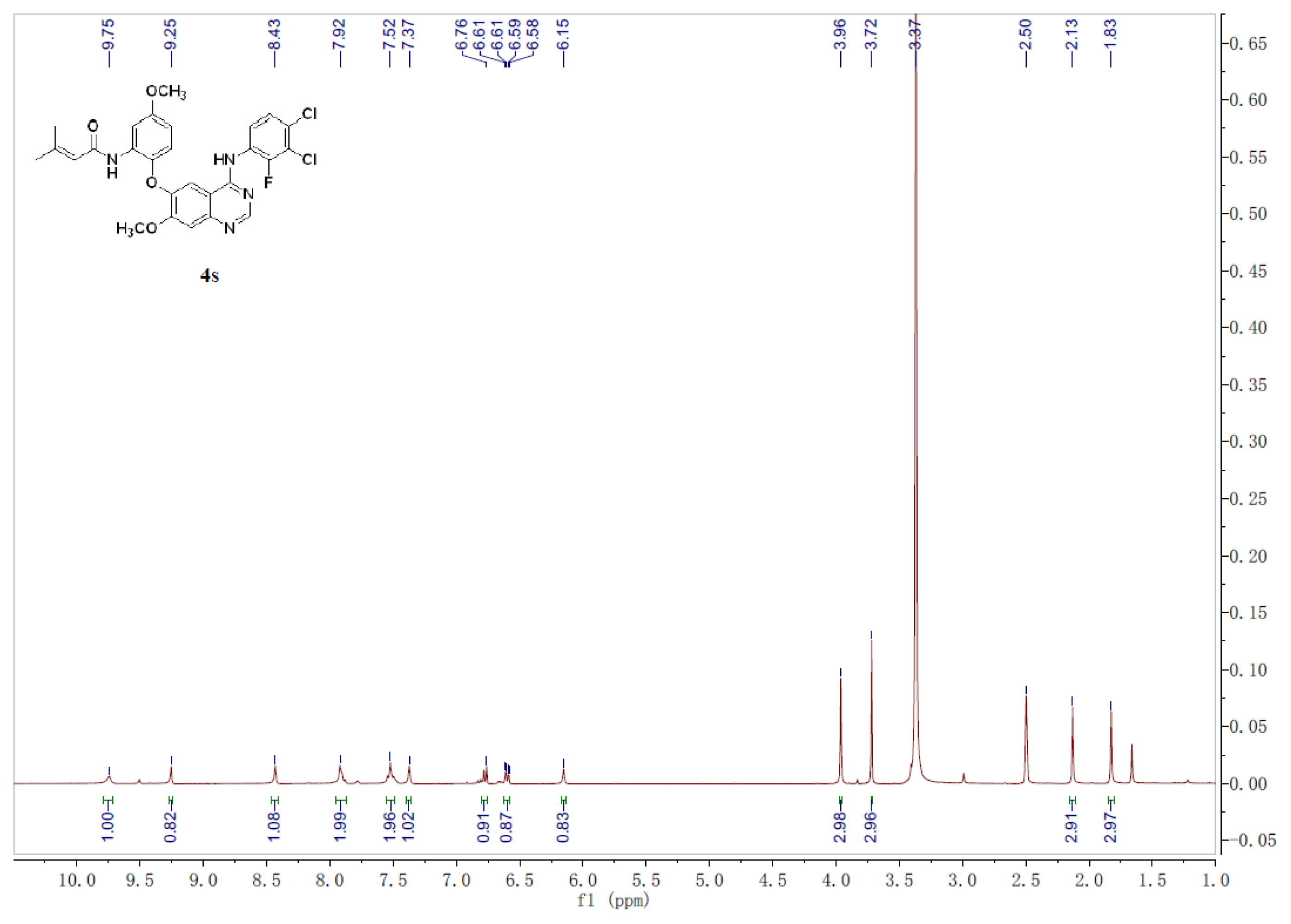

Supplement: Supplementary file 47 — 1H NMR spectrum of 4s [file turkjchem-46-3-849s47.tif]

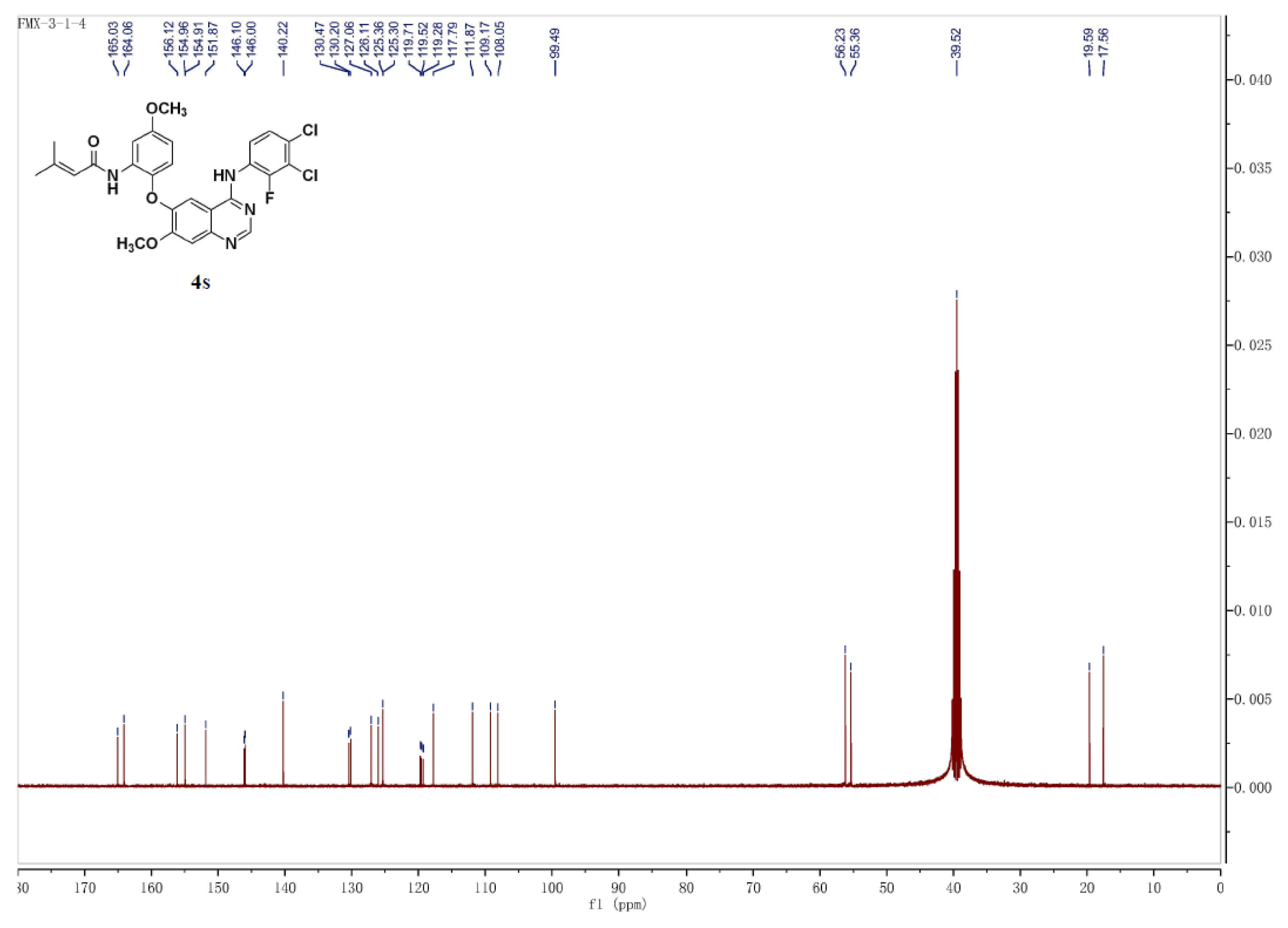

Supplement: Supplementary file 48 — 13C NMR spectrum of 4s [file turkjchem-46-3-849s48.tif]

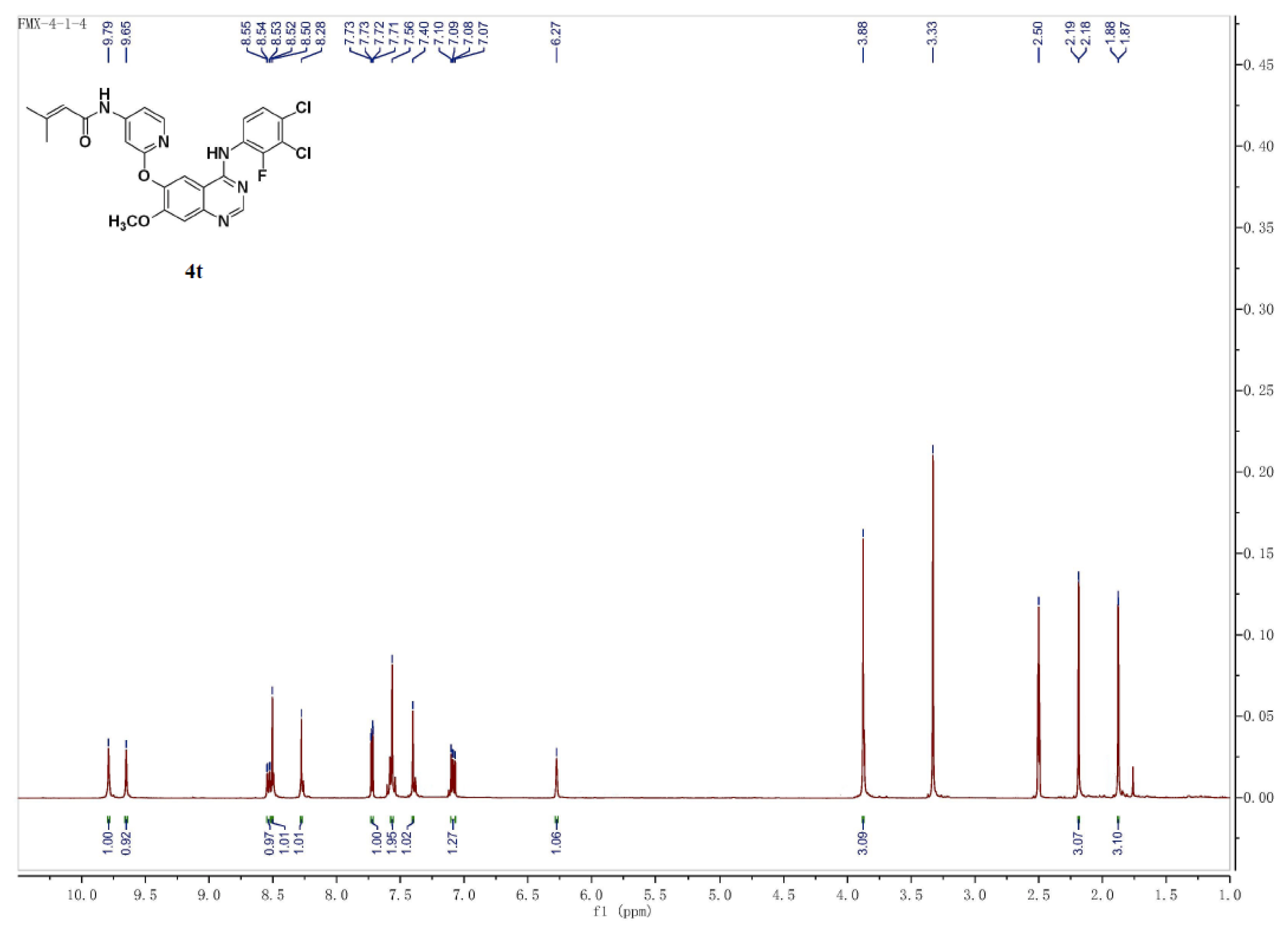

Supplement: Supplementary file 49 — 1H NMR spectrum of 4t [file turkjchem-46-3-849s49.tif]

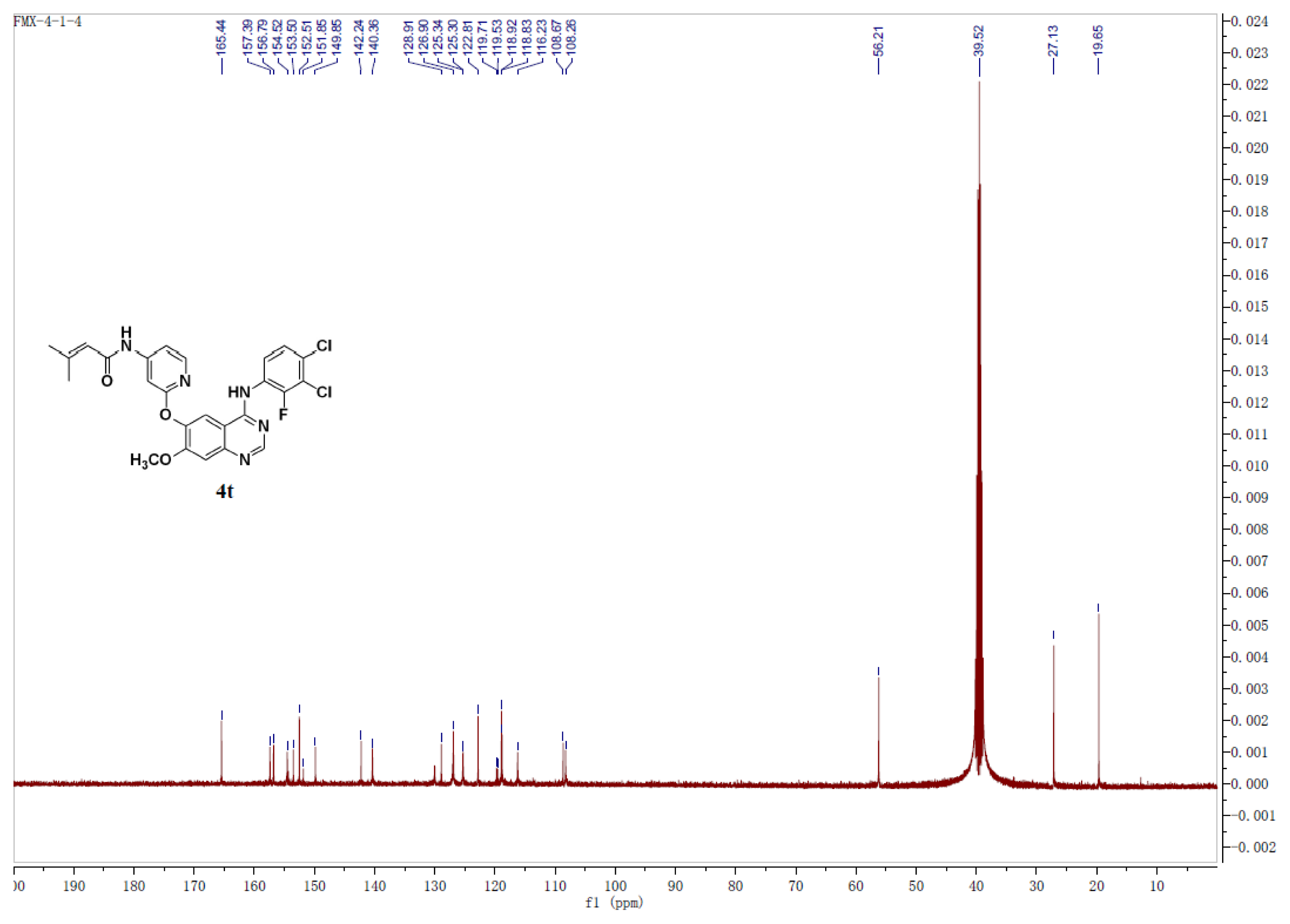

Supplement: Supplementary file 50 — 13C NMR spectrum of 4t [file turkjchem-46-3-849s50.tif]
